# In silico insights on diverse interacting partners and phosphorylation sites of respiratory burst oxidase homolog (Rbohs) gene families from Arabidopsis and rice

**DOI:** 10.1186/s12870-018-1378-2

**Published:** 2018-08-10

**Authors:** Gurpreet Kaur, Pratap Kumar Pati

**Affiliations:** 1 Department of Biotechnology, Guru Nanak Dev University (GNDU), Amritsar, Punjab 143005 India; 2 Present Address: Quantitative Biology Center (QBiC), University of Tuebingen, 72076 Tuebingen, Germany

**Keywords:** Plant NADPH oxidase, Rboh, EF-hands, Interacting partners, Network, Phosphorylation sites

## Abstract

**Background:**

NADPH oxidase (Nox) is a critical enzyme involved in the generation of apoplastic superoxide (O_2_^−^), a type of reactive oxygen species (ROS) and hence regulate a wide range of biological functions in many organisms. Plant Noxes are the homologs of the catalytic subunit from mammalian NADPH oxidases and are known as respiratory burst oxidase homologs (Rbohs). Previous studies have highlighted their versatile roles in tackling different kind of stresses and in plant growth and development. In the current study, potential interacting partners and phosphorylation sites were predicted for Rboh proteins from two model species (10 Rbohs from *Arabidopsis thaliana* and 9 from *Oryza sativa japonica*). The present work is the first step towards in silico prediction of interacting partners and phosphorylation sites for Rboh proteins from two plant species.

**Results:**

In this work, an extensive range of potential partners (unique and common), leading to diverse functions were revealed from interaction networks and gene ontology classifications, where majority of AtRbohs and OsRbohs play role in stress-related activities, followed by cellular development. Further, 68 and 38 potential phosphorylation sites were identified in AtRbohs and OsRbohs, respectively. Their distribution, location and kinase specificities were also predicted and correlated with experimental data as well as verified with the other EF-hand containing proteins within both genomes.

**Conclusions:**

Analysis of regulatory mechanisms including interaction with diverse partners and post-translational modifications like phosphorylation have provided insights regarding functional multiplicity of Rbohs. The bioinformatics-based workflow in the current study can be used to get insights for interacting partners and phosphorylation sites from Rbohs of other plant species.

**Electronic supplementary material:**

The online version of this article (10.1186/s12870-018-1378-2) contains supplementary material, which is available to authorized users.

## Background

Plants have developed various mechanisms to protect themselves against different stresses whether abiotic or biotic. One of them is the generation of reactive oxygen species (ROS) such as superoxide (O_2_^−^), singlet oxygen (^1^O_2_), and hydrogen peroxide (H_2_O_2_). Membrane-localized NADPH oxidases (Noxes) are the major source of ROS production in plants and transfer electrons from cytosolic NADPH/NADH to apoplastic oxygen which leads to ROS. They are the homolog of the mammalian NADPH oxidase catalytic subunit known as gp91phox [1]. Unlike animals, plant NADPH oxidase consists of two major structural elements: Respiratory burst oxidase homologue (Rboh) and Rop (Rho-like protein; a Rac homologue of plants). The first plant NADPH oxidase was identified in *Oryza sativa,* known as *OsRbohA* [2] and subsequently, more Rbohs were discovered in other plant species including dicots, monocots and lower plants [1]. Rboh proteins contain two Ca^2+^-binding EF-hand motifs in the N-terminal region, six transmembrane helices, FAD and NADPH binding domains in the C-terminal. The currently available crystal structure of OsRbohB N-terminal region (138–313 amino acid residues) has indicated the presence of two additional EF-hand-like motifs (EF-like 1 and EF-like 2) [3]. Rbohs are known to perform versatile functions in the plant reproduction, growth, development, and responses to abiotic and biotic stresses [1, 4]. Recently published in silico studies on the gene structures, regulatory elements, physico-chemical characterization, topology analysis, phylogenetics and structural analysis of Rbohs have provided critical insights into their diversity and hints to design functional genomics experiments [5–7]. Further, few experimental studies have revealed the interaction of Rbohs with various regulatory components for their functioning which involve Ca^2+^, calcium-dependent protein kinases (CDPKs), Ca^2+^/CaM-dependent protein kinase (CCaMK), Rop, extracellular ATP (eATP), phospholipase Dα1(PLD α1) and its lipid product phosphatidic acid (PA), mitogen activated protein kinase (MAPK), Nt14–3–3 h/omega1 (a member of 14–3–3 protein family) and nitric oxide [1, 8–10]. These interactions may be mediated via complex signaling networks and however, the knowledge regarding the connectivity of Rbohs with these components is still a subject worth investigation.

In addition to interacting partners, functioning of Rbohs through post-translational modification like phosphorylation via various types of protein kinases such as calcium-dependent protein kinase (CDPK), Ca^2+^ /CaM-dependent protein kinase (CCaMK), mitogen activated protein kinase (MAPK), BIK1 (receptor-like cytoplasmic kinase) has also been observed [1, 11, 12]. However, the various aspects such as potential phosphorylation sites (serine, threonine and tyrosine), their distribution, location and kinase specificities requires extensive experimental studies.

The experimental methods are very time-consuming and expensive, hence the currently available in silico approaches provide alternative cost-effective possibilities to explore the possible interacting associates and phosphorylation sites for Rbohs. In the present study, protein-protein interaction network analysis revealed potential interacting partners for Rbohs from two model plants (10 Rbohs from Arabidopsis and 9 from rice). Further, the potential phosphorylation sites were also elucidated including their distribution, location and kinase specificities and hence correlated with the experimental information wherever available as well as verified with the other EF-hand containing proteins. To the best of our knowledge, this is the first study documenting the potential interacting partners and phosphorylation sites for Rbohs in an extensive manner.

## Methods

### Sequence retrieval

Accession numbers for Arabidopsis and rice Rboh proteins were taken from a recent study from our lab [1]. 10 Rboh sequences for Arabidopsis and 9 for rice were retrieved from UniProt (http://www.uniprot.org/) in FASTA format.

### Analysis of protein-protein interaction network

Protein-protein interaction network studies were conducted using STRING v 9.1 (http://string-db.org/). The STRING (Search Tool for the Retrieval of Interacting Genes/Proteins) database retrieves the physical as well as functional interactions among proteins by integrating the information from neighbourhood, gene fusion, co-occurrence, co-expression, experiments, databases, text-mining and homology [13]. The functional interactions were analyzed by using medium confidence score, ranging from 0.4 to 1.0. Interactions with score < 0.4, 0.4 to 0.7 and > 0.7 are considered as low, high and highest confidence respectively. Three different options were used for finding number of interactors: no more than 10, 20 and 50 interactors, and the corresponding confidence scores ranged from 0.865 to 0.99, 0.8 to 0.99 and 0.659 to 0.99, respectively. More information about the functional partners was retrieved from UniProt (http://www.uniprot.org/).

### Prediction of phosphorylation sites and kinase specificity

Two programs were used to predict the putative phosphorylation sites in AtRbohs and OsRbohs: Musite (http://musite.net/) [14] and PlantPhos (http://csb.cse.yzu.edu.tw/PlantPhos/) [15]. We selected two models in Musite for predicting phosphorylating serine and threonine residues at 95% specificity level: General phospho-serine/threonine (*A. thaliana*) and General phospho-serine/threonine (Green Plants) for AtRbohs and OsRbohs, respectively. General phospho-tyrosine (Green Plants) model was used for predicting phosphorylating tyrosine residues among 19 Rbohs. Default options were employed in PlantPhos. To find any kinase specificity for the predicted sites, NetPhosK 1.0 (http://www.cbs.dtu.dk/services/NetPhosK/) [16] and KinasePhos 2.0 (http://kinasephos.mbc.nctu.edu.tw/) [17] programs were used. NetPhosK without ESS filtering method with other default options and KinasePhos with 95% prediction specificity and no specific kinase options were used.

### Extraction of experimentally verified phosphorylated sites

RLIMS-P (Rule-based Literature Mining System for Protein Phosphorylation (http://research.bioinformatics.udel.edu/rlimsp/) [18] was used to extract any phosphorylation related information regarding Rbohs and kinases in the literature. This was also further verified with manual search.

### Genome-wide analysis of EF-hand containing proteins in Arabidopsis and rice

Information regarding EF-hand containing proteins in Arabidopsis and rice were retrieved from two previous studies [19, 20]. Gene IDs and Locus IDs were converted to UniProt IDs using UniProt ID Mapping tool, TAIR and RAP-DB. Duplicates were removed manually while analysing the sequences.

### Amino acids occurrence percentage and distribution of EF-hand containing proteins in Arabidopsis and rice

Amino acid occurrence percentage and distribution were computed using Residue Frequency Summarizer tool (http://omics.pnl.gov/software/amino-acid-residue-frequency-summarizer) and EMBOSS Pepstats program (http://emboss.sourceforge.net/), respectively.

## Results

In the present study, 19 Rboh proteins (10 from Arabidopsis and 9 from rice) were retrieved (Additional file 1) and their interaction partners were determined. Further, phosphorylation sites were predicted, their distribution, location and kinase specificity were analyzed, which was correlated with the available experimental information as well as verified with genome-wide analysis of the other EF-hand containing proteins in both species.

### Analysis of protein-protein interaction network for Arabidopsis and rice Rbohs

**Fig. 1:**
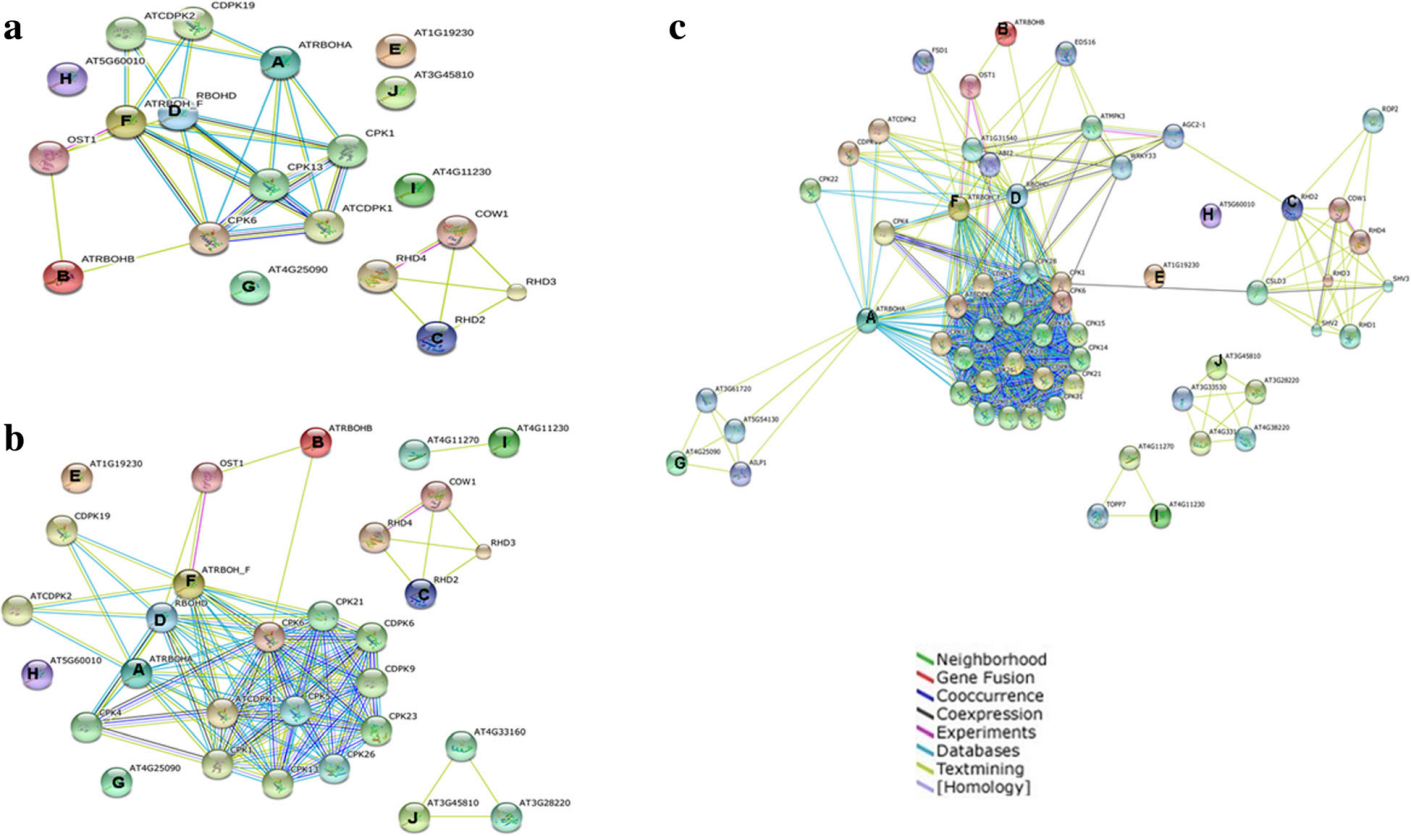
Combined network diagram showing of 10 AtRboh proteins showing potential interacting partners in evidence view with (**a**) 10 interactors (**b**) 20 interactors and (**c**) 50 interactors. Different coloured lines indicate types of evidence for association. The thickness of each line indicates the strength of the association

**Fig. 2:**
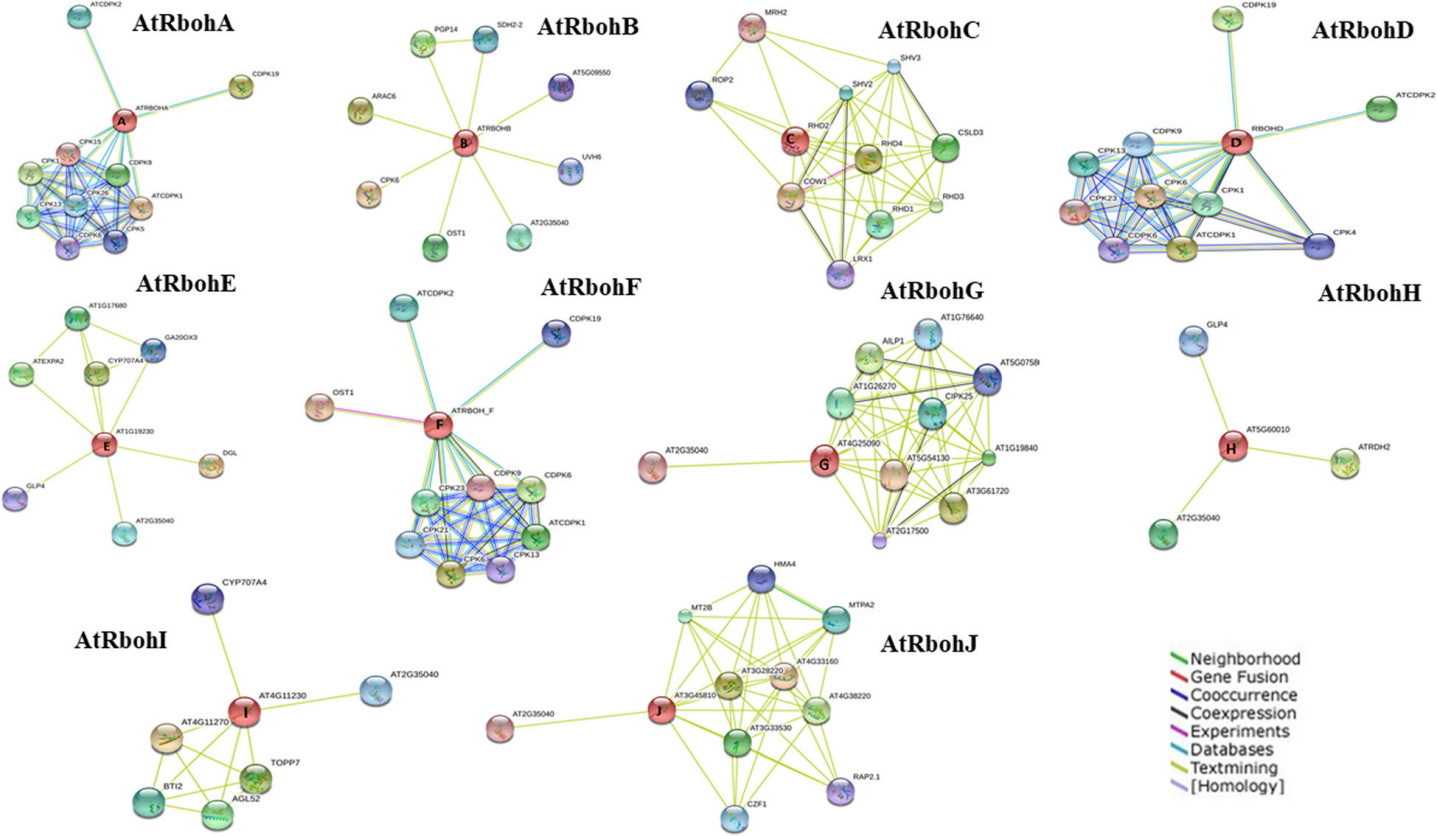
Individual network diagrams of 10 AtRboh proteins showing potential interacting partners in evidence view with 10 interactors. Different coloured lines indicate types of evidence for association. The thickness of each line indicates the strength of the association

In order to find the interaction among Rbohs and with other proteins within the respective plant species, a combined interaction network of 10 AtRbohs was constructed, which revealed that out of 10 AtRbohs, only four (AtRbohA, AtRbohB, AtRbohD and AtRbohF) were interacting with CDPKs (calcium dependent protein kinases) and two (AtRbohB and AtRbohF) with OST1 (open stomata 1) (Fig. 1a). AtRbohC was the only one who showed interaction with unique functional partners (not interacting with partners of any other AtRboh). No direct interactions among AtRbohs were observed. Few Rbohs (AtRbohE, AtRbohG, AtRbohH, AtRbohI and AtRbohJ) did not appear to interact with any partners. To get further hints, we also employed two other options “no more than 20 and 50 interactors” in the STRING database. As soon as we increased the number of interactors, we observed few partners for AtRbohI and AtRbohJ (Fig. 1b). AtRbohG which was not showing any interaction, appeared to interact with few partners common with AtRbohA (Fig. 1c). We also generated 10 interaction networks for each of the AtRbohs (Fig. 2). Six Rbohs; AtRbohA, AtRbohC, AtRbohD, AtRbohF, AtRbohG and AtRbohJ appeared to interact with 10 partners, AtRbohA, AtRbohD and AtRbohF had six common partners, AtRbohD and AtRbohF had eight, AtRbohE and AtRbohH had two; and AtRbohH and AtRbohJ had only one common partner. However, AtRbohC showed interaction with 10 unique partners, which was also obtained from combined network. AtRbohG and AtRbohJ have 9 unique partners. Four Rbohs; AtRbohB, AtRbohE, AtRbohH and AtRbohI were interacting with 8, 7, 3 and 6 partners. To get further insights, we also used two other options “no more than 20 and 50 interactors” in the STRING database. Four Rbohs (AtRbohB, AtRbohE, AtRbohH and AtRbohI) showed similar partners as observed in option using “no more than 10 interactors”. However, for another four Rbohs (AtRbohA, AtRbohC, AtRbohD and AtRbohF) more interactions were observed and for rest two (AtRbohJ and AtRbohG) additional one and two interactions were observed, respectively (Additional files 2 and 3). The details of their functional partners were further verified with UniProt (Table 1). Table 2 shows the various unique and common functional partners among AtRbohs in color coding, where unique partners are uncoloured. Further, they were grouped into various functional categories (Additional file 4).

**Table 1:** Details of identified potential functional partners of AtRboh proteins

Rbohs	Functional Partners						Types of evidence for the association							Score
	S.No.	Name	Gene ID	Description	UniProt ID	Length (aa)	Neizghborhood	Gene Fusion	Co-occurrence	Experiments	Databases	Textmining	Homology	
AtRbohA	1.	ATCDPK1	AT1G18890.1	Calcium Dependent Protein Kinase 1; induced by dehydration and high salt	Q9M9V8^a^	545					P	P		0.816
	2.	CDPK19	AT5G19450.1	Calcium Dependent Protein Kinase 19	Q42438^a^	533					P	P		0.812
	3.	CPK1	AT5G04870.1	Calcium Dependent Protein Kinase 1; phosphorylate phenylalanine ammonia lyase (PAL), a key enzyme in pathogen defense	Q06850^a^	610					P	P		0.811
	4.	CDPK9	AT5G23580.1	Calcium Dependent Protein Kinase 9	Q42396^a^	490					P	P		0.808
	5.	CPK13	AT3G51850.1	Calcium Dependent Protein Kinase 13	Q8W4I7^a^	528					P	P		0.808
	6.	ATCDPK2	AT1G35670.1	Calcium Dependent Protein Kinase 2; induced by drought and high-salt stress but not by low-temperature or heat stress; regulate ABA signal transduction	Q39016^a^	495					P	P		0.807
	7.	CPK26	AT4G38230.1	Calcium Dependent Protein Kinase 26	Q9SZM3^a^	340					P			0.8
	8.	CPK5	AT4G35310.1	Calcium Dependent Protein Kinase 5	Q38871^a^	556					P			0.8
	9.	CDPK6	AT4G23650	Calcium Dependent Protein Kinase 6; ABA regulation of guard cell S-type anion-and Ca(2+)-permeable channels and stomatal closure	Q42479^a^	529					P			0.8
	10.	CPK15	AT4G21940.1	Calcium Dependent Protein Kinase 15	O49717^a^	554					P			0.8
AtRbohB	1.	CPK6	AT2G17290.1	Calcium Dependent Protein Kinase 6; ABA regulation of guard cell S-type anion-and Ca(2+)-permeable channels and stomatal closure	Q38872^a^	544						P		0.467
	2.	ARAC6	AT4G35950.1	Rac-like 6; a member of ROP GTPases gene family-like, GTP binding protein; involved in cell polarity control during the actin-dependent tip growth of pollen tubes	Q9SBJ6^a^	197						P		0.44
	3.	PGP14	AT1G28010.1	P-GlycoProtein 14; ABC transporter B family member 14	Q9C7F2^a^	1247						P		0.408
	4.	OST1	AT4G33950.1	Open Stomata 1; regulation of stomatal aperture by ABA and acts upstream of ROS production; regulation of seed germination and root growth; dehydration stress	Q940H6^a^	362						P		0.408
	5.	AT2G35040	AT2G35040.1	AICARFT/IMPCHase bienzyme family protein; phosphoribosylaminoimidazolecarboxamide formyltransferase activity, IMP cyclohydrolase activity, catalytic activity; response to cold, purine nucleotide biosynthetic process	O64767	596						P		0.408
	6.	SDH2-2	AT5G40650.1	One of three isoforms of the iron-sulfur component of the succinate dehydrogenase complex; expressed during germination and post-germinative growth	Q8LB02^a^	280						P		0.407
	7.	UVH6	AT1G03190.2	UltraViolet Hypersensitive 6 (ATP-dependent DNA repair helicase); may negatively regulate a common response program mediated by UV damage and heat stress, that leads to tissue death and reduced chloroplast function	Q8W4M7^a^	758						P		0.406
	8.	AT5G09550	AT5G09550.1	RAB GDP-dissociation inhibitor; regulation of GTPase activity, protein transport	Q9LXC0^a^	445						P		0.405
AtRbohC	1.	COW1	AT4G34580.1	Can Of Worms1; phosphatidylinositol/phosphatidylcholine transfer protein essential for root hair tip growth	F4JLE5^a^	554						P		0.923
	2.	RHD4	AT3G51460.1	Root Hair Defective 4; phosphatidylinositol-4-phosphate phosphatase required for root hair development	Q9C5G5^a^	597						P		0.919
	3.	RHD3	AT3G13870.1	Root Hair Defective 3; required for regulated cell expansion and normal root hair development	P93042^a^	802						P		0.919
	4.	CSLD3	AT3G03050.1	Cellulose Synthase-Like D3; required for synthesis of a cell wall polysaccharide essential for root hair elongation, but not initiation	Q9M9M4^a^	1145						P		0.79
	5.	RHD1	AT1G64440.1	Root Hair Defective 1(UDP-glucose 4-epimerase 4); involved in growth and cell wall carbohydrate biosynthesis	Q9C7W7^a^	348						P		0.79
	6.	SHV2	AT5G49270.1	Shaven 2 (predicted GPI-anchored protein); involved in successfully establishing tip growth in root hairs	Q0WRJ1	663						P		0.788
	7.	SHV3	AT4G26690.1	Shaven 3 (glycerophosphoryl diester phosphodiesterase 2); cell wall cellulose accumulation and pectin linking; impacts root hair, trichome and epidermal cell development	Q9SZ11^a^	759						P		0.784
	8.	ROP2	AT1G20090.1	Rho-related protein Of Plants 2; RAC-like GTP-binding protein; its expression is stimulated by brassinosteroid treatment and inhibit light-induced stomatal opening	Q0WU07	195						P		0.72
	9.	LRX1	AT1G12040.1	Leucine-Rich repeat extensin-like protein 1; regulates root hair morphogenesis and elongation	O65375^a^	744						P		0.657
	10.	MRH2	AT3G54870.1	Morphogenesis of Root Hair 2 (Armadillo repeat-containing kinesin-like protein 1); control root hair tip growth by promoting microtubule depolymerization and limiting the accumulation of endoplasmic microtubules	Q9SV36^a^	941						P		0.651
AtRbohD	1.	CPK6	AT2G17290.1	Calcium Dependent Protein Kinase 6; ABA regulation of guard cell S-type anion-and Ca(2+)-permeable channels and stomatal closure	Q38872^a^	544					P	P		0.894
	2.	ATCDPK1	AT1G18890.1	Calcium Dependent Protein Kinase 1; induced by dehydration and high salt	Q9M9V8^a^	545				P	P	P		0.879
	3.	CDPK19	AT5G19450.1	Calcium Dependent Protein Kinase 19	Q42438^a^	533					P	P		0.873
	4.	ATCDPK2	AT1G35670.1	Calcium Dependent Protein Kinase 2; induced by drought and high-salt stress but not by low-temperature or heat stress; regulate ABA signal transduction	Q39016^a^	495					P	P		0.873
	5.	CPK1	AT5G04870.1	Calcium Dependent Protein Kinase 1; phosphorylate phenylalanine ammonia lyase (PAL), a key enzyme in pathogen defense	Q06850^a^	610				P	P	P		0.872
	6.	CPK13	AT3G51850.1	Calcium Dependent Protein Kinase 13	Q8W4I7^a^	528					P	P		0.865
	7.	CDPK9	AT5G23580.1	Calcium Dependent Protein Kinase 9	Q42396^a^	490					P	P		0.865
	8.	CPK4	AT4G09570.1	Calcium Dependent Protein Kinase 4; phosphorylates ABA-responsive transcription factors ABF1 and ABF4 *in vitro*	Q38869^a^	501				P	P	P		0.841
	9..	CDPK6	AT4G23650	Calcium Dependent Protein Kinase 6; ABA regulation of guard cell S-type anion-and Ca(2+)-permeable channels and stomatal closure	Q42479^a^	529					P	P		0.83
	10.	CPK23	AT4G04740.1	Calcium Dependent Protein Kinase 23; induced by drought and salt stress	Q9M101^a^	520					P	P		0.81
AtRbohE	1.	DGL	AT1G05800.1	Dongle (galactolipase); catalyzes the initial step of JA biosynthesis and for the biosynthesis of basal-level endogenous jasmonate in vegetative tissues, regulates leaves growth, but not essential for jasmonate biosynthesis after wounding or upon pathogen infection	Q9MA46^a^	471						P		0.436
	2.	CYP707A4	AT3G19270.1	Abscisic acid 8'-hydroxylase 4; involved in ABA catabolism	Q9LJK2^a^	468						P		0.409
	3.	ATEXPA2	AT5G05290.1	Expansin-A2; causes loosening and extension of plant cell walls by disrupting non-covalent bonding between cellulose microfibrils and matrix glucans	Q38866^a^	255						P		0.408
	4.	AT1G17680	AT1G17680.2	Transcription factor-related (putative uncharacterized protein)	Q56YL8	896						P		0.408
	5.	AT2G35040	AT2G35040.1	AICARFT/IMPCHase bienzyme family protein; phosphoribosylaminoimidazolecarboxamide formyltransferase activity, IMP cyclohydrolase activity, catalytic activity; response to cold, purine nucleotide biosynthetic process	O64767	596						P		0.408
	6.	GA20OX3	AT5G07200.1	Gibberellin 20 oxidase 3 (YAP169); biosynthesis of gibberellin that catalyzes the conversion of GA12 and GA53 to GA9 and GA20 respectively, via a three-step oxidation at C-20 of the GA skeleton	Q39112^a^	380						P		0.406
	7.	GLP4	AT1G18970.1	Germin-Like Protein 4; may play role in plant defense	P92995^a^	220						P		0.406
AtRbohF	1.	OST1	AT4G33950.1	Open Stomata 1; regulation of stomatal aperture by ABA and acts upstream of ROS production; regulation of seed germination and root growth; dehydration stress	Q940H6^a^	362				P		P		0.99
	2.	CPK6	AT2G17290.1	Calcium Dependent Protein Kinase 6; ABA regulation of guard cell S-type anion-and Ca(2+)-permeable channels and stomatal closure	Q38872^a^	544			P		P	P		0.921
	3.	CDPK6	AT4G23650	Calcium Dependent Protein Kinase 6; ABA regulation of guard cell S-type anion-and Ca(2+)-permeable channels and stomatal closure	Q42479^a^	529					P	P		0.865
	4.	ATCDPK1	AT1G18890.1	Calcium Dependent Protein Kinase 1; induced by dehydration and high salt	Q9M9V8^a^	545			P		P	P		0.856
	5.	CPK23	AT4G04740.1	Calcium Dependent Protein Kinase 23; induced by drought and salt stress	Q9M101^a^	520					P	P		0.848
	6.	ATCDPK2	AT1G35670.1	Calcium Dependent Protein Kinase 2; induced by drought and high-salt stress but not by low-temperature or heat stress; regulate ABA signal transduction	Q39016^a^	495					P	P		0.838
	7.	CPK21	AT4G04720.1	Calcium Dependent Protein Kinase 21; mediates the phosphorylation and activation of the S-type anion efflux channel SLAC1.	Q9ZSA2^a^	531					P	P		0.834
	8.	CDPK19	AT5G19450.1	Calcium Dependent Protein Kinase 19	Q42438^a^	533					P	P		0.831
	9.	CPK13	AT3G51850.1	Calcium Dependent Protein Kinase 13	Q8W4I7^a^	528					P	P		0.831
	10.	CDPK9	AT5G23580.1	Calcium Dependent Protein Kinase 9	Q42396^a^	490					P	P		0.828
AtRbohG	1.	AT5G54130	AT5G54130.2	Calcium-binding EF hand family protein	Q8GUH8	436						P		0.679
	2.	AT3G61720	AT3G61720.1	C2 domain-containing protein; calcium-dependent plant phosphoribosyltransferase family protein	Q9M366	795						P		0.679
	3.	AILP1	AT5G19140.1	Plasma membrane protein; unknown function; response to aluminum ion and auxin stimulus	Q2V367^b^, Q94BR2	234						P		0.659
	4.	AT1G19840	AT1G19840.1	Auxin-responsive family protein; auxin responsive SAUR (Small auxin-up RNA) protein	Q9FXI2	153						P		0.659
	5.	AT1G26270	AT1G26270.1	Phosphatidylinositol 4-kinase gamma 5	Q9C671^a^	630						P		0.625
	6.	CIPK25	AT5G25110.1	CBL-Interacting Protein Kinase 25	Q8W1D5^a^	488						P		0.597
	7.	AT1G76640	AT1G76640.1	Calcium-binding protein CML39; developmental and stimulus-induced expression	Q9SRE7^a^	159						P		0.567
	8.	AT5G07580	AT5G07580.1	Ethylene-responsive transcription factor ERF106; binds to the GCC-box pathogenesis-related promoter element	Q9LY05^a^	274						P		0.536
	9.	AT2G17500	AT2G17500.2	Auxin efflux carrier family protein	Q8LGC5	396						P		0.416
	10.	AT2G35040	AT2G35040.1	AICARFT/IMPCHase bienzyme family protein; phosphoribosylaminoimidazolecarboxamide formyltransferase activity, IMP cyclohydrolase activity, catalytic activity; response to cold, purine nucleotide biosynthetic process	O64767	596						P		0.408
AtRbohH	1.	ATRDH2	AT1G16460.2	Rhodanese Homologue 2 (thiosulfate/3-mercaptopyruvate sulfurtransferase 2); involved in embryo and seed development	Q24JL3^a^	342						P		0.458
	2.	AT2G35040	AT2G35040.1	AICARFT/IMPCHase bienzyme family protein; phosphoribosylaminoimidazolecarboxamide formyltransferase activity, IMP cyclohydrolase activity, catalytic activity; response to cold, purine nucleotide biosynthetic process	O64767	596						P		0.408
	3.	GLP4	AT1G18970.1	Germin-Like Protein 4; may play role in plant defense	P92995^a^	220						P		0.408
AtRbohI	1.	AT4G11270	AT4G11270.1	Transducin family protein / WD-40 repeat family protein	Q67XA6	1446						P		0.819
	2.	TOPP7	AT4G11240.1	Serine/threonine-protein phosphatase PP1 isozyme 6	P48486^a^	322						P		0.679
	3.	AGL52	AT4G11250.1	MADS-box protein AGL52; transcription factor activity	Q9SUT6	329						P		0.659
	4.	BTI2	AT4G11220.1	Virb2-Interacting Protein 2 (reticulon-like protein)	B9DHX9	271						P		0.468
	5.	AT2G35040	AT2G35040.1	AICARFT/IMPCHase bienzyme family protein; phosphoribosylaminoimidazolecarboxamide formyltransferase activity, IMP cyclohydrolase activity, catalytic activity; response to cold, purine nucleotide biosynthetic process	O64767	596						P		0.408
	6.	CYP707A4	AT3G19270.1	Abscisic acid 8'-hydroxylase 4; involved in ABA catabolism	Q9LJK2^a^	468						P		0.405
AtRbohJ	1.	AT4G33160	AT4G33160.1	Ubiquitin-protein ligase (F-box only protein 13)	Q9SMZ3^a^	457						P		0.823
	2.	AT3G28220	AT3G28220.1	Meprin and TRAF homology domain-containing protein / MATH domain-containing protein; response to salt stress	Q9LHA6	370						P		0.823
	3.	AT4G38220	AT4G38220.2	Aminoacylase, putative / N-acyl-L-amino-acid amidohydrolase	Q3E9P0^b^	433						P		0.746
	4.	AT3G33530	AT3G33530.2	Transducin family protein / WD-40 repeat family protein	Q9SRK1	1358						P		0.679
	5.	MT2B	AT5G02380.1	Metallothionein 2B; cysteine-rich protein with copper-binding activity	Q8LDX5	77						P		0.534
	6.	MTPA2	AT3G58810.1	Metal Tolerance Protein A2; member of Zinc transporter (ZAT) family. Contributes to basic cellular Zn tolerance and controls Zn partitioning, particularly under conditions of high rates of Zn influx into the root symplasm.	Q3EAH9	432						P		0.467
	7.	CZF1	AT2G40140.1	Zinc finger CCCH domain-containing protein 29; transcription factor activity; regulate salt stress	Q9XEE6^a^	597						P		0.462
	8.	HMA4	AT2G19110.1	Heavy metal transporter; involved in cadmium/zinc transport	Q0WLA3	1172						P		0.409
	9.	RAP2.1	AT1G46768.1	Ethylene-responsive transcription factor RAP2-1; binds to the GCC-box pathogenesis-related promoter element	Q8LC30^a^	153						P		0.409
	10.	AT2G35040	AT2G35040.1	AICARFT/IMPCHase bienzyme family protein; phosphoribosylaminoimidazolecarboxamide formyltransferase activity, IMP cyclohydrolase activity, catalytic activity; response to cold, purine nucleotide biosynthetic process	O64767	596						P		0.408

^a^: Reviewed UniProt ID; ^b^: Deleted entry; P: Present

**Table 2:**
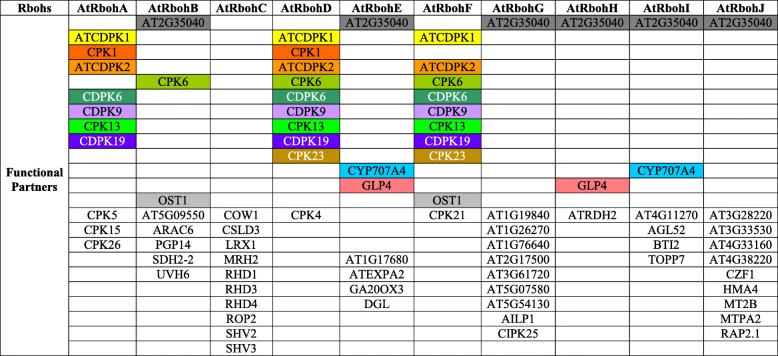
Various unique and common functional partners among AtRbohs in color coding, where unique partners are uncoloured

**Fig. 3:**
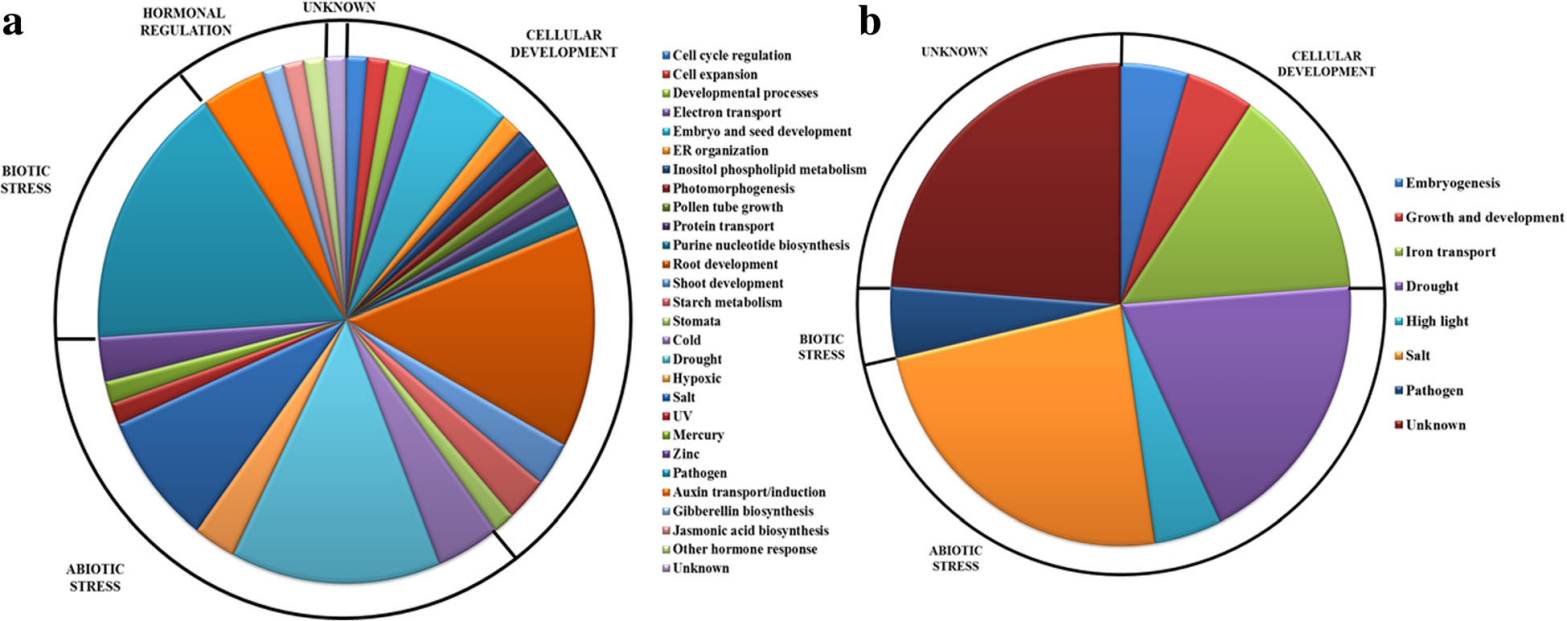
Pie chart for functional categories of identified potential interacting partners for (**a**) AtRbohs and (**b**) OsRbohs

Unlike AtRbohs, the combined network of OsRbohs involved all 9 Rbohs (Additional file 5a). As we go on increasing the number of interactors, we observed more partners for OsRbohs (Additional file 5b, c). However, like AtRbohs no direct interaction among OsRbohs was noticed. Individual networks revealed that 8 OsRbohs have overlapping functional partners (Additional file 6) except OsRbohA which has many unique partners. Unlike AtRbohs, increase in number of interactors for individual Rbohs resulted in more partners for all 9 OsRbohs (Additional files 7 and 8). The details of their functional partners were further verified with UniProt (Additional file 9). Various unique and common functional partners among OsRbohs are shown in Additional file 10. Further, they were grouped into various functional categories (Additional file 11). The various functional categories of potential interaction partners for AtRbohs and OsRbohs are represented in pie chart (Fig. 3).

### Analysis of phosphorylation sites for Arabidopsis and rice Rbohs

**Fig. 4:**
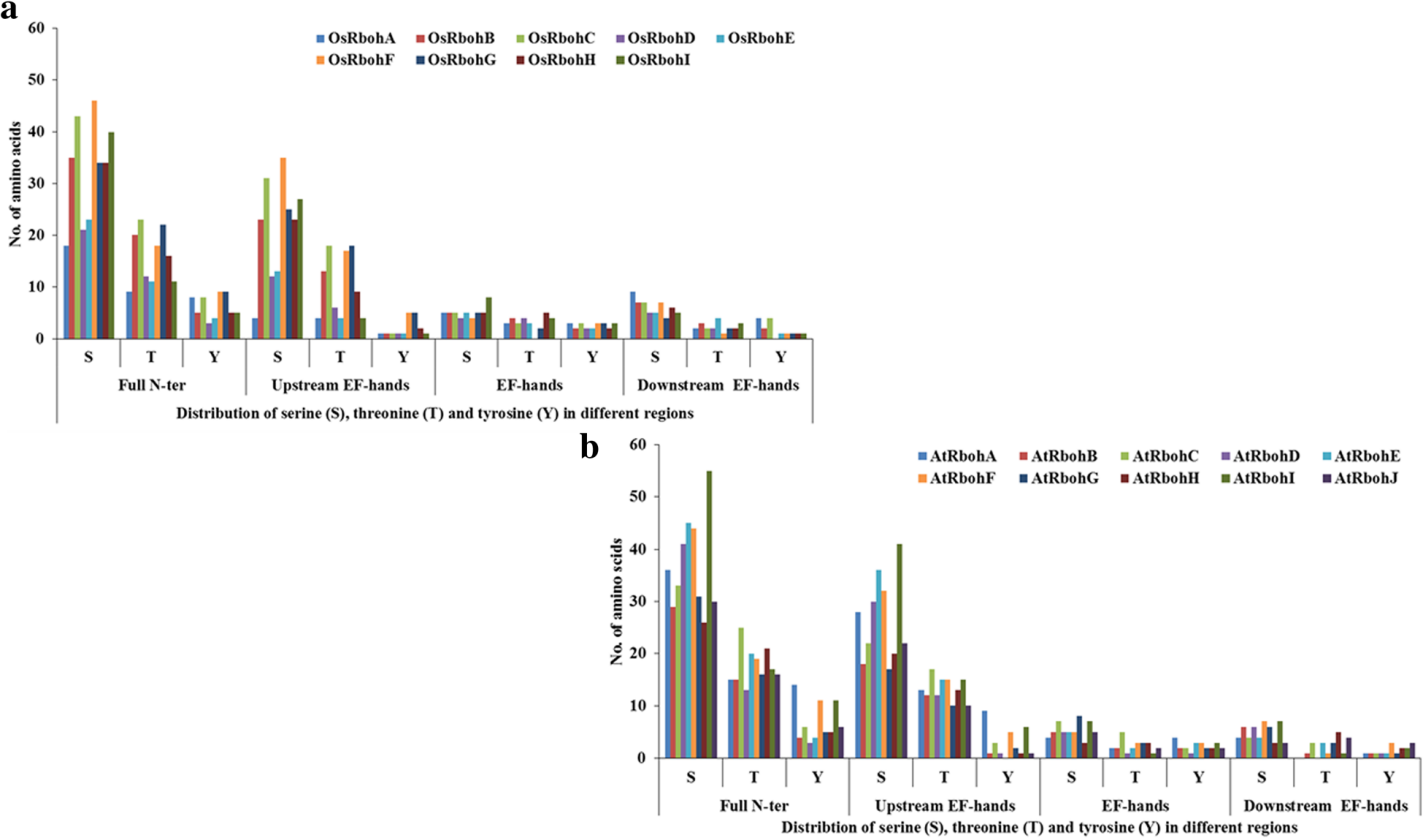
Distribution of serine (S), threonine (T) and tyrosine (Y) in different regions: upstream of EF-hands, EF-hands and downstream of EF-hands in (**a**) OsRbohs and (**b**) AtRbohs

**Fig. 5:**
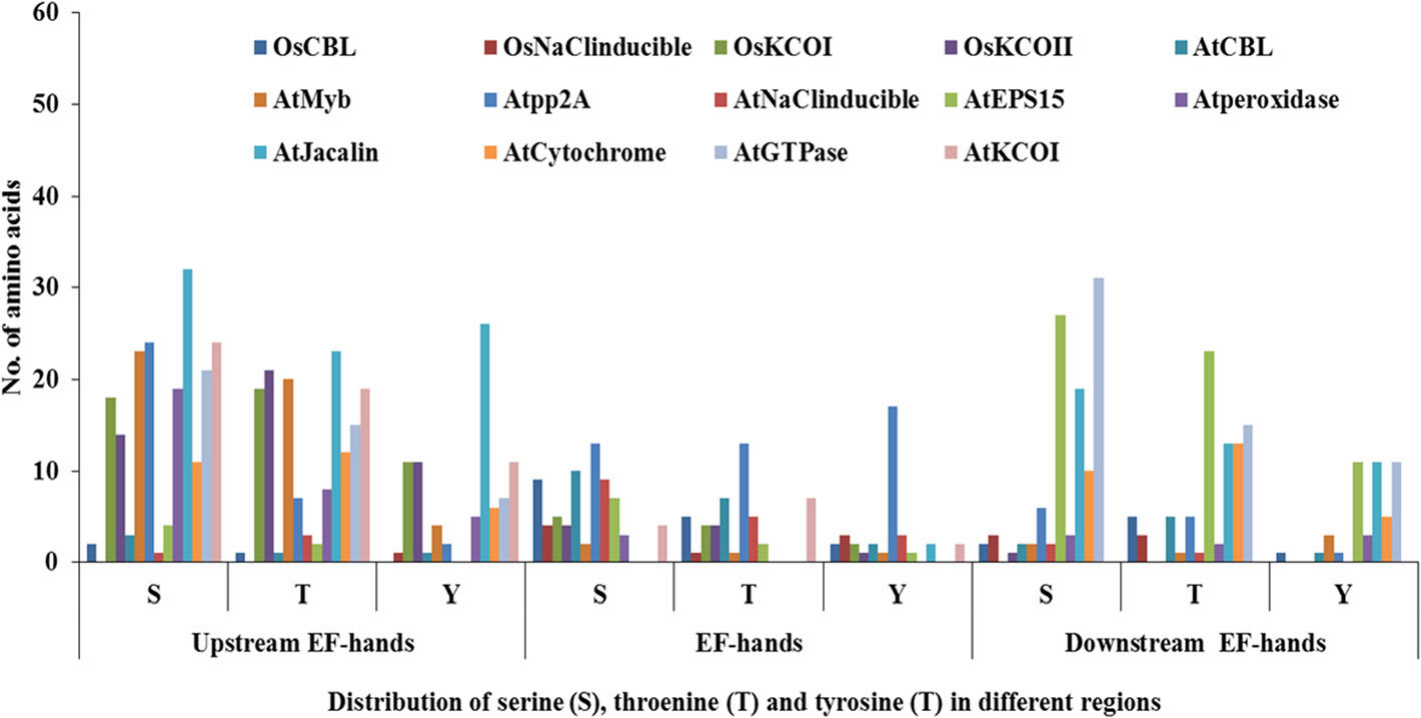
Distribution of serine (S), threonine (T) and tyrosine (Y) in different regions: upstream of EF-hands, EF-hands and downstream of EF-hands in EF-hand containing proteins of Arabidopsis and rice

**Fig. 6:**
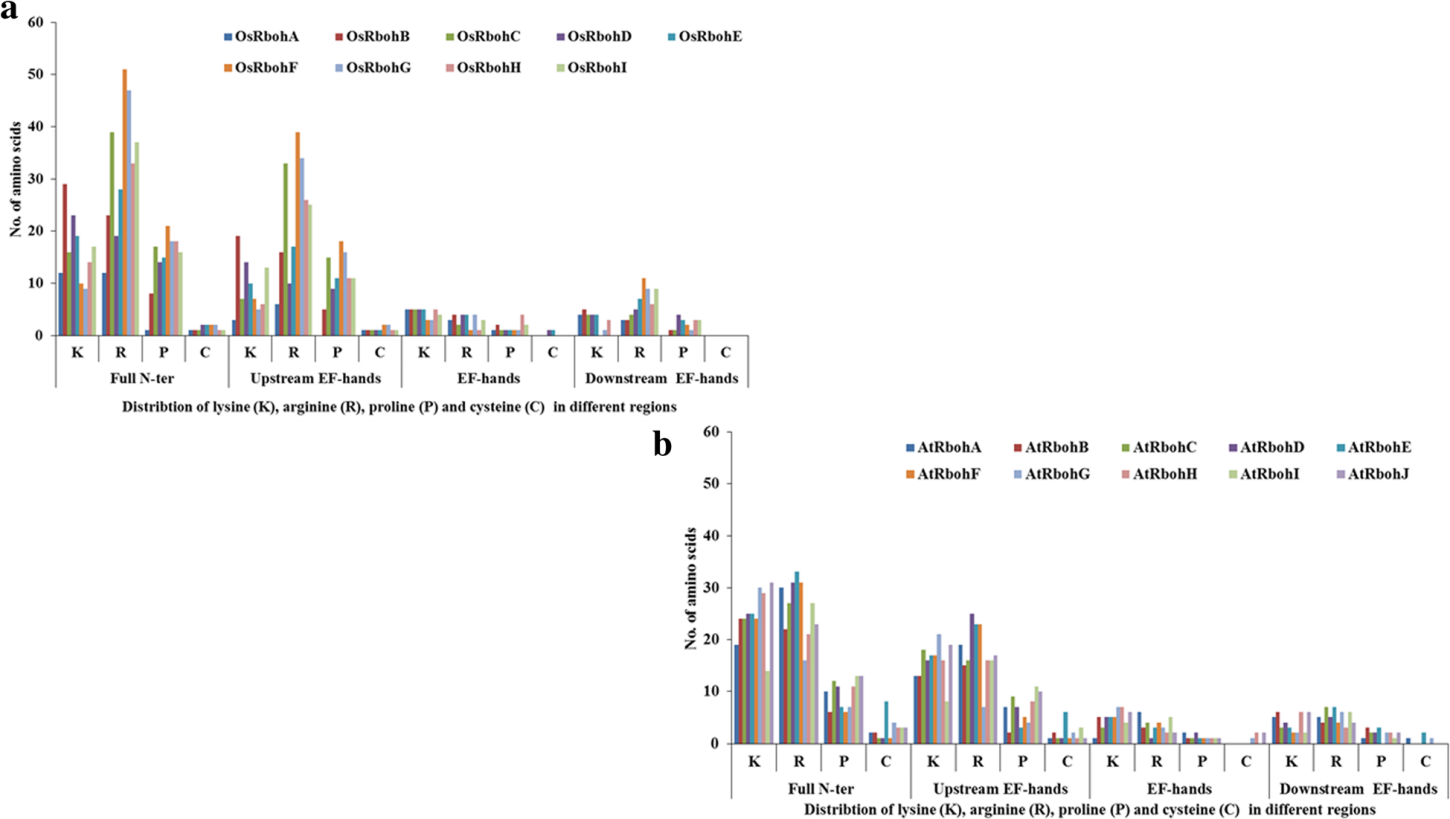
Distribution of lysine (K), arginine (R), proline (P) and cysteine (C) in different regions: upstream of EF-hands, EF-hands and downstream of EF-hands in (**a**) OsRbohs and (**b**) AtRbohs

**Fig. 7:**
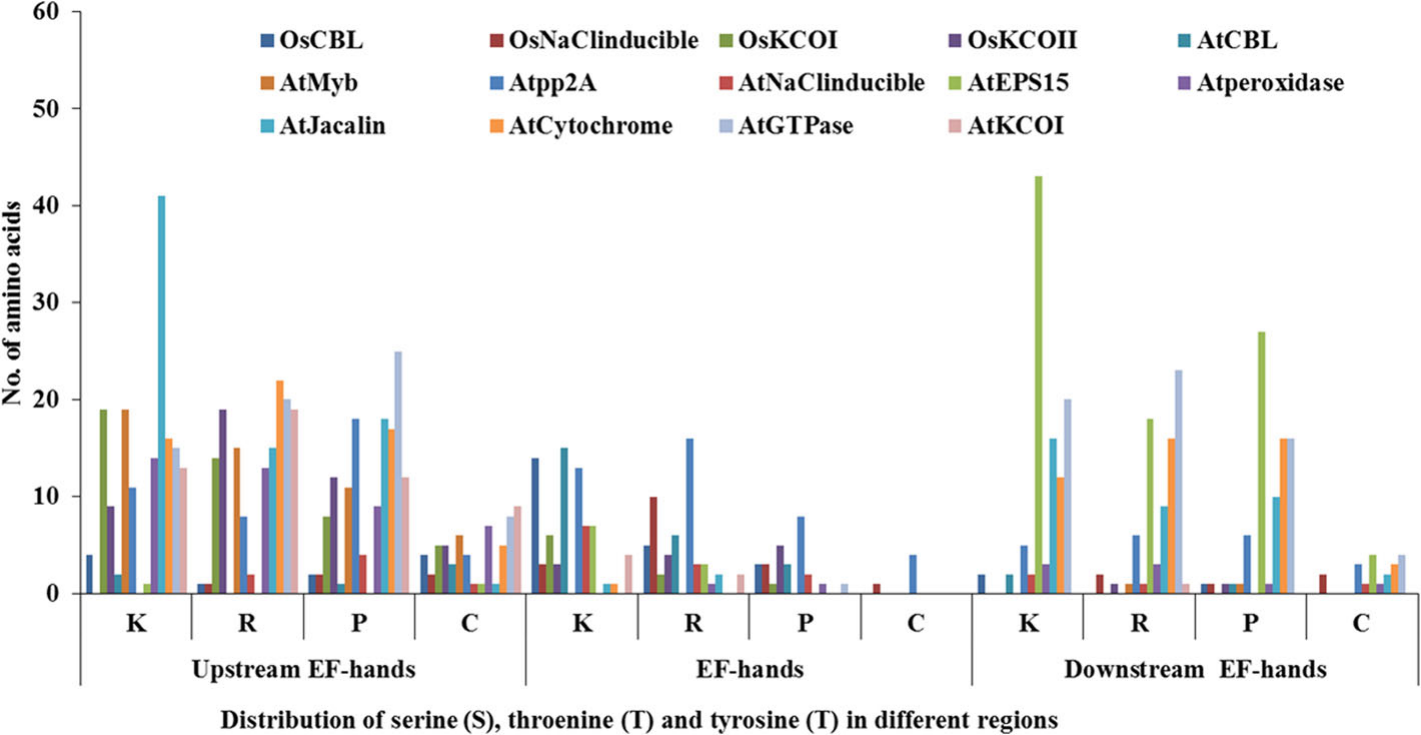
Distribution of lysine (K), arginine (R), proline (P) and cysteine (C) in different regions: upstream of EF-hands, EF-hands and downstream of EF-hands in EF-hand containing proteins of Arabidopsis and rice

Potential serine (S), threonine (T) and tyrosine (Y) phosphorylation sites were identified among 19 Rboh proteins (Table 3). Total number of phosphorylation sites predicted in AtRbohs and OsRbohs at 95% specificity level were 68 and 38, respectively. Maximum number of phosphorylation sites for AtRbohs were observed in AtRbohD whereas, in case of rice, it was OsRbohC. Further, the location of predicted sites within the Rboh proteins were identified and both the predicted and few experimentally verified sites were mapped on the multiple sequence alignment of 19 Rbohs (Additional file 12). Most of the potential phosphorylation sites were found in the N-terminal region upstream of EF-hands. For example, equivalent serine residues corresponding to S-148 and S-163 from AtRbohD were conserved in four (AtRbohA, AtRbohC, AtRbohE and AtRbohF) and all AtRbohs, respectively. Further, equivalent serine residues for S-174 from AtRbohF were found conserved among all AtRbohs while S-318 and S-322 from AtRbohC were conserved in 6 AtRbohs (AtRbohA, AtRbohD, AtRbohE, AtRbohF, AtRbohG and AtRbohI) and 3 AtRbohs (AtRbohA, AtRbohB and AtRbohD), respectively. In addition, the kinase specificities/preferences of the putative phosphorylation sites were also computed. NetPhosK and KinasePhos analysis indicated that AtRboh and OsRboh proteins possess a broad range of phosphorylation sites (Table 3). To find the abundance of S, T and Y residues in different regions of 10 AtRbohs and 9 OsRbohs, all Rbohs was divided into four regions: full N-terminal, upstream of EF-hands, EF-hands and downstream of EF-hands. The distribution patterns of S, T and Y for 9 OsRbohs and 10 AtRbohs in each region were computed (Fig. 4a, b). To verify the obtained results, the Gene IDs and Locus IDs for other EF-hand containing proteins of Arabidopsis and rice were retrieved from literature and converted into protein IDs. Overall, 230 Arabidopsis and 211 rice EF-hand containing proteins were obtained. Further, distributions of S, T and Y were analyzed for the representative proteins including 10 from Arabidopsis and 4 from rice (Fig. 5). Similarly other important residues which include lysine (K), arginine (R), proline (P) and cysteine (C) were also analyzed in OsRbohs and AtRbohs (Fig. 6a, b) as well as in the representative proteins (Fig. 7).

**Table 3:**
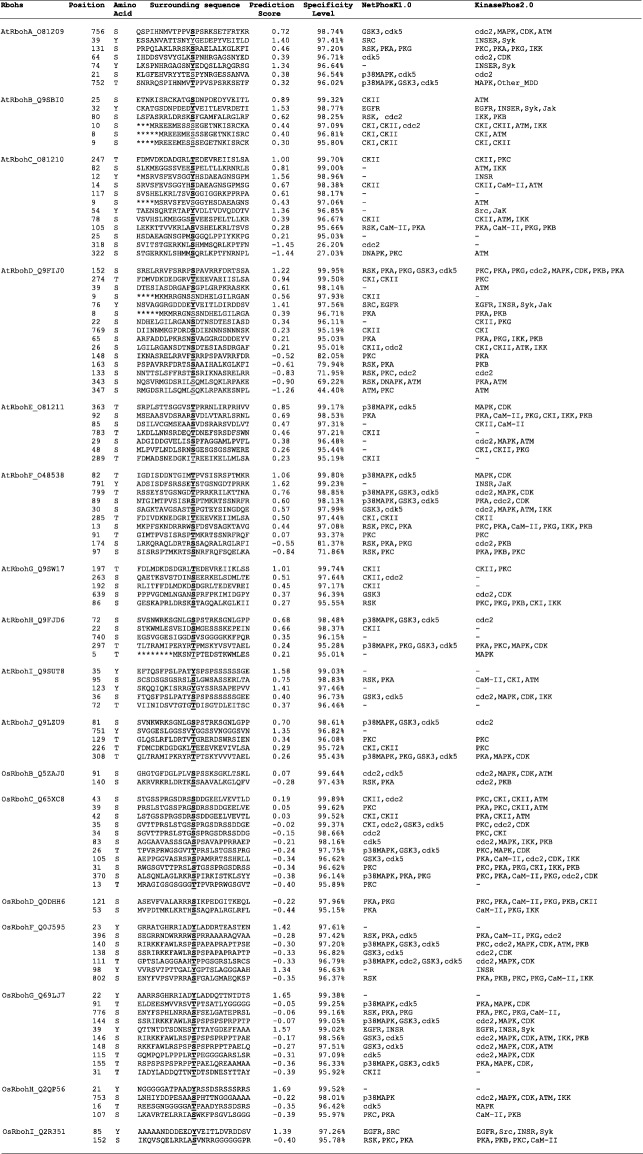
Predicted S/T/Y phosphorylation sites of 19 Rbohs from Musite webserver are underlined and highlighted in cyan. Sites verified from PlantPhos are shown in bold. Experimentally validated sites are highlighted in green

*Abbreviations*: *GSK3* Glycogen synthase kinase 3, *cdk5* cyclin dependent kinase 5, *SRC* Tyrosine kinase, *RSK* 90 kDa ribosomal S6 kinase, *PKA* cyclic AMP-dependent protein kinase, *PKC* protein kinase C, *PKG* cyclic GMP-dependent protein kinase, *p38MAPK* p38 Mitogen-activated protein kinase, *EGFR* Epidermal growth factor receptor, *CaM-II* Calmodulin-dependent protein kinase II, *cdc2* cell division control protein 2, *CKII* Casein Kinase II, *DNAPK* DNA activated protein kinase, *ATM* Ataxia Telangiectasia-Mutated, *PKC* Protein kinase C, *CDK* Cyclin-dependent kinase, *CKI* Casein kinase I, *p34cdc2* p34 cell division control protein, *MAPK* Mitogen-activated protein kinase, *IKK* IkappaB kinase, *PKB* Protein kinase B, *INSR* Insulin receptor, *Ab1* Abelson murine leukemia virus oncoprotein (tyrosine kinase), *Syk* Spleen tyrosine kinase, *Jak* Janus kinase

## Discussion

Plant NADPH oxidases, also known as respiratory burst oxidase homologs (Rbohs), are critical players in the production of reactive oxygen species (ROS) and play diverse roles [1, 4, 8]. In the recent past, there has been rapidly growing interest to study different aspects of Rbohs using in silico approaches [5–7, 21]. Analysis of regulatory mechanisms, for e.g. interaction with different partners and post-translational modification such as phosphorylation are believed to provide vital clues towards functional multiplicity of Rboh proteins. In the present study, protein-protein interaction network analysis revealed potential interacting partners for Arabidopsis and rice Rbohs. In addition, the potential phosphorylation sites were identified with their distribution, location and kinase specificities as well as correlated with experimental data wherever available. This was further verified with the other EF-hand containing proteins throughout the both genomes.

### Analysis of protein-protein interaction network for Arabidopsis and rice Rbohs

Rbohs are known to mediate diverse functions and are implicated in many signal transduction pathways involving interactions with different components involved in their regulation such as Ca^2+^, protein kinases (CDPKs; Ser/Thr protein kinases having a Ca^2+^ − binding calmodulin-like domain, OST1, CBL/CIPK; calcineurin B-Like calcium sensors–interacting protein kinase) and Rop; Rac of plants [1]. These interactions may be mediated in the form of well-regulated networks and the information regarding the connectivity of Rbohs with these components is still the subject of investigation. Therefore, it is imperative to decipher these interactions among Rbohs and with other genes/proteins. Hence, we performed protein-protein interaction network analysis using the STRING database to find any functional associations among Rbohs and specific Rboh with other proteins. The STRING stands for Search Tool for the Retrieval of Interacting Genes/Proteins that retrieves the physical and functional interactions among proteins by integrating the information from a variety of evidence types (neighbourhood, gene fusion, co-occurrence, co-expression, experiments, databases, text-mining and homology) [13]. In the present work, the total number of partners showing interactions with AtRbohs and OsRbohs were 59 and 19, respectively. Rboh proteins with a high degree of connectivity i.e. connected to many other proteins would act as a central point involved in supervising communication in a network. On the other hand, the nodes that are not connected indicates no interaction with other proteins and hence, involved in an indirect role. A combined STRING network analysis of 10 AtRbohs predicted that various types of CDPKs were common partners for four Rbohs (AtRbohA, AtRbohB, AtRbohD and AtRbohF). However, individual network analysis of each AtRboh revealed more details of partners. AtRbohA and AtRbohD were predicted to interact with 10 CDPKs, AtRbohB with one CDPK and AtRbohF with nine CDPKs. AtRbohA, AtRbohD and AtRbohF had six common CDPKs indicating the diversity as well as overlapping in their functions. Except AtRbohA whose function is still unknown, AtRbohD and AtRbohF are known to play multiple roles [1]. The reason might be the differences in the substrate specificities of AtCDPKs which may lead to their functional multiplicity [22, 23]. However, there are no experimentally validated interactions of CDPK with AtRbohA, AtRbohB and AtRbohF, but with that of AtRbohD have been recently reported [24]. It has been suggested that the flg22 induced activation of CPK5 leading to the phosphorylation of AtRbohD. In the predicted networks, CPK5 was found to interact with AtRbohD at confidence score of 0.8 when we used “no more than 20 and 50 interactors” options. In addition, CPK5 was also observed to interact with AtRbohA and AtRbohF using same options. Another protein kinase OST1 was common for two Rbohs (AtRbohB and AtRbohF) with better confidence score (0.99) for AtRbohF than AtRbohB (0.408). Although its interaction with AtRbohF is known in stomatal closure [25], however no experimentally recorded interactions with AtRbohB are available so far. Another class of protein kinases consists of Ca^2+^-regulated CBL-interacting protein kinases (CIPKs) that are known to be activated upon interaction with Calcineurin B-like (CBL) Ca^2+^ sensor proteins. Several diverse functions have been documented for various CBL-CIPK pairs [26]. In the present study, only AtRbohG was predicted to interact with CIPK25. A recent report has suggested the role of CIPK25 in biotic stress and energy sensing [27, 28]. However, another study has reported the interaction of AtRbohF with CBL1/9-CIPK26 complex and its involvement in the regulation of ABA responses [29].

In addition to protein kinases, AtRbohs also showed interaction with different Rac proteins. Rac are known to display diverse array of functions in the plants [30]. In our study, the interactions between AtRbohA and AtRbohB with Rac6 and AtRboh C with Rop2, Rac3 and Rac5 were observed.

Among 10 AtRbohs, AtRbohC appeared to have unique functional partners, all of which are involved in root development. The role of AtRbohC in root hair formation is well known [31], however no experimental reports are available regarding its interaction partners. On the other hand, AtRbohH was observed to interact with only three partners (ATRDH2, AT2G35040 and GLP4). An earlier study has documented the role of ATRDH2 (also known as Rhodanese Homologue 2 or STR2; Sulfurtransferase 2) in embryo and seed development [32]. On the other hand, GLP4 (Germin-Like Protein 4) is implicated in the plant defense, auxin-induced cell growth and exhibiting superoxide dismutase activity [33–35]. Although, the role of AT2G35040 has not been elucidated, it may belong to AICARFT/IMPCHase bienzyme family as evident from our UniProt analysis. Two partners (AT2G35040 and GLP4) of AtRbohH are common with AtRbohE and AtRbohI, and one (AT2G35040) with AtRbohJ. Previous reports have indicated AtRbohH and AtRbohJ as pollen-specific [36], which may be related to one common partner (AT2G35040) among them, however, function of AtRbohE is still unknown. In addition to two common partners (AT2G35040 and GLP4) with AtRbohH, AtRbohE has one common partner (CYP707A4) with two other Rbohs (AtRbohG and AtRbohI). CYP707A4 is an abscisic acid 8′-hydroxylase 4 which is involved in ABA catabolism during drought conditions [37]. Further, AtRbohE appeared to interact with four unique partners (DGL, ATEXPA2, AT1G17680 and GA20OX3). Two (DGL and GA20OX3) are involved in biosynthesis of hormones, where DGL (DONGLE) possess galactolipase activity and involves in jasmonic acid biosynthesis [38] while GA20OX3 (Gibberellin 20 oxidase 3) in gibberellin acid biosynthesis [39]. ATEXPA2 (α-expansin 2) plays role in cell wall loosening and development processes [40], while AT1G17680 is still uncharacterized. Further, AtRbohG along with AtRbohI and AtRbohJ were also observed to interact with many unique partners.

In case of rice Rbohs, except OsRbohA which has five unique and five common partners, the observed frequency of common partners was higher among other eight Rbohs. The unique interaction partners for OsRbohA involves immutans (chloroplastic alternative oxidase), three superoxide dismutases (FeSOD1, FeSOD2 and MnSOD) and protein kinase WNK4. Immutans are known in ROS-related damage prevention under high light stress [41], FeSOD1 and FeSOD2 in drought stress; and MnSOD in drought stress and embryogenesis [42, 43]. However, the role of WNK4 is still unknown. Another protein, two pore calcium channel protein1 (TPC1) was observed to interact with OsRbohB only. The role of TPC1 in the regulation of growth and development is well documented [44]. Other partner (4339304) encoding Rac protein expressing under salt stress, was found to interact with two rice Rbohs (OsRbohA and OsRbohB) only [45]. It is interesting to note that one partner with gene ID: LOC_Os04g31290.1 (basic helix-loop-helix (bHLH) DNA-binding domain containing protein) was found to interact with all nine rice Rbohs and acting as a central hub. However, no information is available for its functional annotation yet. Functional characterization of such hub will lead to addition of knowledge in the area.

Overall, the present interaction study and gene ontology classifications have provided insights into the interaction of AtRbohs and OsRbohs with a wide range of potential partners which may be critical for their diverse functions. The observed high frequency of common and total partners in AtRbohs as compared to OsRbohs may indicate more complex interactions in AtRbohs. Further, pie distribution indicated that the majority of AtRbohs play role in stress-related activities, followed by cellular development. Similar kind of trend was also obtained for OsRbohs. These observations justifies the versatility of functions played by Rbohs as evident from literature [1].

### Analysis of phosphorylation sites for Arabidopsis and rice Rbohs

Protein phosphorylation and dephosphorylation are among the most crucial post-translational modifications, which play important role in a broad range of regulatory signaling cascades in plants. The phosphorylation of specific sites in proteins may result in conformational changes in protein structure which may lead to changes in enzyme activity, substrate specificity, biological role, intracellular localization, protein stability etc. Serine, threonine and tyrosine residues are the important amino acids which can be phosphorylated. Few studies in the past have provided hints for the regulation of Rbohs through phosphorylation via Ca^2+^ and different types of protein kinases such as calcium-dependent protein kinase (CDPK), Ca^2+^ /CaM-dependent protein kinase (CCaMK), mitogen activated protein kinase (MAPK), BIK1 (receptor-like cytoplasmic kinase) etc. [1, 9, 11, 12]. However, this area need to be fully investigated, though it is very time-consuming and expensive to identify a broad range of phosphorylation sites experimentally. Hence, in silico prediction of phosphorylation sites provides an alternative approach [46]. In the present study, potential serine (S), threonine (T) and tyrosine (Y) phosphorylation sites were predicted. Their distribution and location were studied by mapping them on the multiple sequence alignment of 19 Rbohs. It was interesting to note that the experimentally verified sites for few AtRbohs were also obtained as potential phosphorylation sites in our prediction outputs and hence, provided hints for conservation and variability within other AtRbohs. Earlier evidences have indicated the diverse roles of AtRbohD and AtRbohF in plants involving growth and development, abiotic and biotic stresses [1]. AtRbohD was found to be phosphorylated at S-8, S-39, S-148, S-152, S-163, S-343 and S-347 in response to pathogen elicitors [24, 47, 48]. In the present study, the conservation of equivalent serine residues corresponding to S-148 in four and S-163 in all AtRbohs, suggest N-terminal phosphorylation-mediated regulation in them. Further, S-133 and S-148 from AtRbohD corresponds to S-82 and S-97 from *S. tuberosum* StRbohB, which have been identified as potential phosphorylation sites for StCDPK4 and StCDPK5 [49]. An earlier study showed that S-13 and S-174 from AtRbohF are phosphorylated by OST1 protein kinase, where OST1 is known to phosphorylate S/T from the motif [LIMVF]XRXXS/T [25, 50]. In the present work, the conservation of S-174 among all AtRbohs as well as that of arginine (R) at the − 3 position relative to S-174 may indicate that they can be phosphorylated by OST1 kinase. S-174 from AtRbohF corresponds to that of S-163 from AtRbohD. Some line of evidences have reported the functional redundancy among AtRbohD and AtRbohF, however, AtRbohD is mostly responsible for ROS in plant-pathogen interactions while AtRbohF in ABA signaling [51, 52]. This might be due to variation among few phosphorylation sites, which lead to differential regulation and function. Besides AtRbohD and AtRbohF, the function of AtRbohC has also been elucidated which is involved in root-hair development [31]. An earlier study has identified S-318 and S-322 as possible sites of phosphorylation in AtRbohC [53]. In the present study, the conservation of equivalent serine residues corresponding to S-318 in six and S-322 in three AtRbohs, may suggest their potential role in root development. The experimental information regarding phosphorylation for other AtRbohs and OsRbohs is still lacking. However, our mapping of the predicted potential phosphorylation sites among all 19 Rbohs indicates their abundance in the N-terminal region with higher phosphorylation of serine residues as compared to threonine and tyrosine. Further, we computed the distribution patterns of all S, T and Y residues in different regions of 19 Rbohs. It was observed that the frequency of occurrence of these residues is highest in the N-terminal upstream EF-hand region from both AtRbohs and OsRbohs. To know whether this concept holds good for other EF-hand containing proteins, genome-wide analysis of EF-hand containing proteins from Arabidopsis and rice was carried out. The distribution pattern of S, T and Y were analyzed for few representative proteins from Arabidopsis and rice. Interestingly, we obtained similar kind of pattern for the selected proteins, which verified our results and hence may point towards the critical role of the N-terminal upstream EF-hand region in contributing functional multiplicity to AtRbohs and OsRbohs. In addition to S, T and Y, other important residues including lysine (K), arginine (R), proline (P) and cysteine (C) were also studied. These residues are known to play crucial roles in transmembrane proteins such as lysine and arginine provide assistance in anchoring the transmembrane orientations, proline in stress tolerance and cysteine in membrane localization [54–57]. Like S, T and Y, the frequency of occurrence of these residues (K, R, P and C) was found highest in the N-terminal upstream EF-hand region for AtRbohs, OsRbohs and representative proteins.

The current comprehensive in silico study provides a necessary clue that may be the N-terminal amino acid residues from Rbohs and their phosphorylation are very critical for regulating various biological functions in a plant. A recent study on Rbohs sequence and structural analysis has also provided hints towards the role of the N-terminal and its variability for their functional diversity [6]. It will be further interesting to test experimentally the interaction with the potential partners, the interaction sites, any overlapping regions, the role of correct intracellular location of the partner, the phosphorylation ability and preferences of the predicted residues among various Rbohs.

## Conclusion

In the present work, in silico approaches were followed to comprehensively deduce the possible interacting partners and phosphorylation sites of Rboh gene family from two model plants (Arabidopsis and rice). The study elucidates an extensive range of potential partners revealed from interaction networks and gene ontology classifications, which may be responsible for their functional multiplicity. Further insights were also obtained from the prediction of potential phosphorylation sites as well as their distribution, location and kinase specificities. These results were correlated with experimental data as well as verified with the other EF-hand containing proteins. However, more and more inputs from the experimental work will further strengthen our assumptions and pave the way to modulate plant species to address the future challenges, for instance, crops with better stress adaptibility.

## Additional files

Additional file 1: Table showing Arabidopsis and rice Rboh protein sequences retrieved from UniProt. (PDF 13 kb)

Additional file 2: Individual network diagram of few AtRboh proteins showing potential interacting partners in evidence view using no more than 20 interactors option. Different coloured lines indicate types of evidence for association. The thickness of each line indicates the strength of the association. (PDF 549 kb)

Additional file 3: Individual network diagram of few AtRboh proteins showing potential interacting partners in evidence view using no more than 50 interactors option. Different coloured lines indicate types of evidence for association. The thickness of each line indicates the strength of the association. (PDF 1926 kb)

Additional file 4: Table showing functional categorization of identified interaction partners among AtRbohs. (PDF 303 kb)

Additional file 5: Combined network diagram of 9 OsRboh proteins showing potential interacting partners in evidence view with (a) 10 interactors (b) 20 interactors and (c) 50 interactors. Different coloured lines indicate types of evidence for association. The thickness of each line indicates the strength of the association. (PDF 811 kb)

Additional file 6: Individual network diagrams of 9 OsRboh proteins showing potential interacting partners in evidence view with 10 interactors. Different coloured lines indicate types of evidence for association. The thickness of each line indicates the strength of the association. (PDF 1978 kb)

Additional file 7: Individual network diagram of OsRboh proteins showing potential interacting partners in evidence view using no more than 20 interactors option. Different coloured lines indicate types of evidence for association. The thickness of each line indicates the strength of the association. (PDF 855 kb)

Additional file 8: Individual network diagram of OsRboh proteins showing potential interacting partners in evidence view using no more than 50 interactors option. Different coloured lines indicate types of evidence for association. The thickness of each line indicates the strength of the association. (PDF 491 kb)

Additional file 9: Table showing details of identified potential functional partners of OsRboh proteins. (PDF 259 kb)

Additional file 10: Table showing various unique and common functional partners among OsRbohs in color coding, where unique partners are uncoloured. (PDF 101 kb)

Additional file 11: Table showing functional categorization of identified interaction partners among OsRbohs. (PDF 196 kb)

Additional file 12: Mapping of phosphorylation sites on multiple sequence alignment of AtRboh and OsRboh protein sequences. 19 Rboh sequences were aligned with Clustal Omega (http://www.ebi.ac.uk/Tools/msa/clustalo). Gaps were indicated with dashes in the sequences. CDPK-binding motifs, two EF-hand-likes, two EF- hands, six ransmembrane spanning domains (TMD I-VI), two FAD-binding and four NADPH-binding sites were shown in black boxes. Reported (also retrieved in prediction) S/T/Y sites involved in phosphorylation were highlighted in 654 green while predicted sites were indicated in cyan. (PDF 62 kb)
