# Supplementary material for: In silico insights on diverse interacting partners and phosphorylation sites of respiratory burst oxidase homolog (Rbohs) gene families from Arabidopsis and rice
Source: BMC Plant Biol. 2018 Aug 10;18:161. doi: 10.1186/s12870-018-1378-2 (PMC6086027; doi:10.1186/s12870-018-1378-2)
Supplement: Supplementary file 1 — Table showing Arabidopsis and rice Rboh protein sequences retrieved from UniProt. (PDF 13 kb) [file 12870_2018_1378_MOESM1_ESM.pdf]

Table. Rboh proteins from Arabidopsis and rice.

Species	Protein name	UniProt Accession No.
Arabidopsis thaliana	AtRbohA	O81209
	AtRbohB	Q9SBI0
	AtRbohC	O81210
	AtRbohD	Q9FIJ0
	AtRbohE	O81211
	AtRbohF	O48538
	AtRbohG	Q9SW17
	AtRbohH	Q9FJD6
	AtRbohI	Q9SUT8
	AtRbohJ	Q9LZU9
Oryza sativa	OsRbohA	Q0JJJ9
	OsRbohB	Q5ZAJ0
	OsRbohC	Q65XC8
	OsRbohD	Q0DHH6
	OsRbohE	Q8S1T0
	OsRbohF	Q0J595
	OsRbohG	Q69LJ7
	OsRbohH	Q2QP56
	OsRbohI	Q2R351
