# Supplementary figures and images for: In silico insights on diverse interacting partners and phosphorylation sites of respiratory burst oxidase homolog (Rbohs) gene families from Arabidopsis and rice
Source: BMC Plant Biol. 2018 Aug 10;18:161. doi: 10.1186/s12870-018-1378-2 (PMC6086027; doi:10.1186/s12870-018-1378-2)

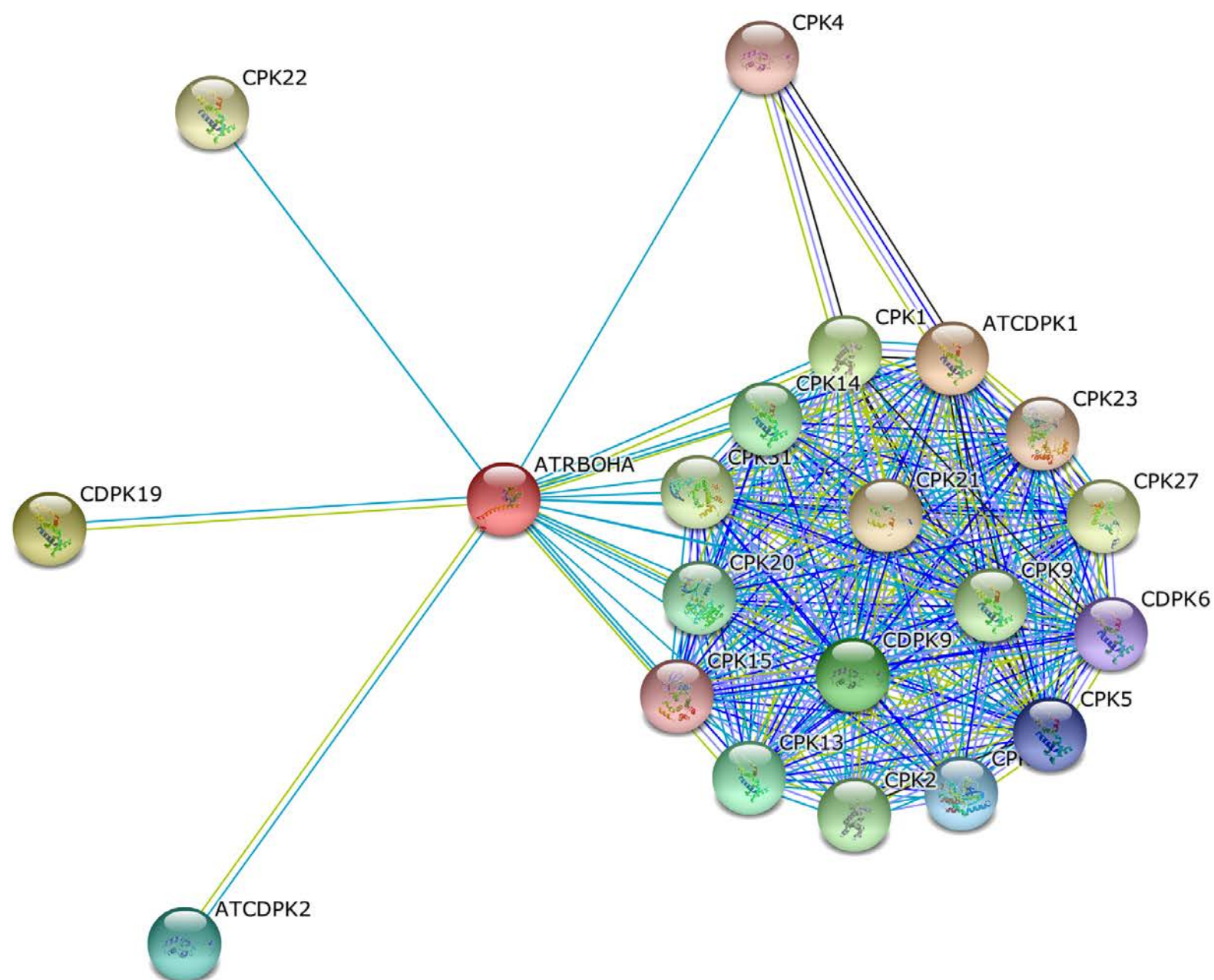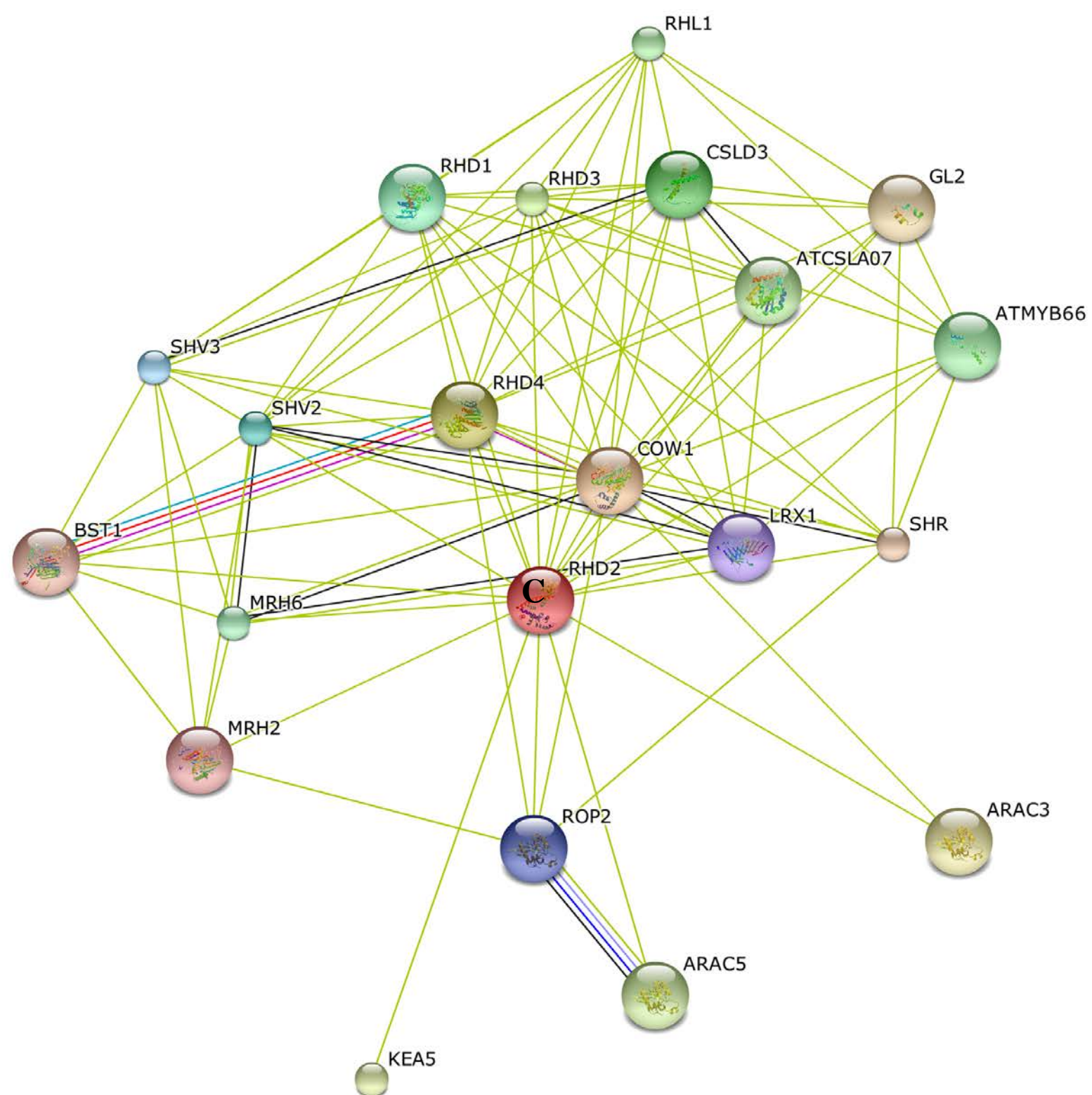

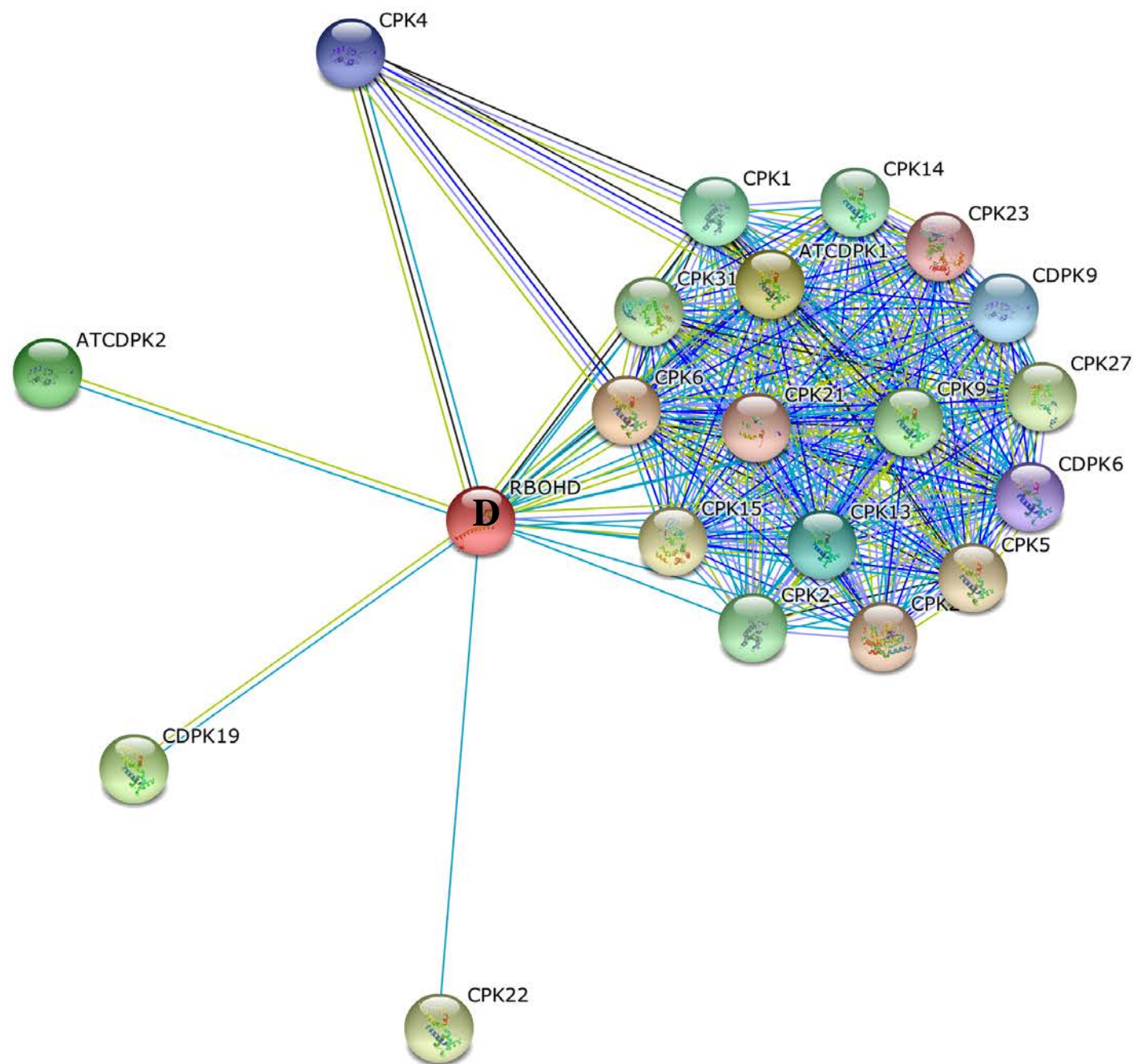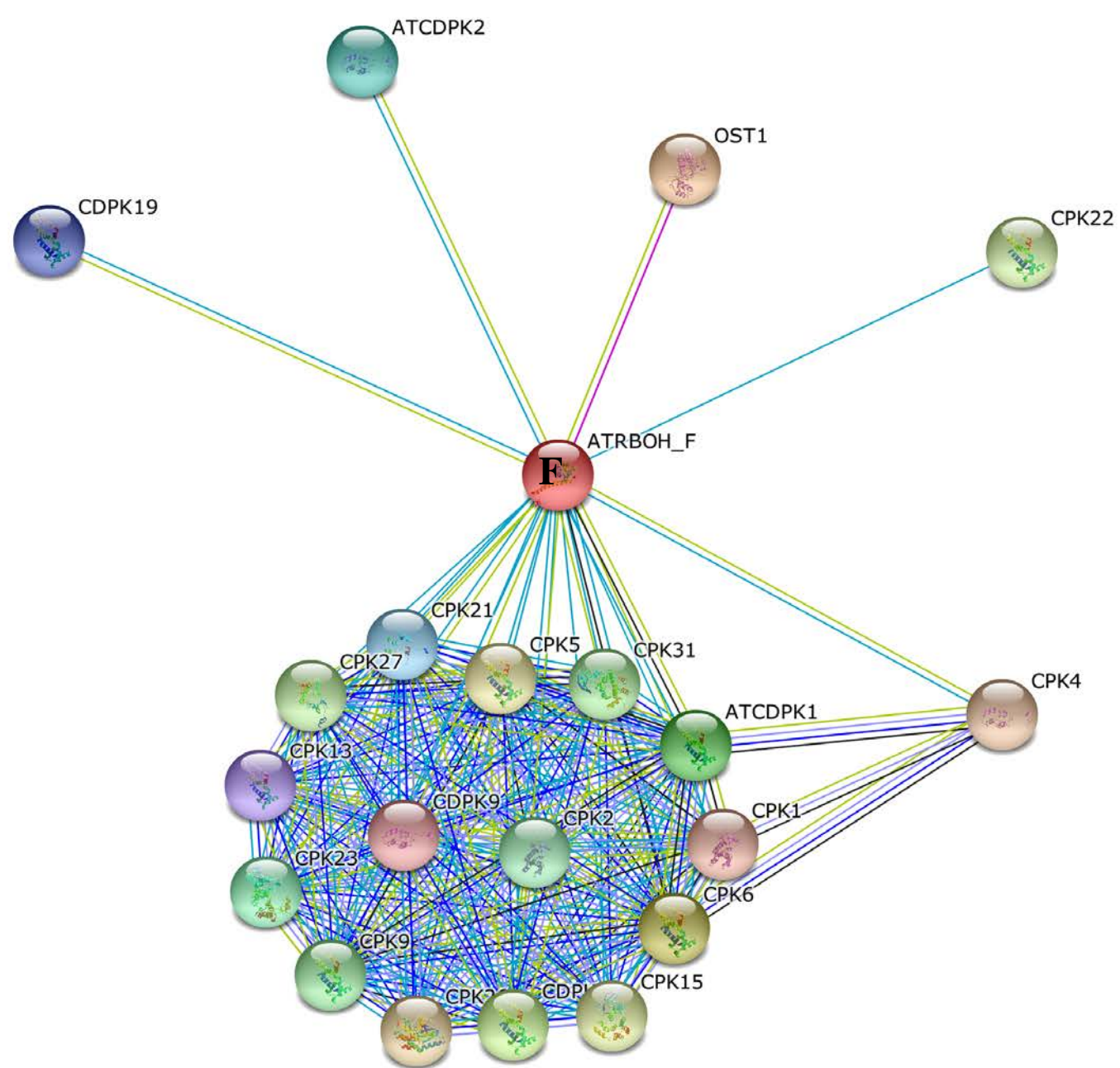

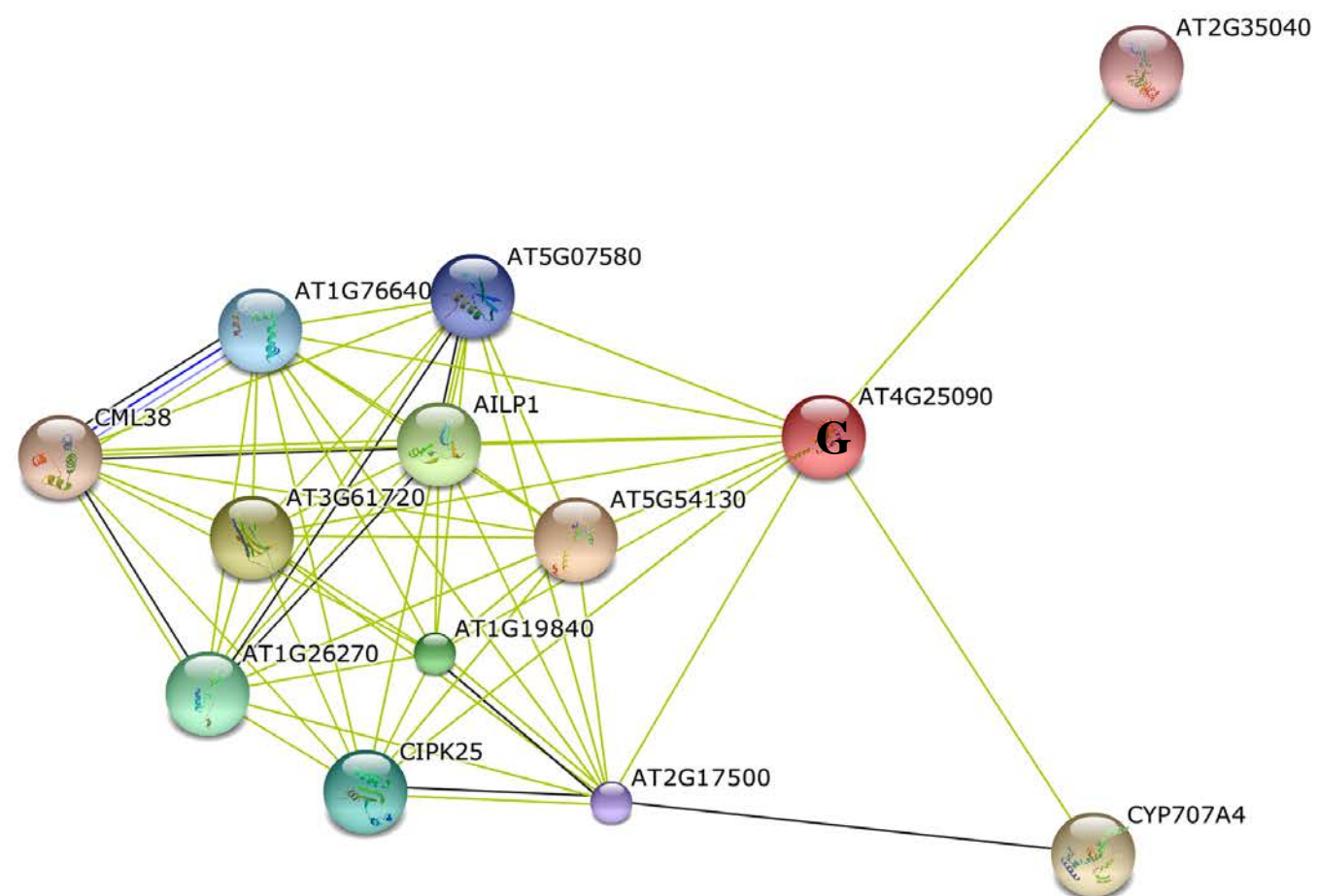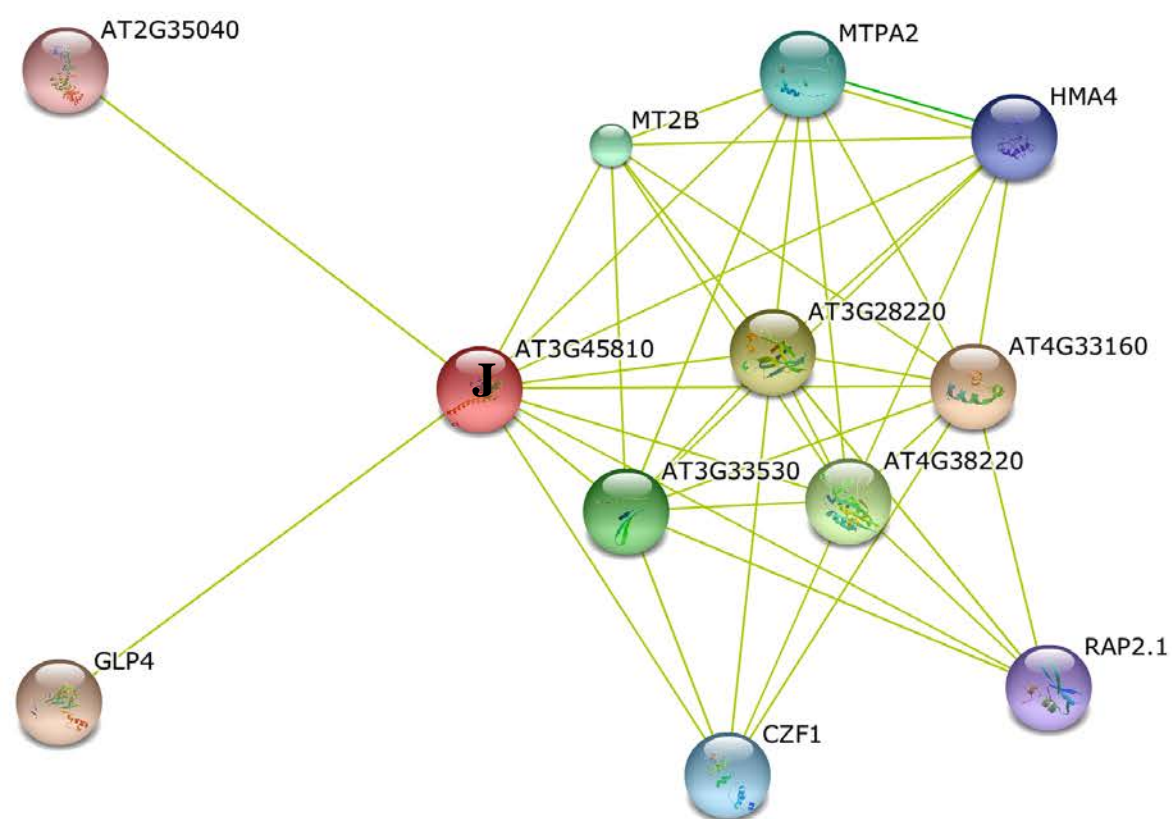

Supplement: Supplementary file 2 — Individual network diagram of few AtRboh proteins showing potential interacting partners in evidence view using no more than 20 interactors option. Different coloured lines indicate types of evidence for association. The thickness of each line indicates the strength of the association. (PDF 549 kb) [file 12870_2018_1378_MOESM2_ESM.pdf]

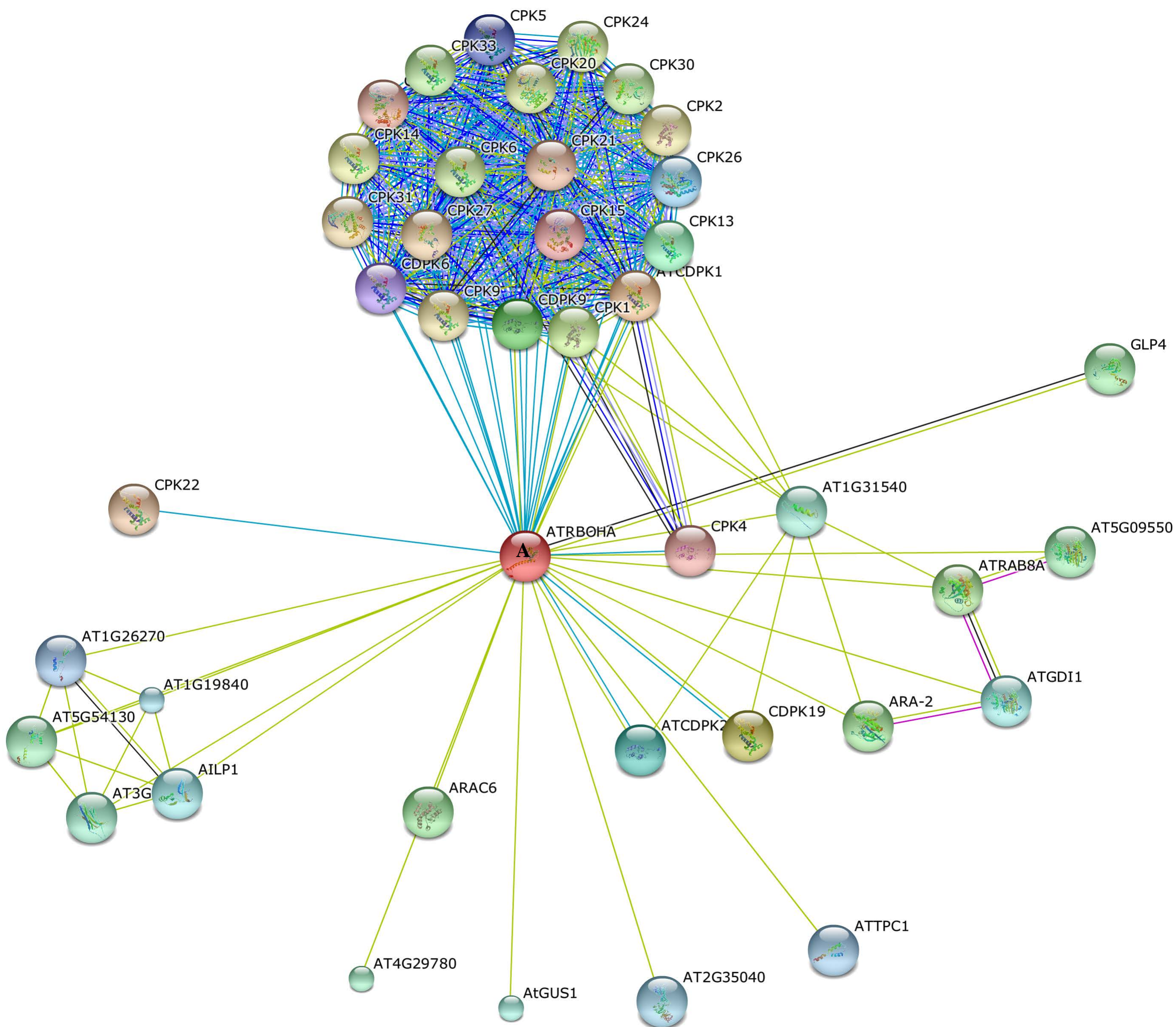

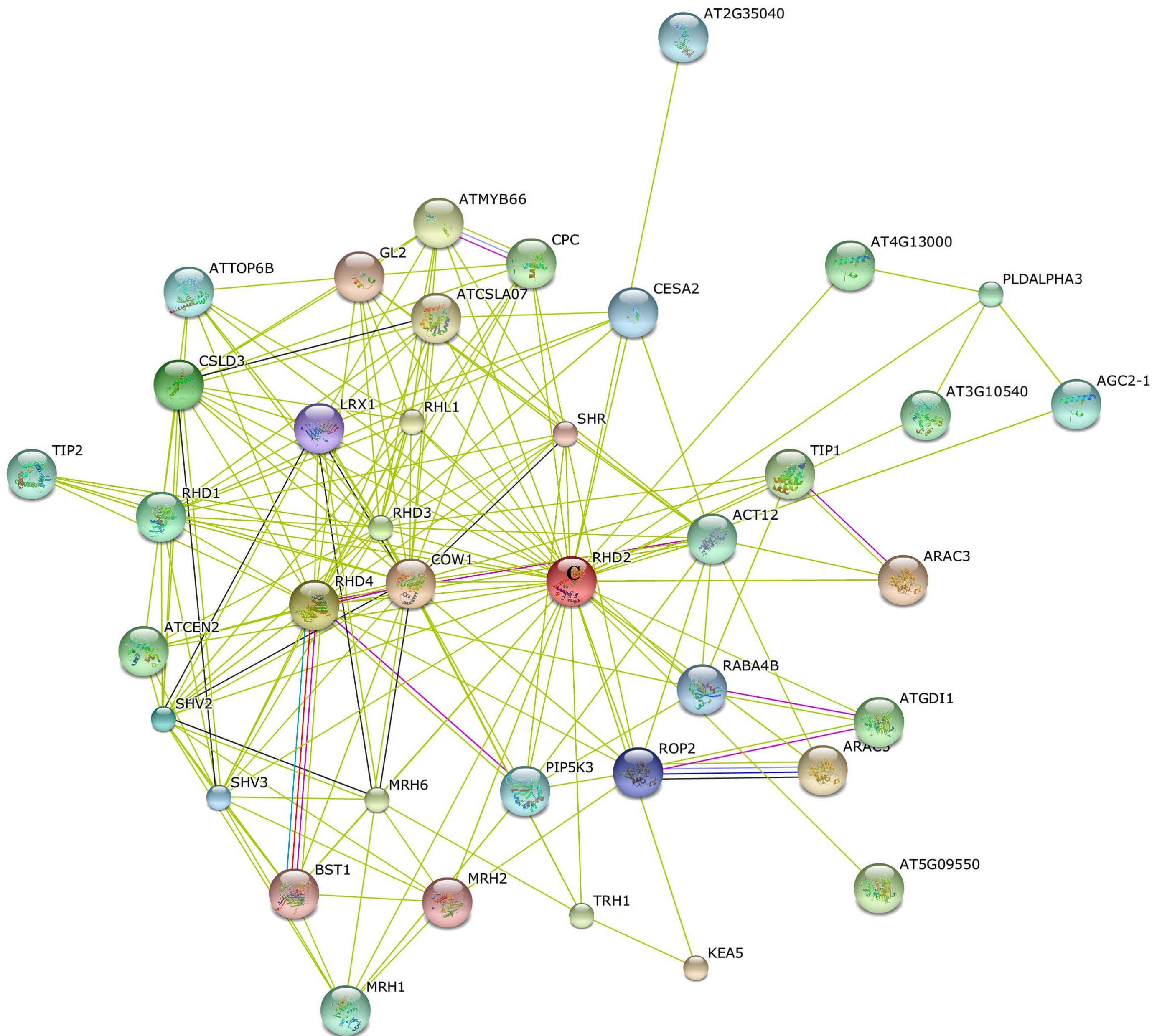

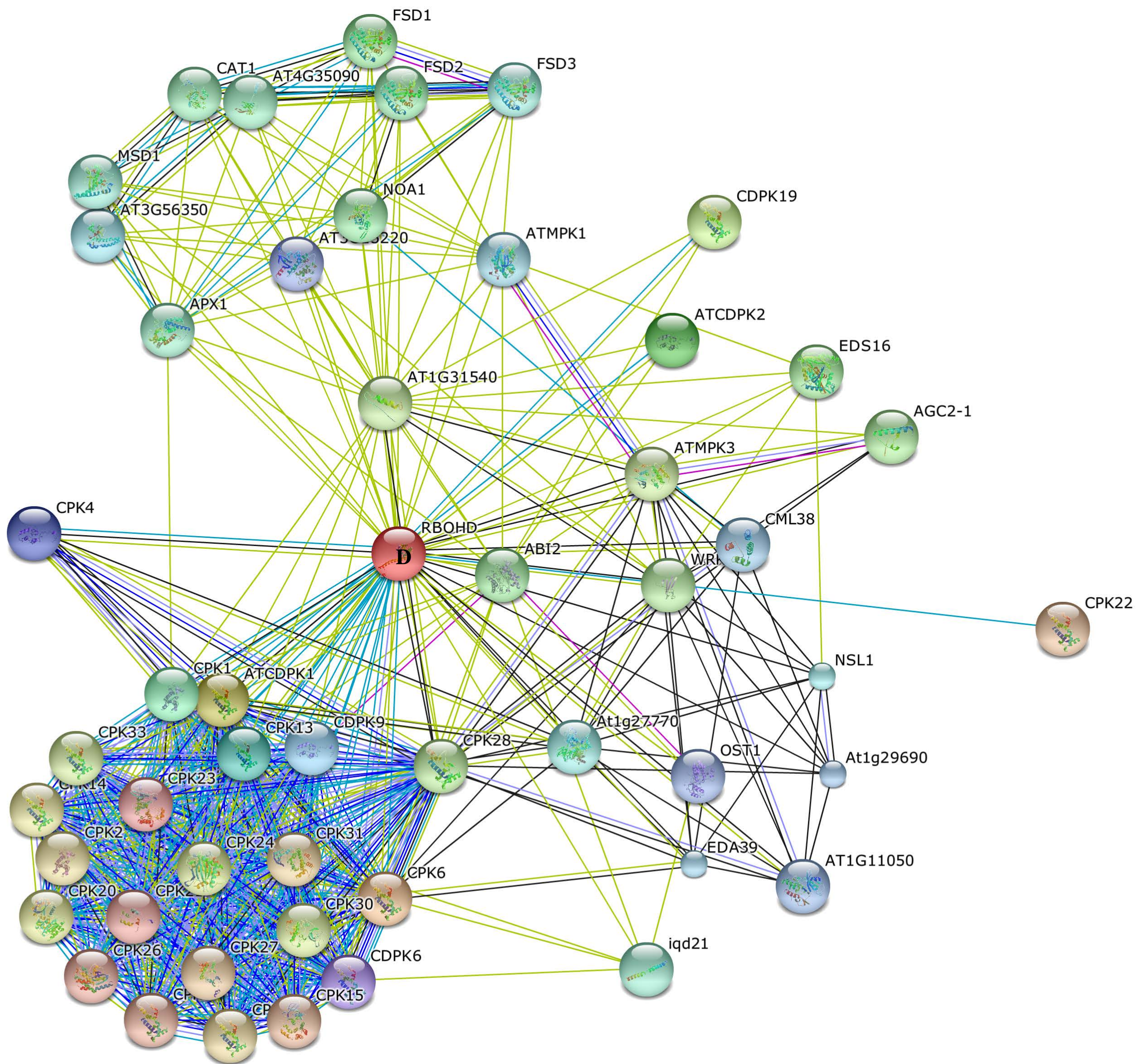

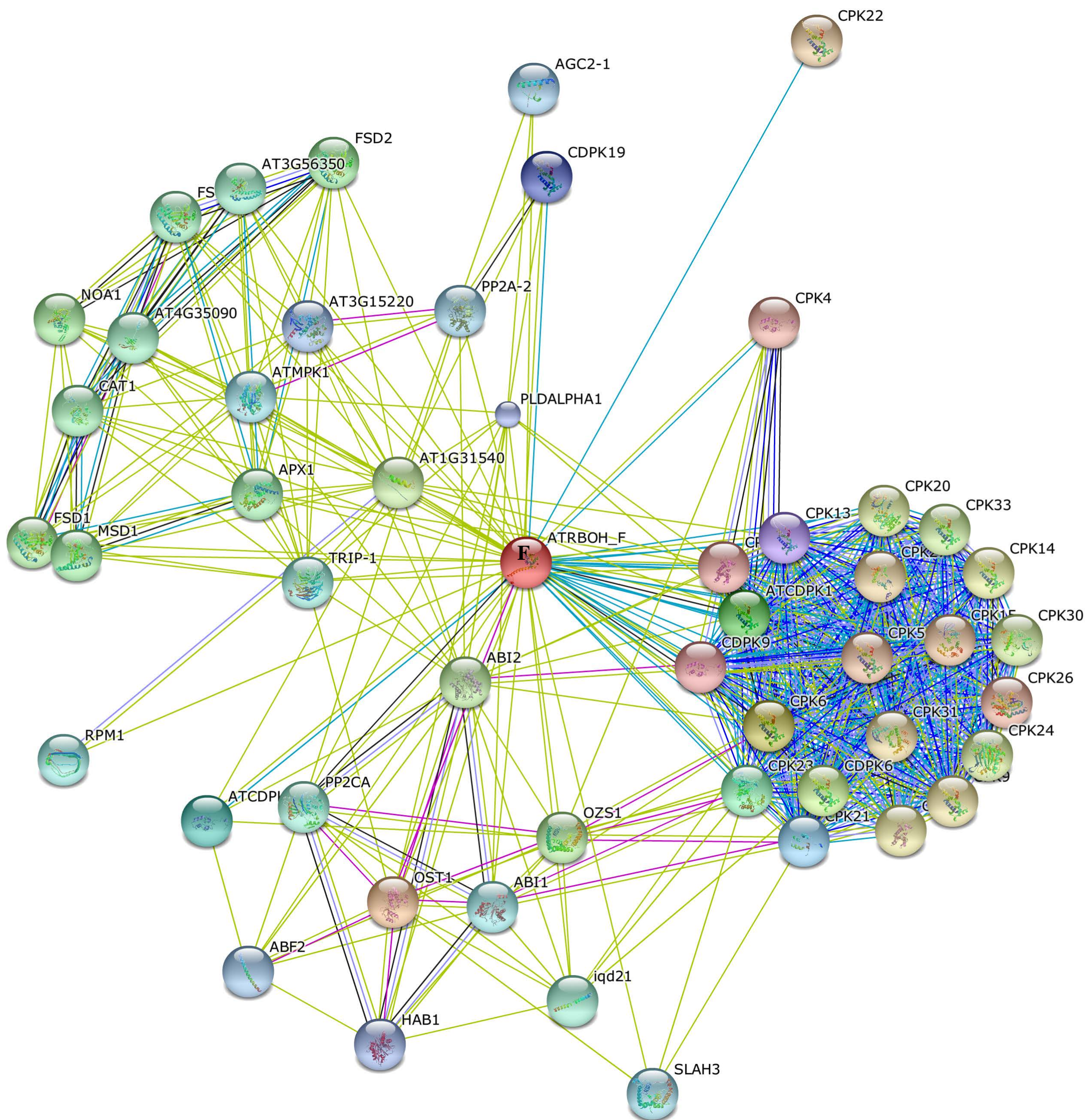

Supplement: Supplementary file 3 — Individual network diagram of few AtRboh proteins showing potential interacting partners in evidence view using no more than 50 interactors option. Different coloured lines indicate types of evidence for association. The thickness of each line indicates the strength of the association. (PDF 1926 kb) [file 12870_2018_1378_MOESM3_ESM.pdf]

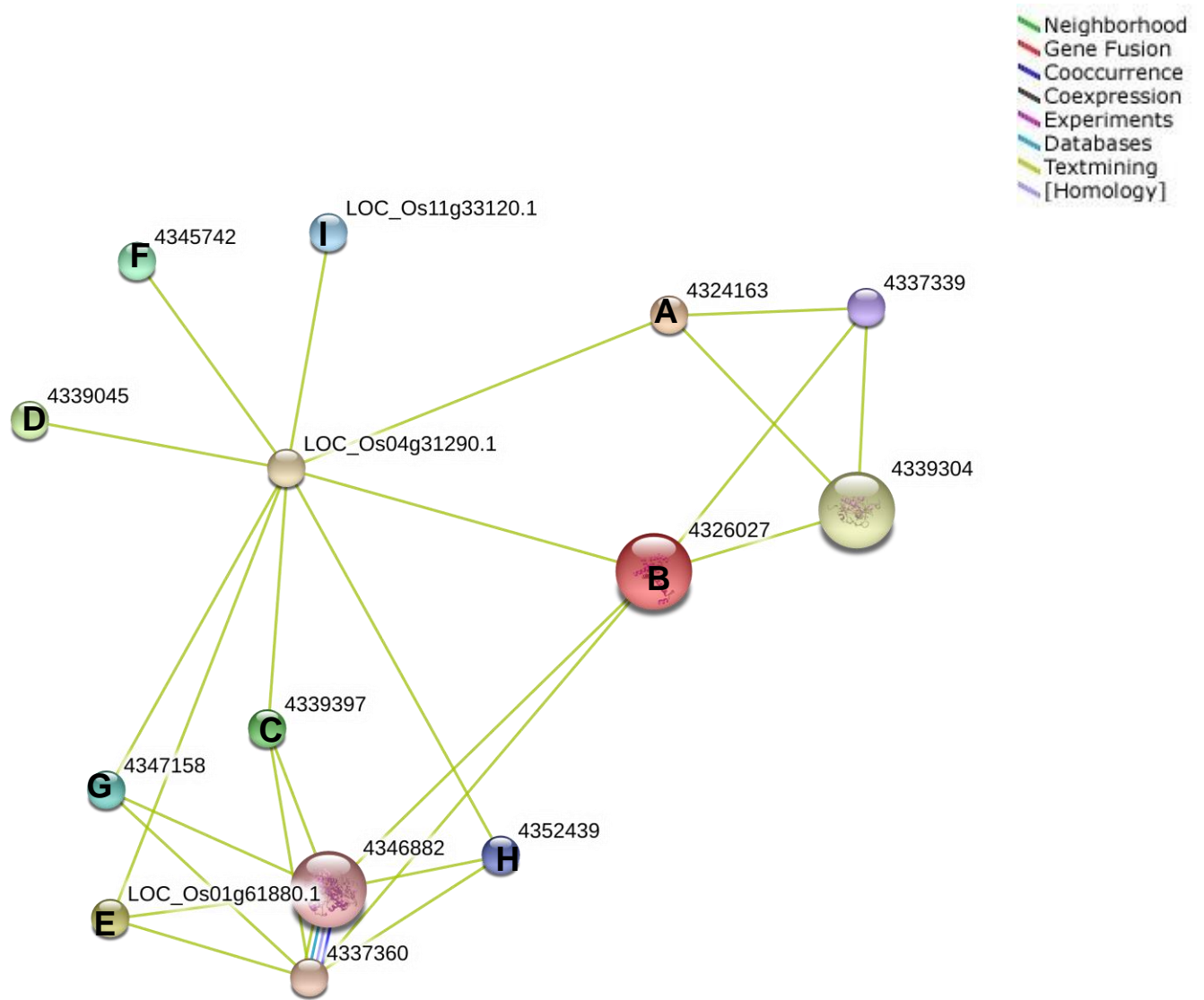

(a)

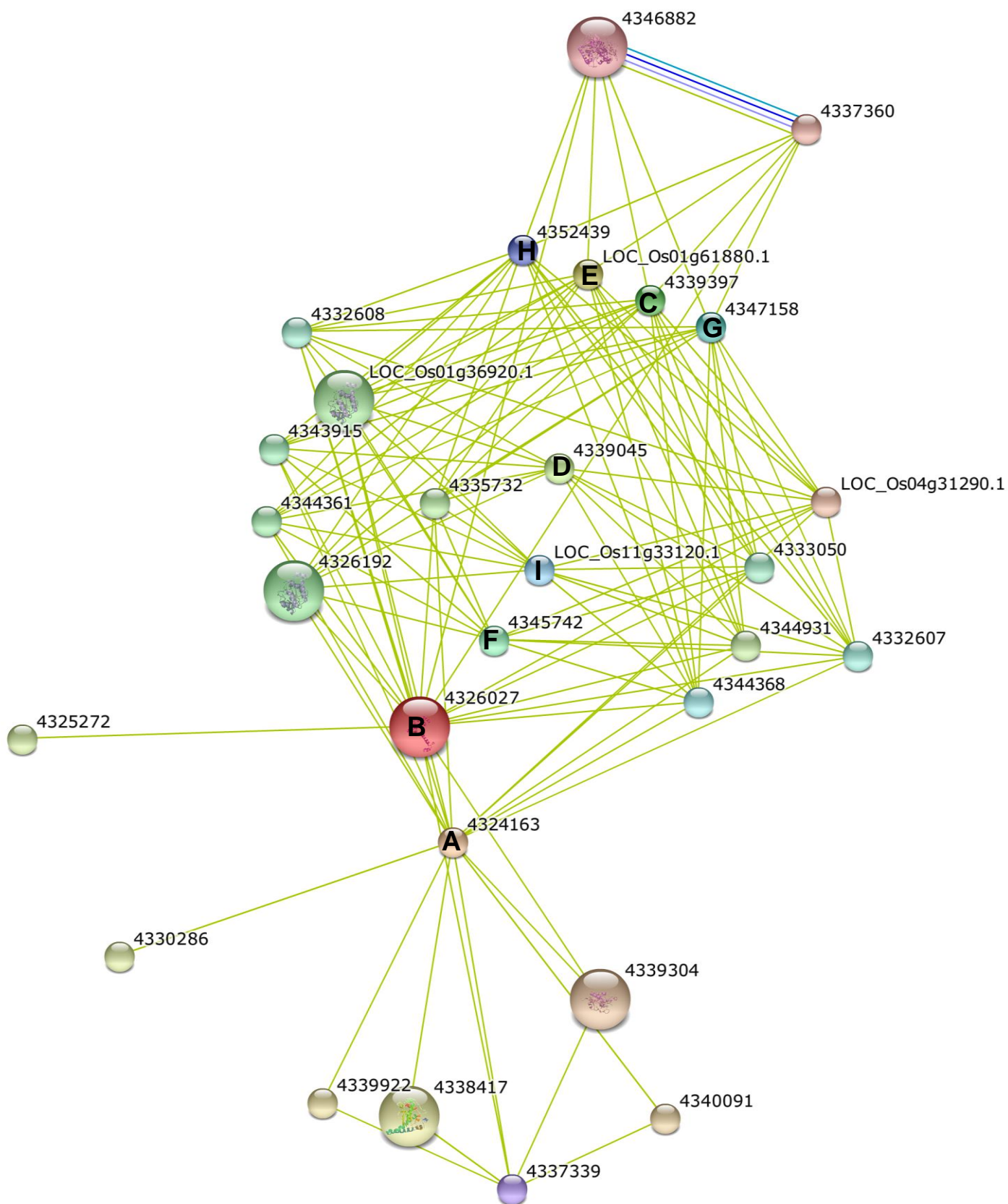

(b)

Supplement: Supplementary file 5 — Combined network diagram of 9 OsRboh proteins showing potential interacting partners in evidence view with (a) 10 interactors (b) 20 interactors and (c) 50 interactors. Different coloured lines indicate types of evidence for association. The thickness of each line indicates the strength of the association. (PDF 811 kb) [file 12870_2018_1378_MOESM5_ESM.pdf]

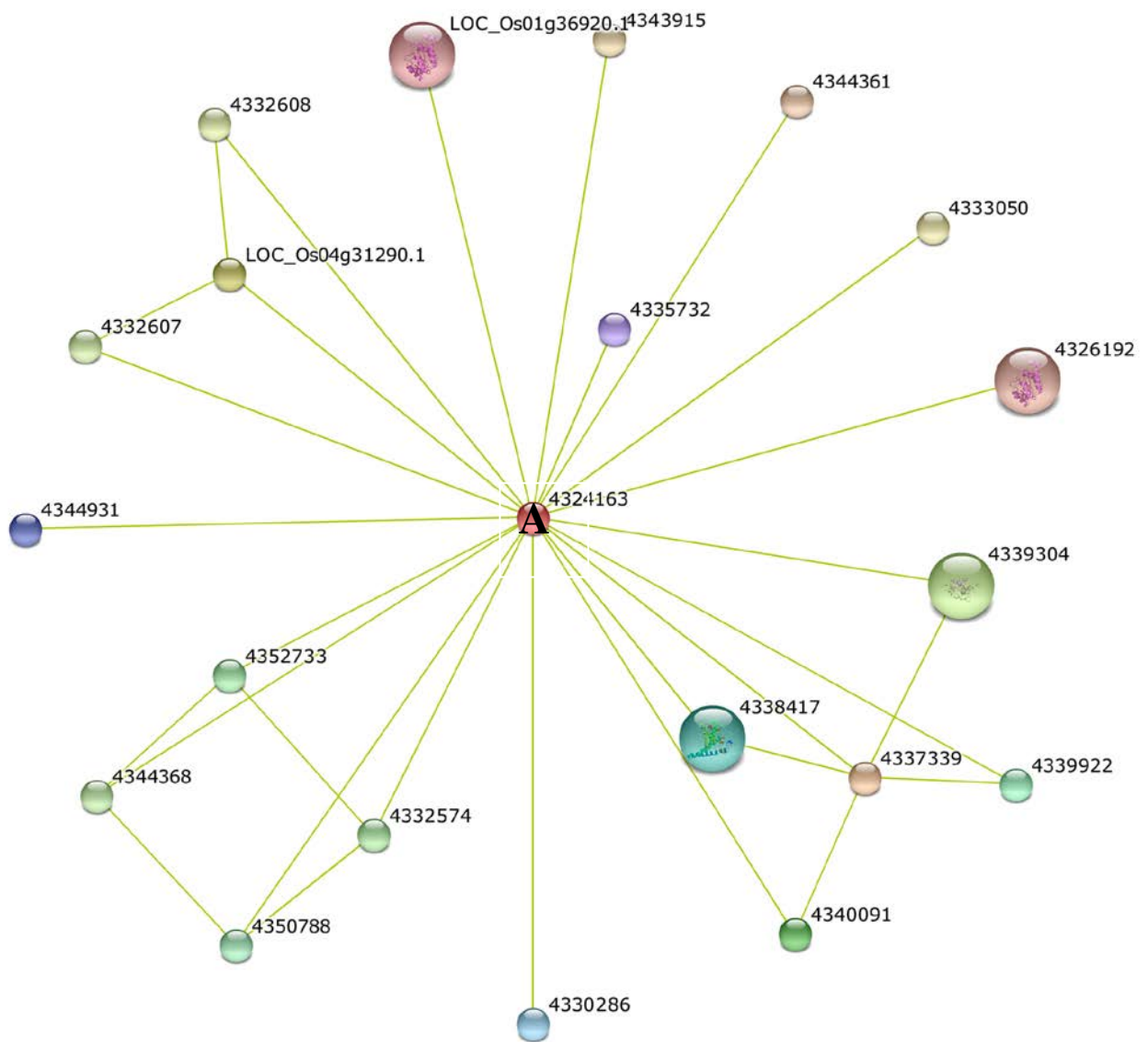

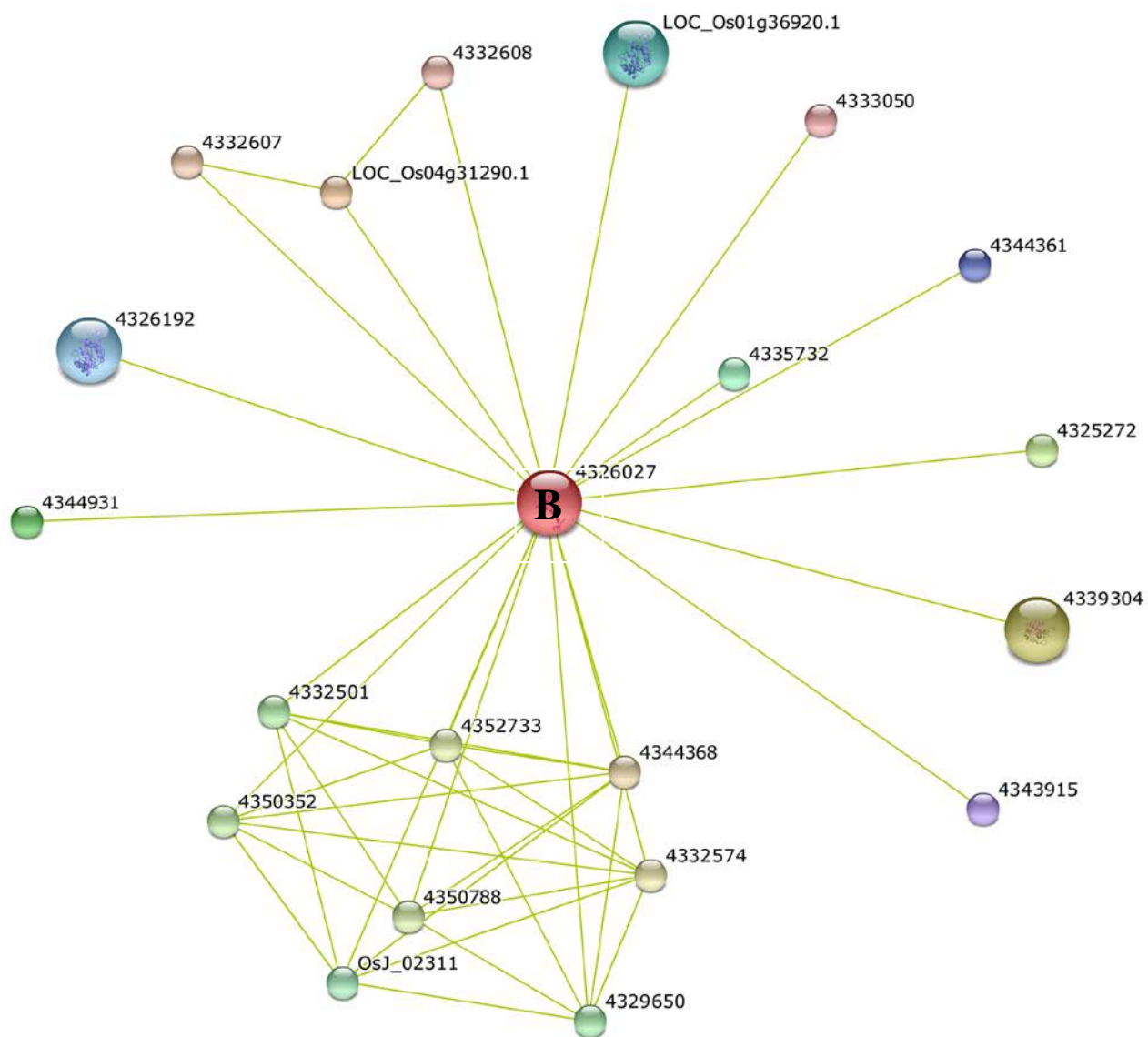

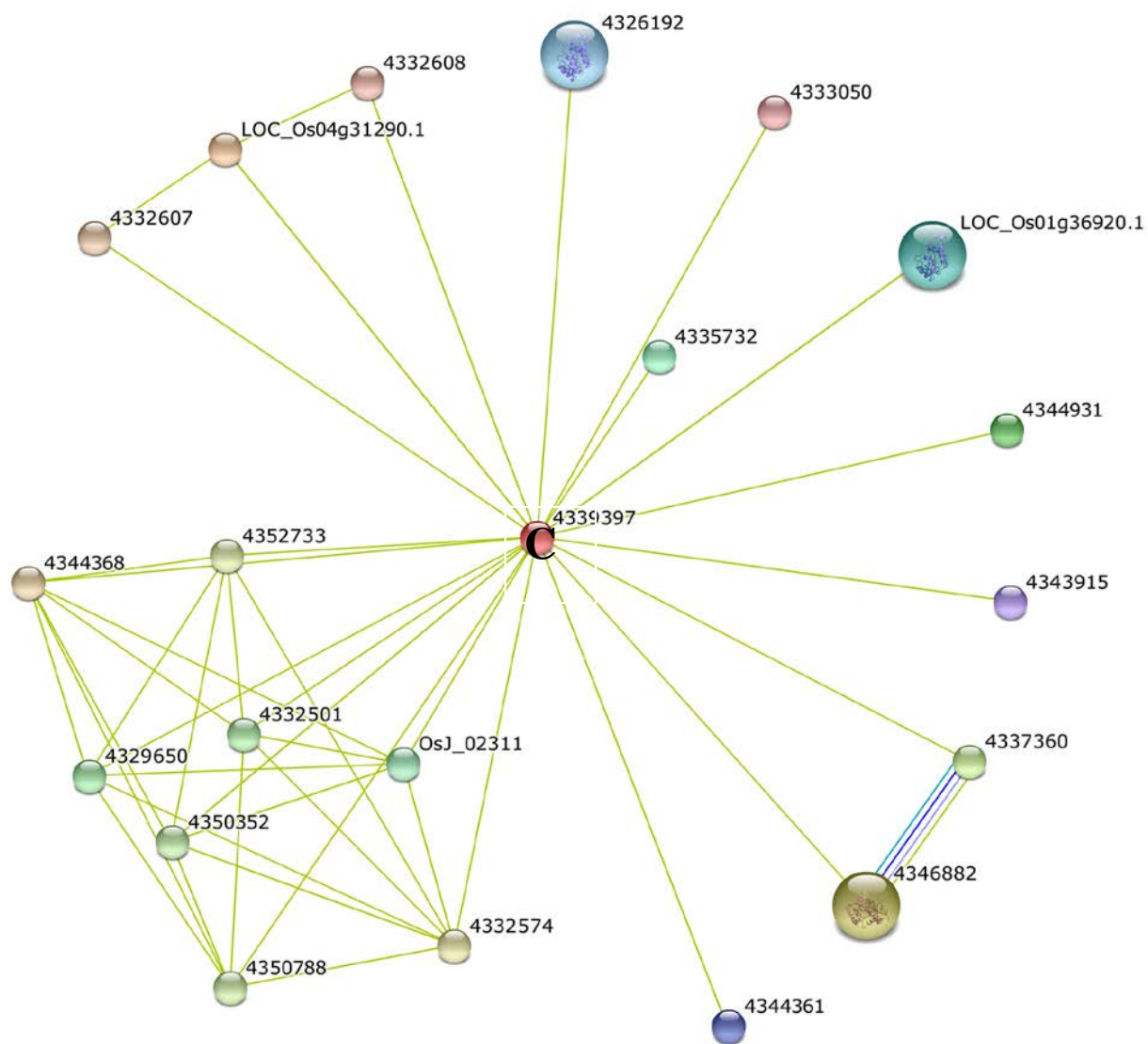

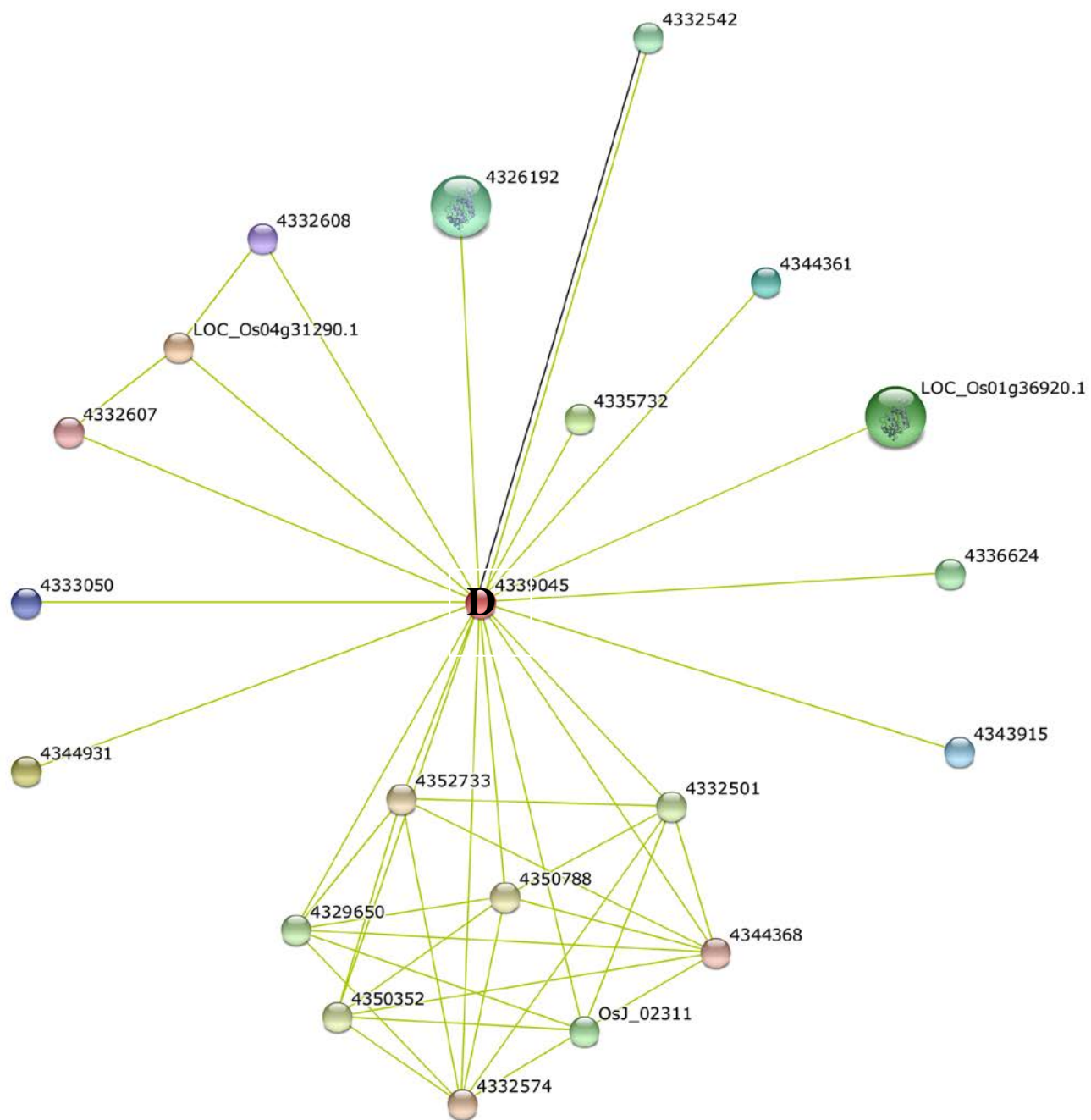

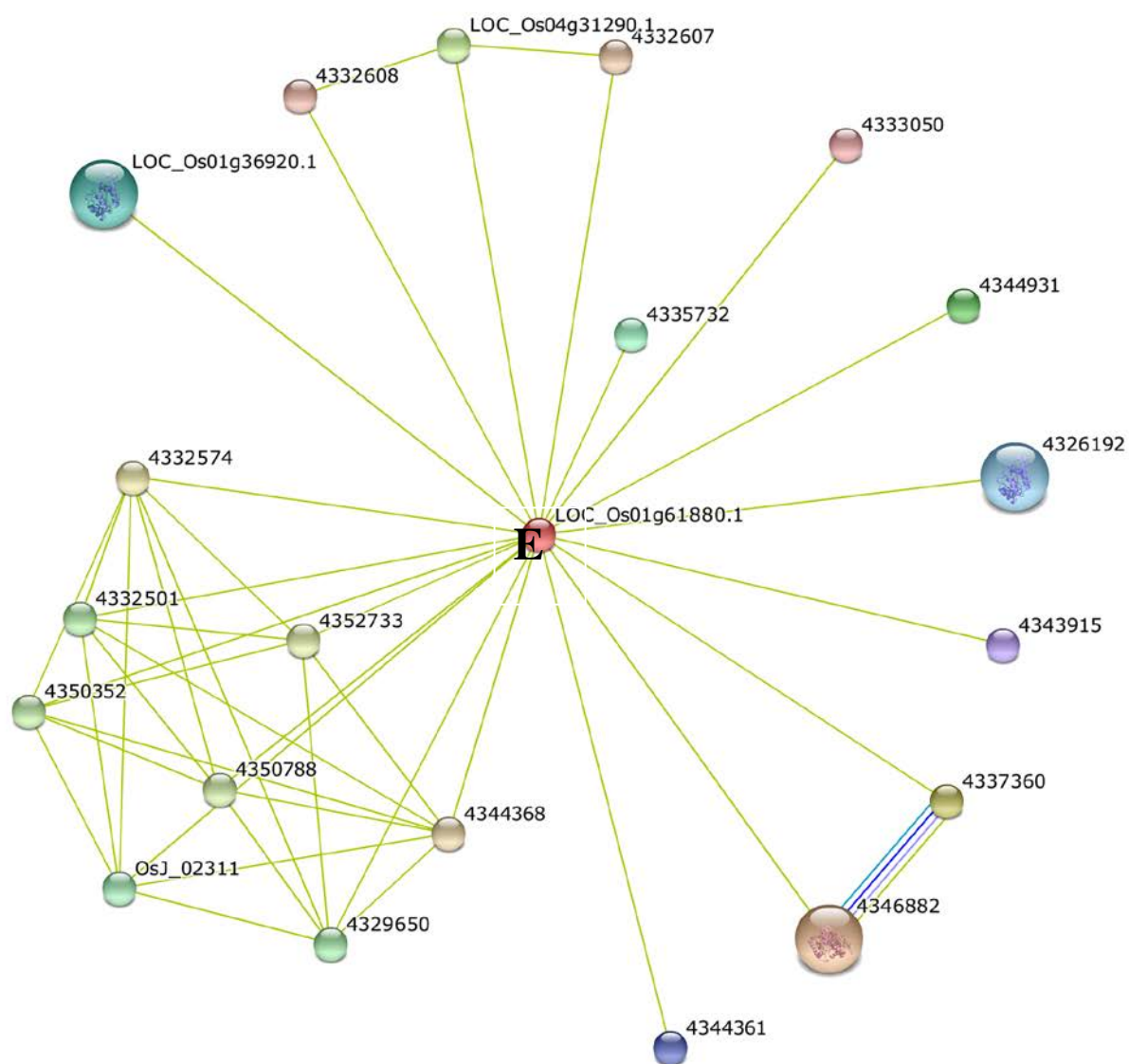

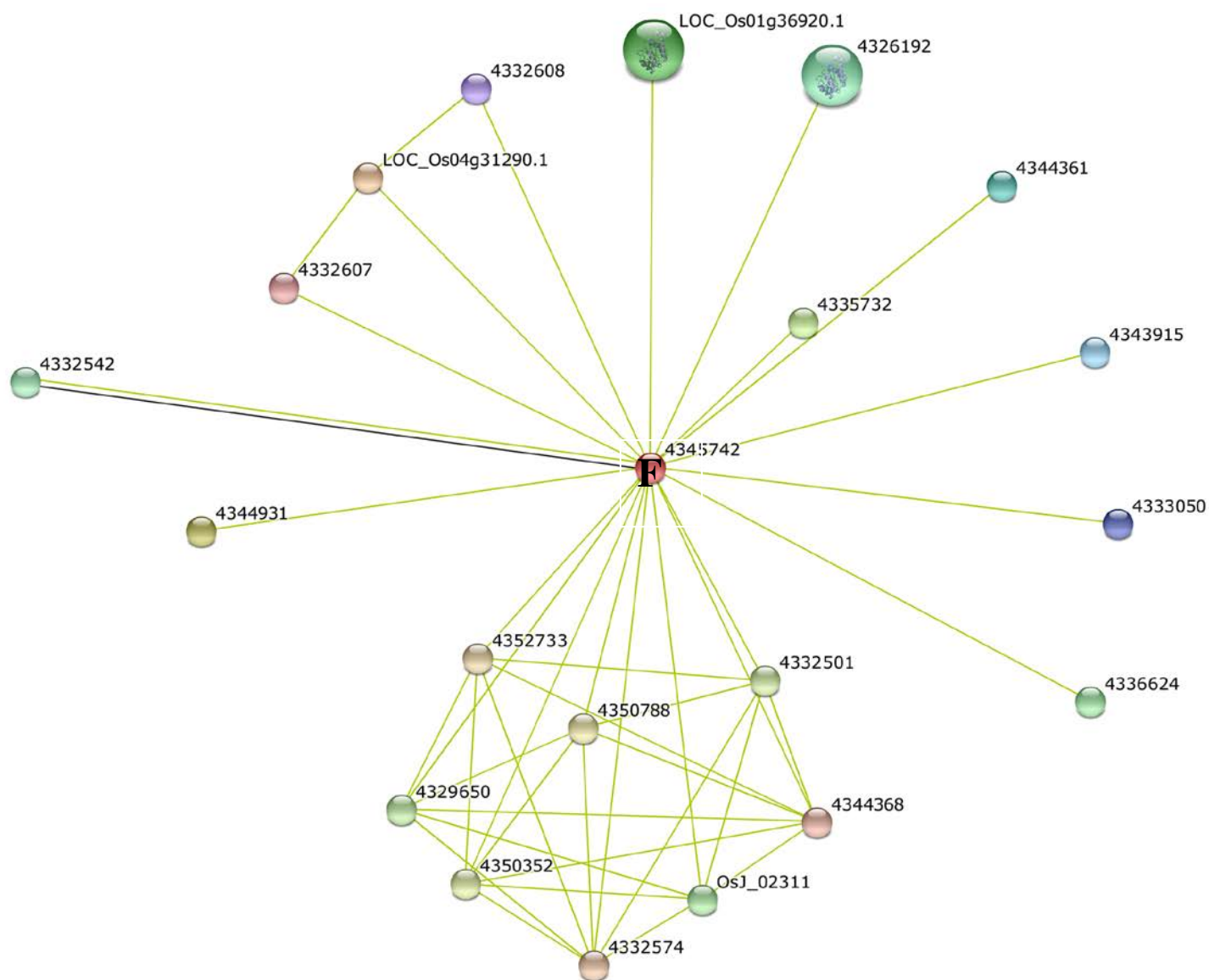

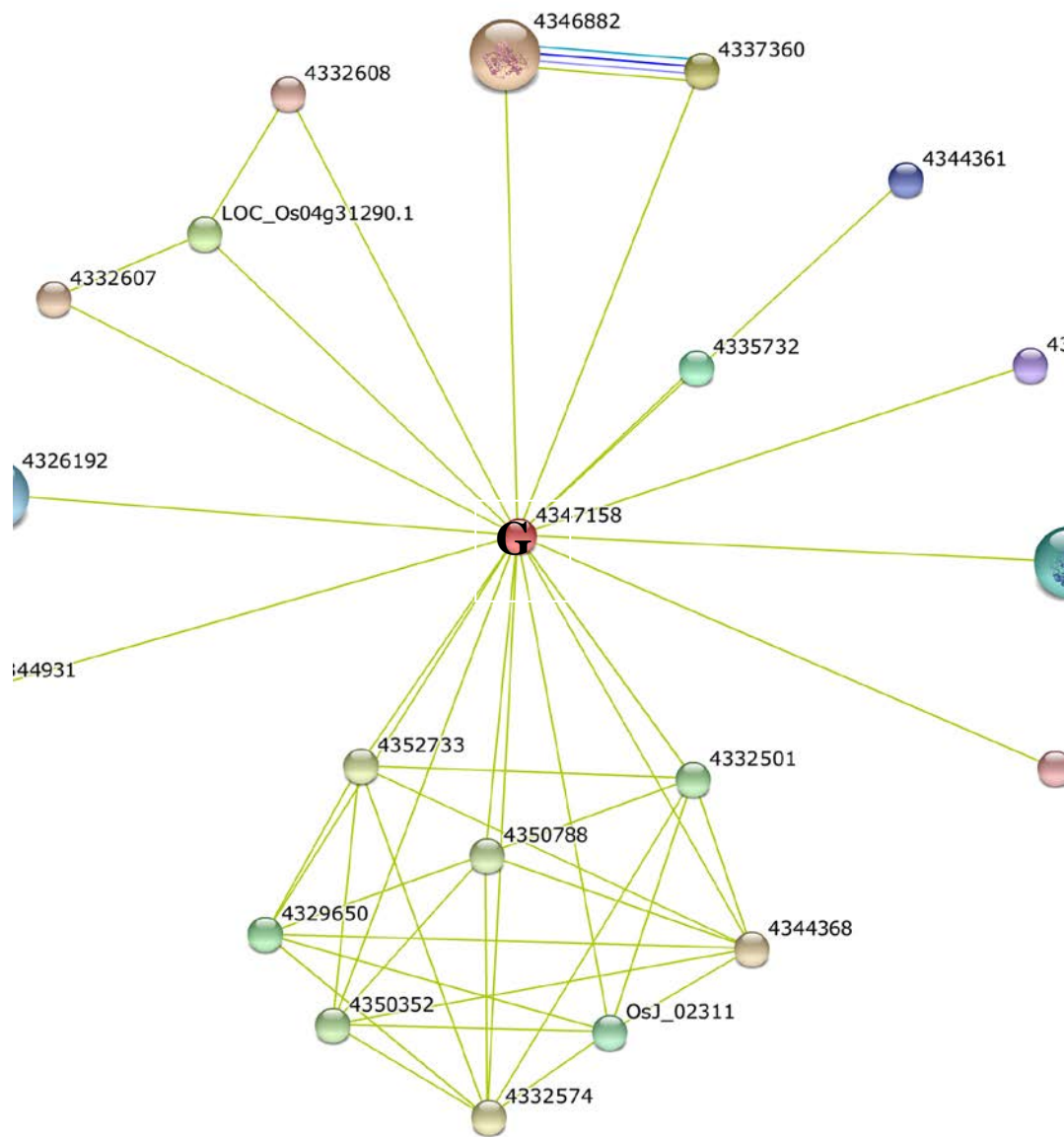

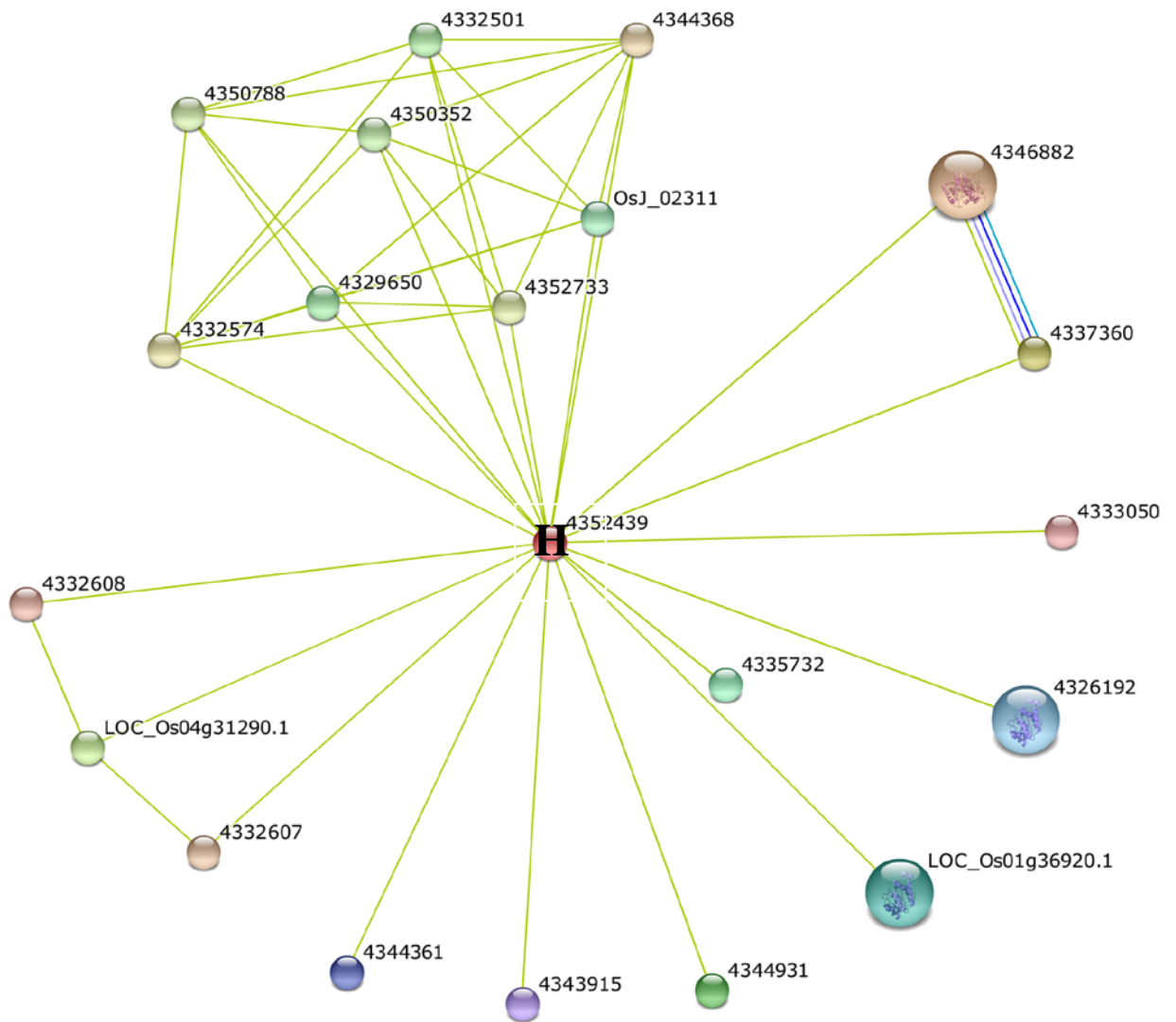

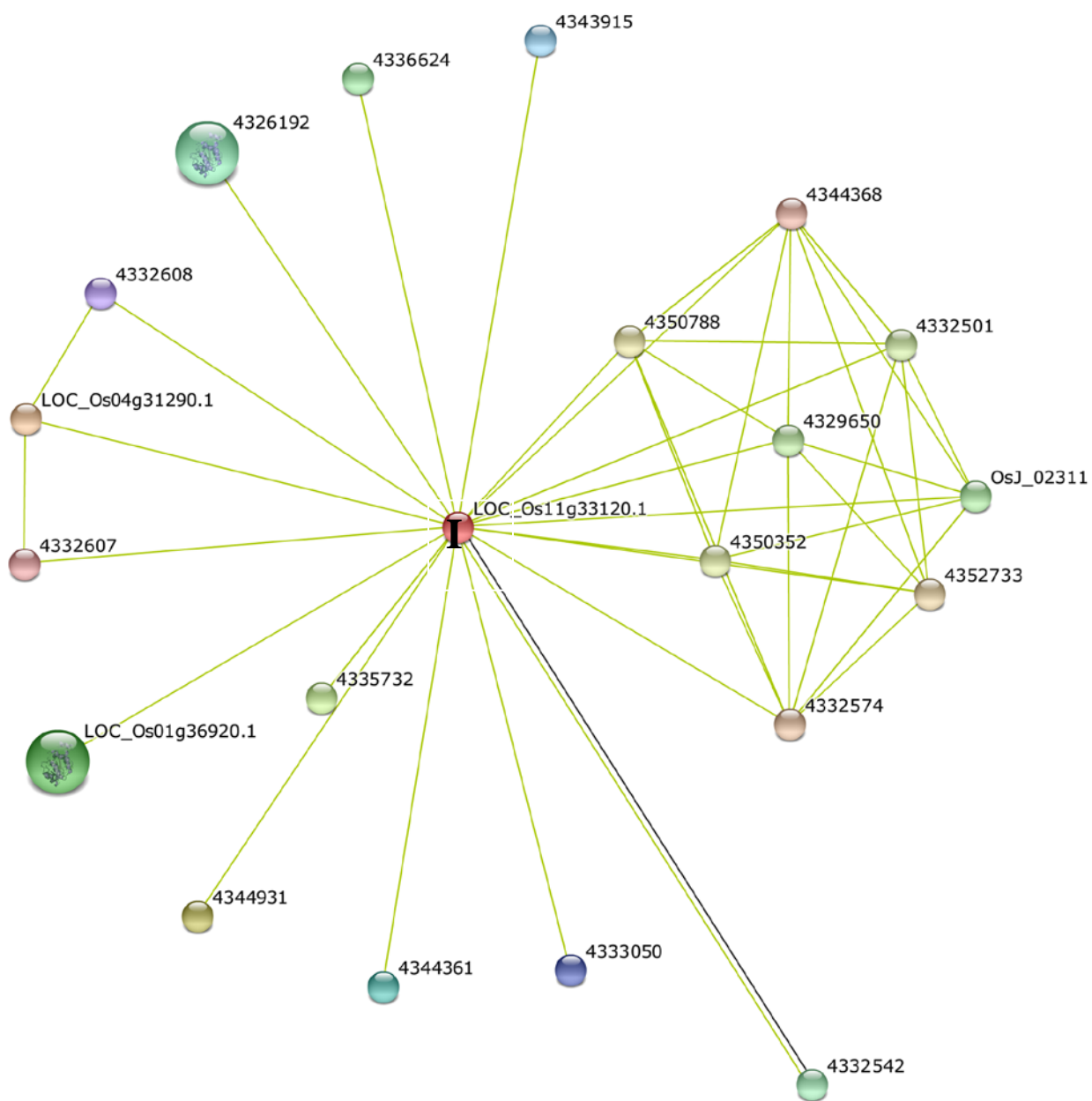

Supplement: Supplementary file 7 — Individual network diagram of OsRboh proteins showing potential interacting partners in evidence view using no more than 20 interactors option. Different coloured lines indicate types of evidence for association. The thickness of each line indicates the strength of the association. (PDF 855 kb) [file 12870_2018_1378_MOESM7_ESM.pdf]

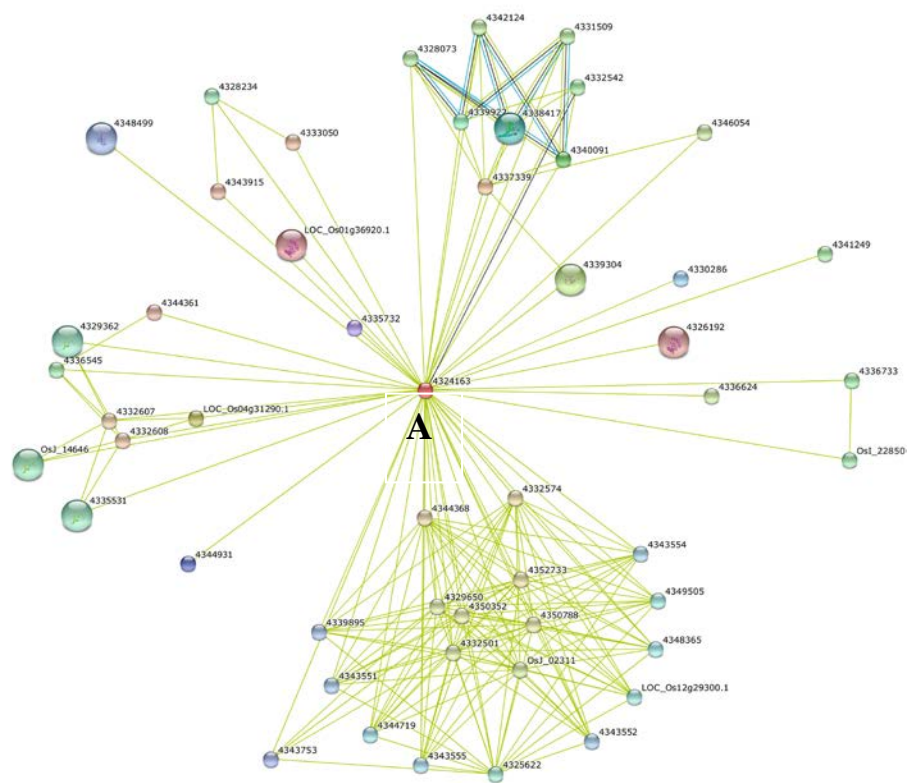

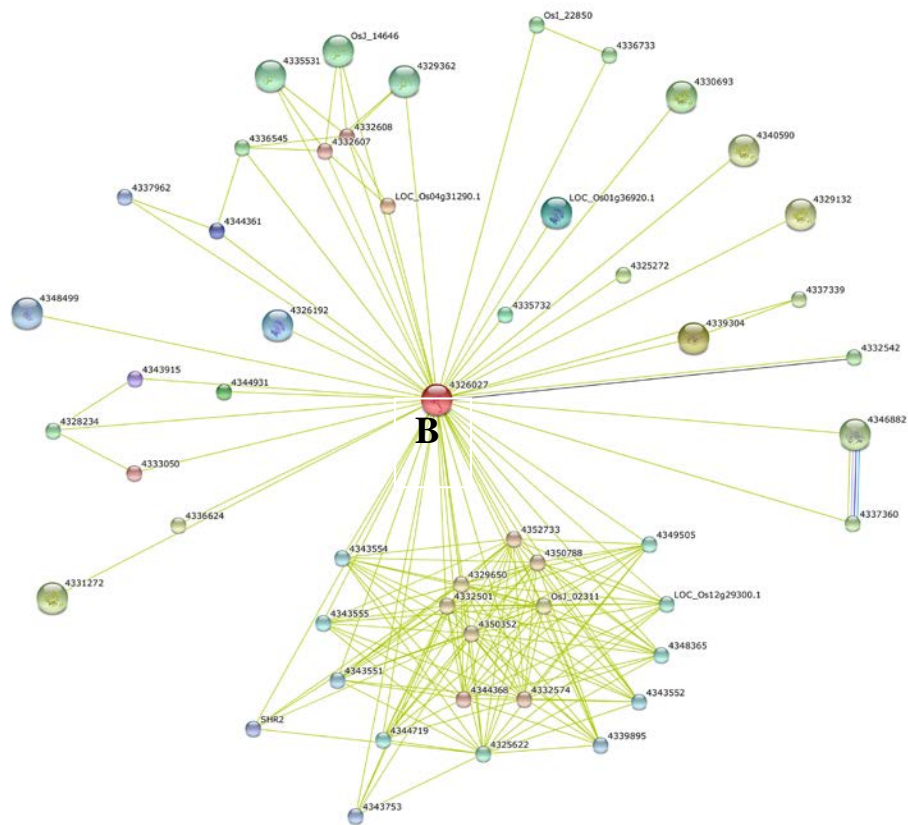

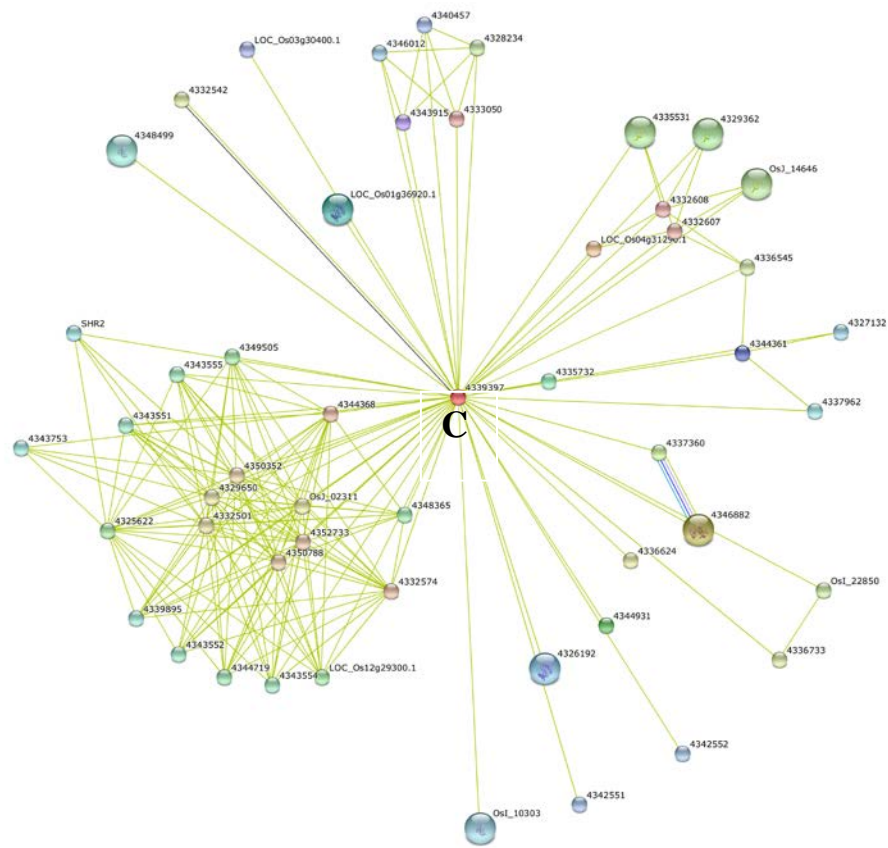

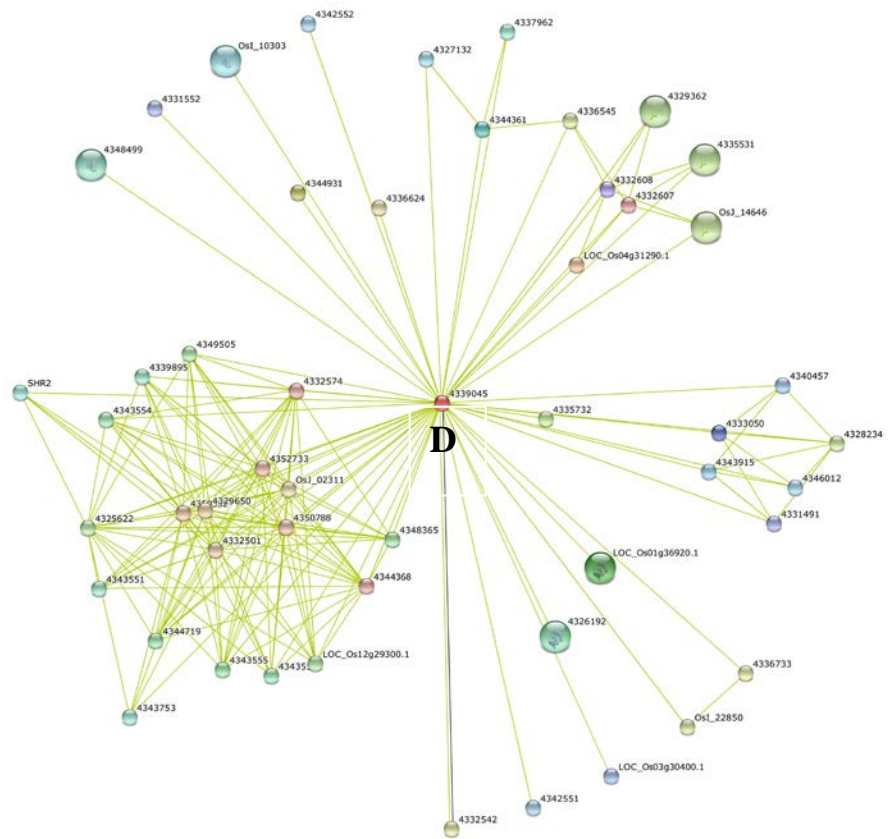

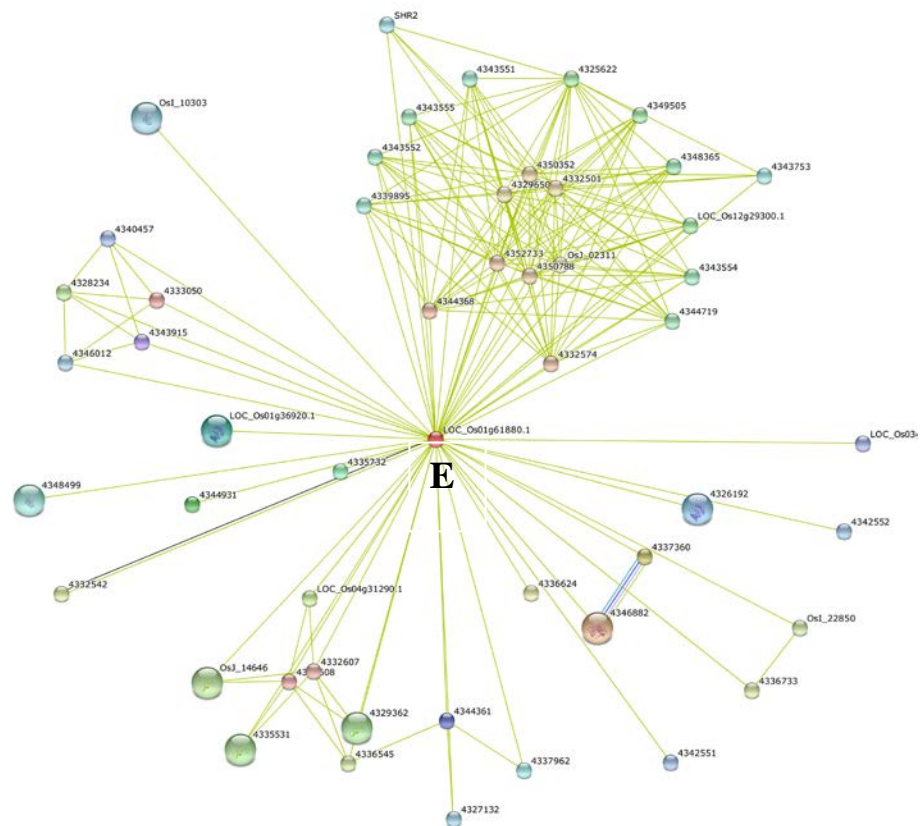

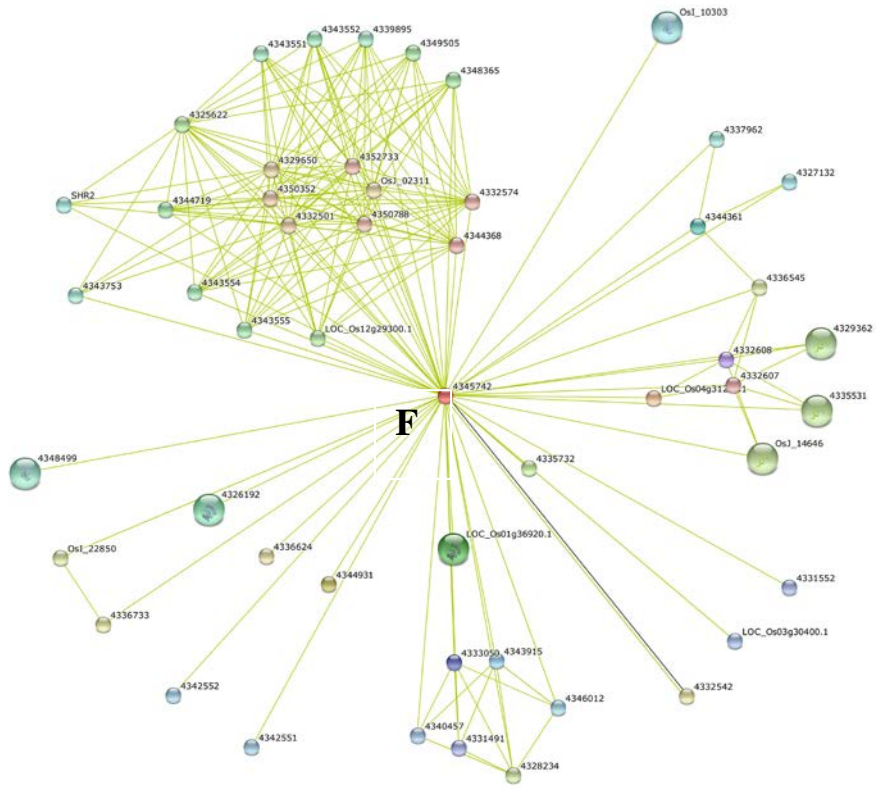

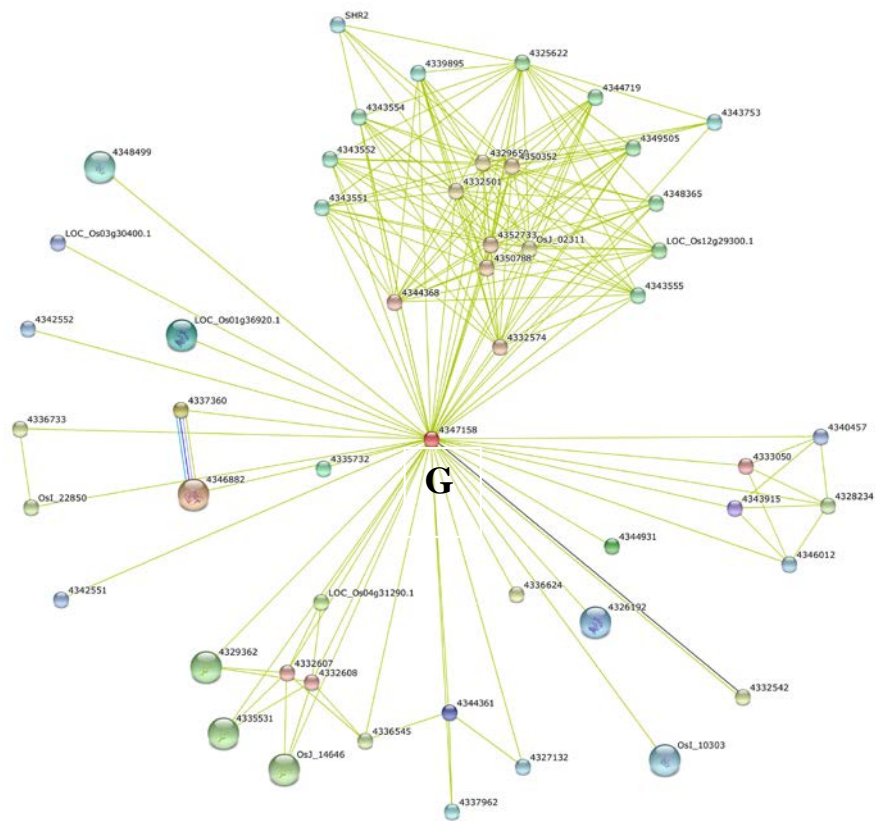

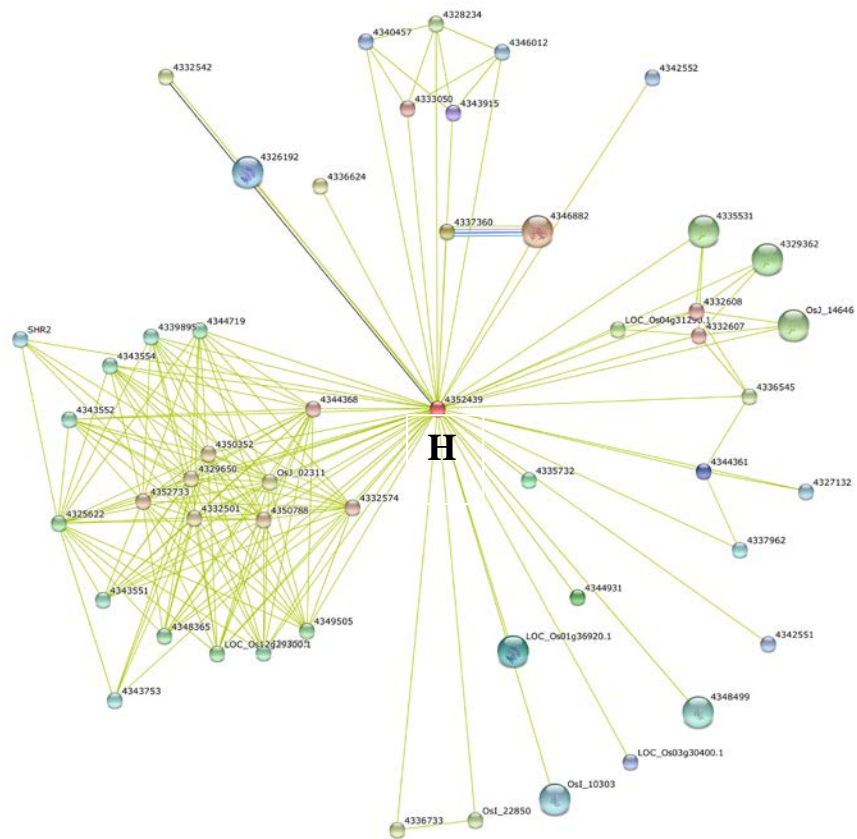

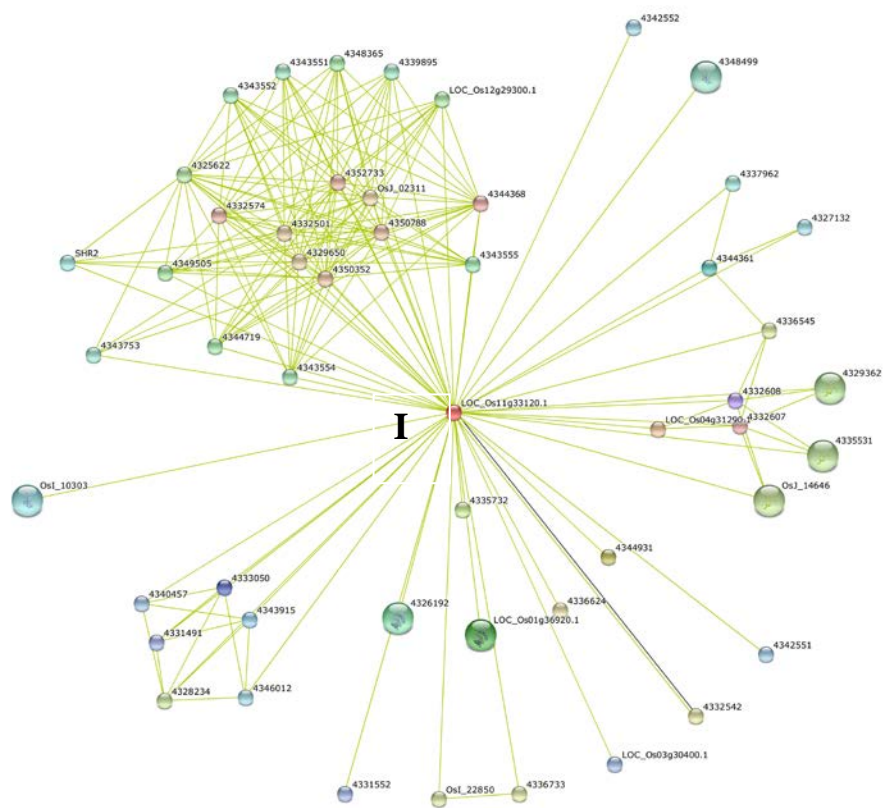

Supplement: Supplementary file 8 — Individual network diagram of OsRboh proteins showing potential interacting partners in evidence view using no more than 50 interactors option. Different coloured lines indicate types of evidence for association. The thickness of each line indicates the strength of the association. (PDF 491 kb) [file 12870_2018_1378_MOESM8_ESM.pdf]
