# Supplementary material for: In silico insights on diverse interacting partners and phosphorylation sites of respiratory burst oxidase homolog (Rbohs) gene families from Arabidopsis and rice
Source: BMC Plant Biol. 2018 Aug 10;18:161. doi: 10.1186/s12870-018-1378-2 (PMC6086027; doi:10.1186/s12870-018-1378-2)
Supplement: Supplementary file 4 — Table showing functional categorization of identified interaction partners among AtRbohs. (PDF 303 kb) [file 12870_2018_1378_MOESM4_ESM.pdf]

Table. Functional categorization of identified interaction partners among AtRbohs.

Categories based on functions of interaction partners	Identified interaction partners	Specific functions of interaction partners	References
**CELLULAR DEVELOPMENT**	TOPP7	Cell cycle regulation	[1]
	AT1G19840	Cell expansion	[2]
	ATEXPA2	Developmental processes	[3]
	SDH2-2	Electron transport	[4]
	ATRDH2, AGL52, AT1G17680, CDPK9	Embryo and seed development	[5-9]
	BTI2	ER organization	[10]
	AT1G26270	Inositol phospholipid metabolism	[11]
	AT2G17500	Photomorphogenesis	[12]
	ARAC6	Pollen tube growth	[13]
	AT5G09550	Protein transport	UniProt
	AT2G35040	Purine nucleotide biosynthesis	UniProt
	COW1, RHD4, RHD3, CSLD3, RHD1, SHV2, SHV3, ROP2, LRX1, MRH2	Root development	[14-21]
	AT4G33160, MT2B	Shoot development	[22,23]
	AT5G54130, AT3G28220	Starch metabolism	[24]
	CDPK19	Stomata	[6,9]
**ABIOTIC STRESS**	CPK1, CZF1, RAP2.1	Cold	[6,9,25]
	ATCDPK1, ATCDPK2, CPK5, CDPK6, CPK6, OST1, CPK4, CYP707A4, CPK21, AT1G76640	Drought	[6,9,26-28]
	AT3G61720, AILP1	Hypoxic	[29]
	ATCDPK1, ATCDPK2, CDPK6, CPK15, CPK6, AT1G76640	Salt	[6,9,28,30]
	UVH6	UV	[31]

	AT4G11270	Mercury	[32]
	MTPA2, HMA4	Zinc	[33]
**BIOTIC STRESS**	CPK1, CPK13, ATCDPK2, CPK5, CDPK6, CPK6, CPK4, CIPK25, AT1G76640, AT5G07580, AT3G28220, AT4G38220	Pathogen-responsive	[6,9,28,34-36]
**HORMONAL REGULATION**	PGP14, AT1G19840, AT2G17500	Auxin transport/induction	[2,37,38]
	GA20OX3	Gibberellin biosynthesis	[39]
	DGL	Jasmonic acid biosynthesis	[40]
	AT3G33530	Other hormone response	[41]
**UNKNOWN**	CPK26	Unknown	

References

1. Farkas I, Dombradi V, Miskei M, Szabados L, Koncz C (2007) Arabidopsis PPP family of serine/threonine phosphatases. Trends Plant Sci 12: 169-176.
2. Spartz AK, Lee SH, Wenger JP, Gonzalez N, Itoh H, et al. (2012) The SAUR19 subfamily of SMALL AUXIN UP RNA genes promote cell expansion. Plant J 70: 978-990.
3. Sampedro J, Cosgrove DJ (2005) The expansin superfamily. Genome Biol 6.
4. Elorza A, León G, Gómez I, Mouras A, Holuigue L, et al. (2004) Nuclear SDH2-1 and SDH2-2 genes, encoding the iron-sulfur subunit of mitochondrial complex II in Arabidopsis, have distinct cell-specific expression patterns and promoter activities. Plant Physiol 136: 4072-4087.
5. Bemer M, Heijmans K, Airoldi C, Davies B, Angenent GC (2010) An Atlas of Type I MADS Box Gene Expression during Female Gametophyte and Seed Development in Arabidopsis. Plant Physiol 154: 287-300.

6. Boudsocq M, Sheen J (2013) CDPKs in immune and stress signaling. Trends Plant Sci 18: 30-40.

7. Liu Y, Ye N, Liu R, Chen M, Zhang J (2010) H2O2 mediates the regulation of ABA catabolism and GA biosynthesis in Arabidopsis seed dormancy and germination. J Exp Bot 61: 2979-2990.

8. Mao GH, Wang RG, Guan YF, Liu YD, Zhang SQ (2011) Sulfurtransferases 1 and 2 Play Essential Roles in Embryo and Seed Development in Arabidopsis thaliana. J Biol Chem 286: 7548-7557.

9. Wurzinger B, Mair A, Pfister B, Teige M (2011) Cross-talk of calcium-dependent protein kinase and MAP kinase signaling. Plant Signal Behav 6: 8-12.

10. Nziengui H, Bouhidel K, Pillon D, Der C, Marty F, et al. (2007) Reticulon-like proteins in Arabidopsis thaliana: Structural organization and ER localization. FEBS Lett 581: 3356-3362.

11. Mueller-Roeber B, Pical C (2002) Inositol phospholipid metabolism in Arabidopsis. Characterized and putative isoforms of inositol phospholipid kinase and phosphoinositide-specific phospholipase C. Plant Physiol 130: 22-46.

12. Alabadi D, Gallego-Bartolome J, Orlando L, Garcia-Carcel L, Rubio V, et al. (2008) Gibberellins modulate light signaling pathways to prevent Arabidopsis seedling de-etiolation in darkness. Plant Journal 53: 324-335.

13. Craddock C, Lavagi I, Yang ZB (2012) New insights into Rho signaling from plant ROP/Rac GTPases. Trends Cell Biol 22: 492-501.

14. Baumberger N, Ringli C, Keller B (2001) The chimeric leucine-rich repeat/extensin cell wall protein LRX1 is required for root hair morphogenesis in Arabidopsis thaliana. Genes & Development 15: 1128-1139.

15. Böhme K, Li Y, Charlot F, Grierson C, Marrocco K, et al. (2004) The Arabidopsis COW1 gene encodes a phosphatidylinositol transfer protein essential for root hair tip growth. Plant Journal 40: 686-698.

16. Hayashi S, Ishii T, Matsunaga T, Tominaga R, Kuromori T, et al. (2008) The Glycerophosphoryl Diester Phosphodiesterase-Like Proteins SHV3 and its Homologs Play Important Roles in Cell Wall Organization. Plant Cell Physiol 49: 1522-1535.

17. Nguema-Ona E, Andeme-Onzighi C, Aboughe-Angone S, Bardor M, Ishii T, et al. (2006) The reb1-1 mutation of Arabidopsis. Effect on the structure and localization of galactose-containing cell wall polysaccharides. Plant Physiol 140: 1406-1417.

18. Thole JM, Vermeer JEM, Zhang YL, Gadella TWJ, Nielsen E (2008) ROOT HAIR DEFECTIVE4 encodes a phosphatidylinositol-4-phosphate phosphatase required for proper root hair development in Arabidopsis thaliana. Plant Cell 20: 381-395.

19. Wang X, Cnops G, Vanderhaeghen R, De Block S, Van Montagu M, et al. (2001) AtCSLD3, a cellulose synthase-like gene important for root hair growth in Arabidopsis. Plant Physiol 126: 575-586.

20. Yang GH, Gao P, Zhang H, Huang SJ, Zheng ZL (2007) A Mutation in MRH2 Kinesin Enhances the Root Hair Tip Growth Defect Caused by Constitutively Activated ROP2 Small GTPase in Arabidopsis. PLoS One 2.

21. Yuen CYL, Sedbrook JC, Perrin RM, Carroll KL, Masson PH (2005) Loss-of-function mutations of ROOT HAIR DEFECTIVE3 suppress root waving, skewing, and epidermal cell file rotation in Arabidopsis. Plant Physiol 138: 701-714.

22. Che P, Gingerich DJ, Lall S, Howell SH (2002) Global and hormone-induced gene expression changes during shoot development in Arabidopsis. Plant Cell 14: 2771-2785.

23. Di Giacomo E, Serino G, Frugis G (2013) Emerging Role of the Ubiquitin Proteasome System in the Control of Shoot Apical Meristem Function. J Integr Plant Biol 55: 7-20.
24. Smith SM, Fulton DC, Chia T, Thorneycroft D, Chapple A, et al. (2004) Diurnal changes in the transcriptome encoding enzymes of starch metabolism provide evidence for both transcriptional and posttranscriptional regulation of starch metabolism in Arabidopsis leaves. Plant Physiol 136: 2687-2699.
25. Vogel JT, Zarka DG, Van Buskirk HA, Fowler SG, Thomashow MF (2005) Roles of the CBF2 and ZAT12 transcription factors in configuring the low temperature transcriptome of Arabidopsis. Plant J 41: 195-211.
26. Geiger D, Scherzer S, Mumm P, Stange A, Marten I, et al. (2009) Activity of guard cell anion channel SLAC1 is controlled by drought-stress signaling kinase-phosphatase pair. Proc Natl Acad Sci U S A 106: 21425-21430.
27. Kushiro T, Okamoto M, Nakabayashi K, Yamagishi K, Kitamura S, et al. (2004) The Arabidopsis cytochrome P450 CYP707A encodes ABA 8'-hydroxylases: key enzymes in ABA catabolism. EMBO J 23: 1647-1656.
28. Vanderbeld B, Snedden WA (2007) Developmental and stimulus-induced expression patterns of Arabidopsis calmodulin-like genes CML37, CML38 and CML39. Plant Mol Biol 64: 683-697.
29. Liu F, VanToai T, Moy LP, Bock G, Linford LD, et al. (2005) Global transcription profiling reveals comprehensive insights into hypoxic response in Arabidopsis. Plant Physiol 137: 1115-1129.
30. Yokotani N, Ichikawa T, Kondou Y, Matsui M, Hirochika H, et al. (2009) Tolerance to various environmental stresses conferred by the salt-responsive rice gene ONAC063 in transgenic Arabidopsis. Planta 229: 1065-1075.

31. Liu ZR, Hong SW, Escobar M, Vierling E, Mitchell DL, et al. (2003) Arabidopsis UVH6, a homolog of human XPD and yeast RAD3 DNA repair genes, functions in DNA repair and is essential for plant growth. Plant Physiol 132: 1405-1414.

32. Heidenreich B, Mayer K, Sandermann H, Ernst D (2001) Mercury-induced genes in Arabidopsis thaliana: identification of induced genes upon long-term mercuric ion exposure. Plant Cell Environ 24: 1227-1234.

33. Roosens NHCJ, Willems G, Saumitou-Laprade P (2008) Using Arabidopsis to explore zinc tolerance and hyperaccumulation. Trends Plant Sci 13: 208-215.

34. Chen LG, Zhang LP, Li DB, Wang F, Yu DQ (2013) WRKY8 transcription factor functions in the TMV-cg defense response by mediating both abscisic acid and ethylene signaling in Arabidopsis. Proc Natl Acad Sci U S A 110: E1963-E1971.

35. Peškan-Berghöfer T, Shahollari B, Giong PH, Hehl S, Markert C, et al. (2004) Association of Piriformospora indica with Arabidopsis thaliana roots represents a novel system to study beneficial plant–microbe interactions and involves early plant protein modifications in the endoplasmic reticulum and at the plasma membrane. Physiol Plant 122: 465-477.

36. Siemens J, Keller I, Sarx J, Kunz S, Schuller A, et al. (2006) Transcriptome analysis of Arabidopsis clubroots indicate a key role for cytokinins in disease development. Mol Plant Microbe Interact 19: 480-494.

37. Sanchez-Fernandez R, Davies TGE, Coleman JOD, Rea PA (2001) The Arabidopsis thaliana ABC protein superfamily, a complete inventory. J Biol Chem 276: 30231-30244.

38. Zhang H, Kim MS, Krishnamachari V, Payton P, Sun Y, et al. (2007) Rhizobacterial volatile emissions regulate auxin homeostasis and cell expansion in Arabidopsis. Planta 226: 839-851.

39. Han FM, Zhu BG (2011) Evolutionary analysis of three gibberellin oxidase genesin rice, Arabidopsis, and soybean. Gene 473: 23-35.

40. Ellinger D, Stingl N, Kubigsteltig II, Bals T, Juenger M, et al. (2010) DONGLE and DEFECTIVE IN ANTHER DEHISCENCE1 lipases are not essential for wound-and pathogen-induced jasmonate biosynthesis: redundant lipases contribute to jasmonate formation. Plant Physiol 153: 114-127.

41. Deihimi T, Niazi A, Ebrahimi M, Kajbaf K, Fanaee S, et al. (2012) Finding the undiscovered roles of genes: an approach using mutual ranking of coexpressed genes and promoter architecture-case study: dual roles of thaumatin like proteins in biotic and abiotic stresses. Springerplus 1: 1-10.
