# Supplementary material for: In silico insights on diverse interacting partners and phosphorylation sites of respiratory burst oxidase homolog (Rbohs) gene families from Arabidopsis and rice
Source: BMC Plant Biol. 2018 Aug 10;18:161. doi: 10.1186/s12870-018-1378-2 (PMC6086027; doi:10.1186/s12870-018-1378-2)
Supplement: Supplementary file 6 — Individual network diagrams of 9 OsRboh proteins showing potential interacting partners in evidence view with 10 interactors. Different coloured lines indicate types of evidence for association. The thickness of each line indicates the strength of the association. (PDF 1978 kb) [file 12870_2018_1378_MOESM6_ESM.pdf]

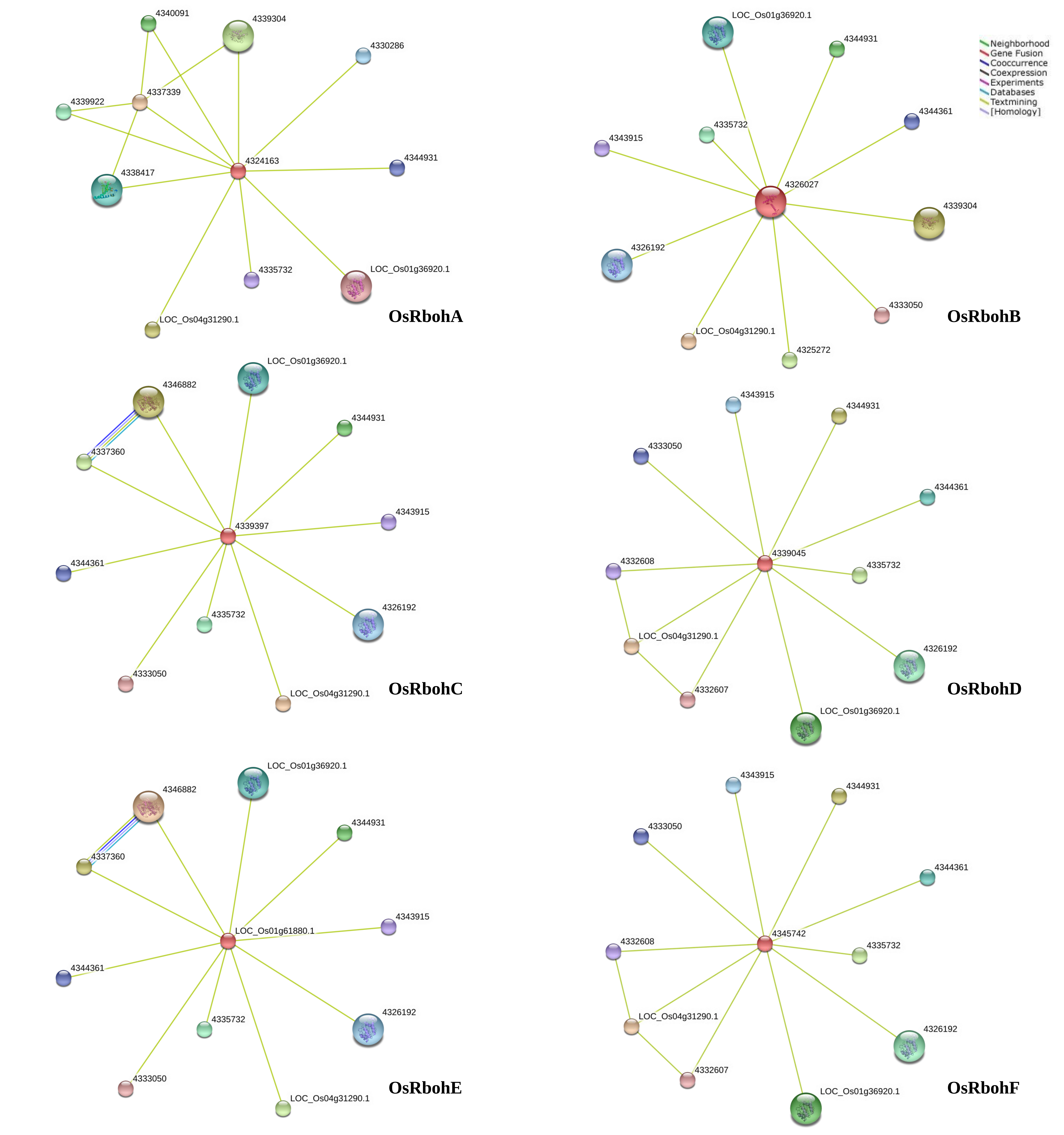
4340091
4339304
4330286
4339922
4337339
4324163
4344931
4338417
4335732
LOC_Os01g36920.1
LOC_Os04g31290.1
OsRbohA
LOC_Os01g36920.1
4344931
Neighborhood
Gene Fusion
Cooccurrence
Coexpression
Experiments
Databases
Textmining
[Homology]
4344361
4335732
4343915
4326027
4339304
4326192
4333050
LOC_Os04g31290.1
4325272
OsRbohB
LOC_Os01g36920.1
4346882
4344931
4337360
4343915
4339397
4344361
4326192
4335732
4333050
LOC_Os04g31290.1
OsRbohC
4343915
4344931
4333050
4344361
4339045
4332608
4335732
LOC_Os04g31290.1
4326192
4332607
LOC_Os01g36920.1
OsRbohD
LOC_Os01g36920.1
4346882
4344931
4337360
4343915
LOC_Os01g61880.1
4344361
4326192
4335732
4333050
LOC_Os04g31290.1
OsRbohE
4343915
4344931
4333050
4344361
4345742
4332608
4335732
LOC_Os04g31290.1
4326192
4332607
LOC_Os01g36920.1
OsRbohF

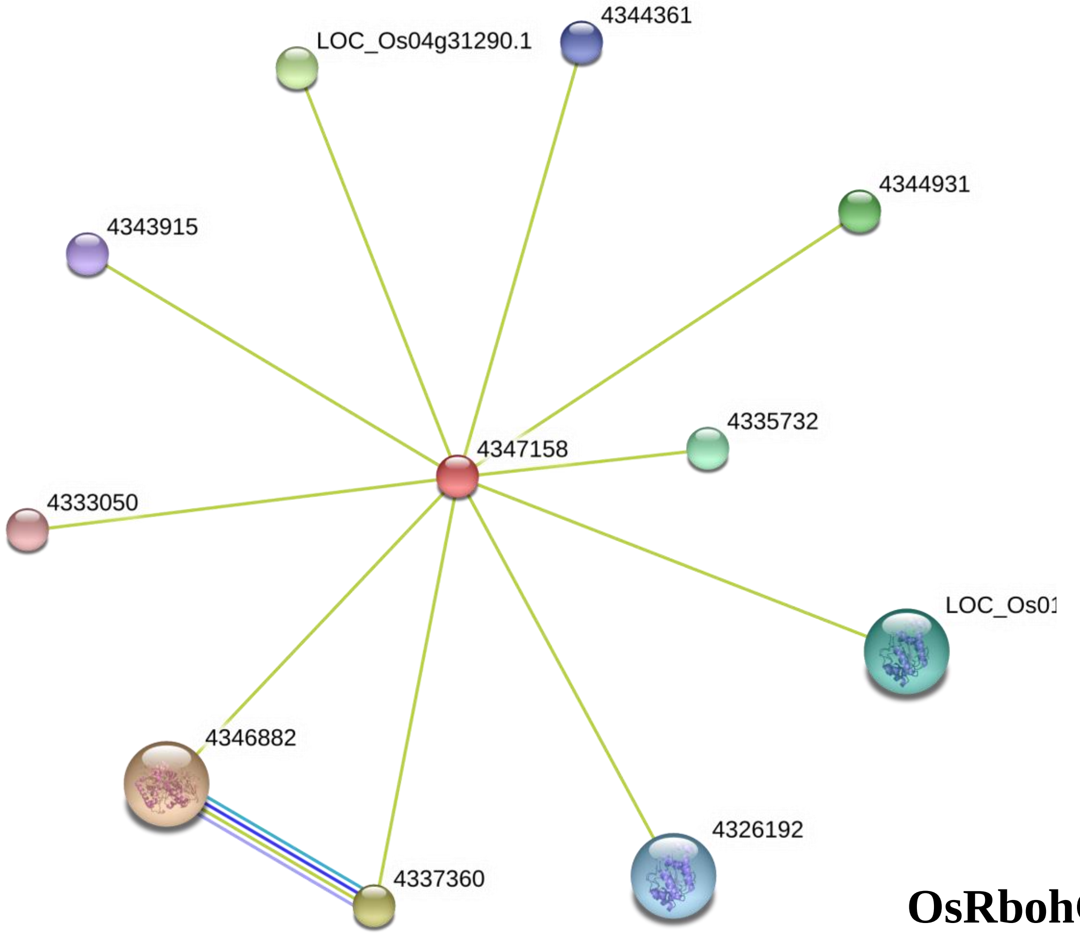

OsRbohG

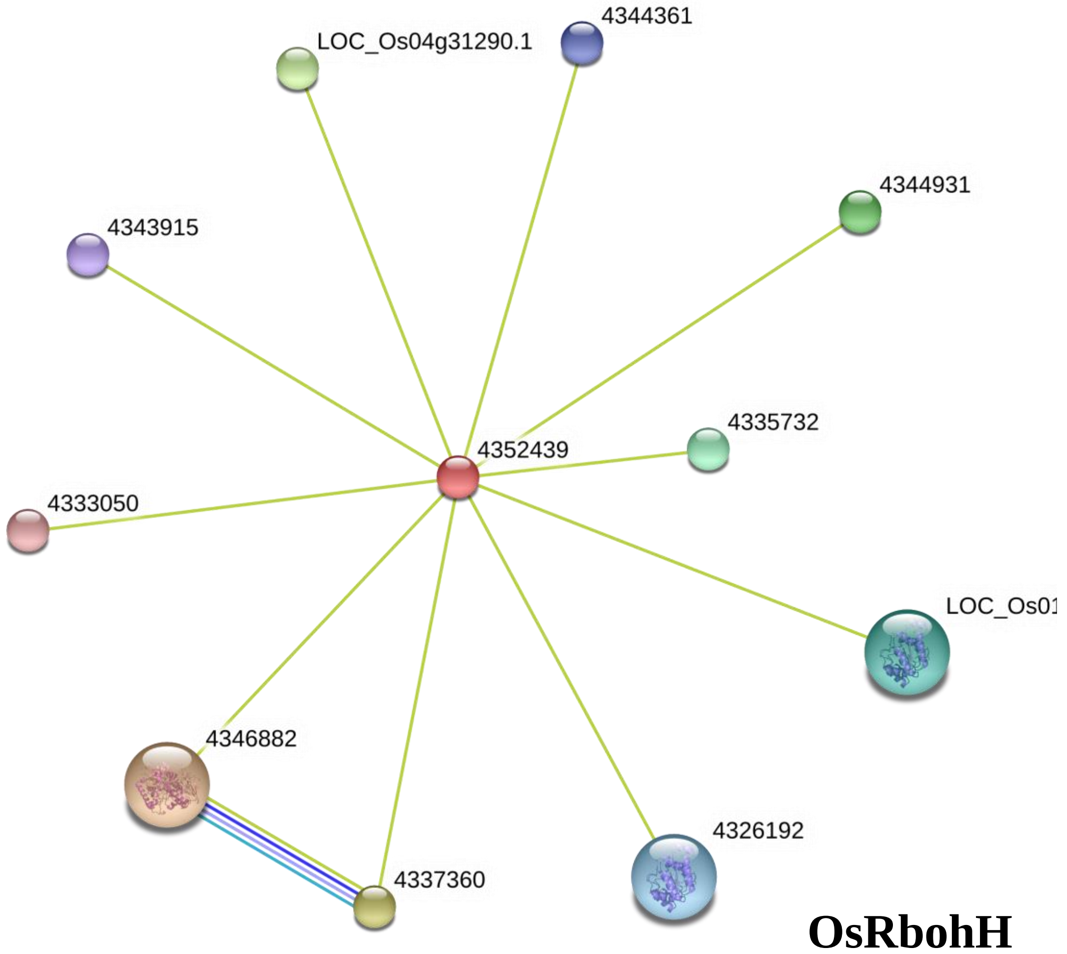

OsRbohH

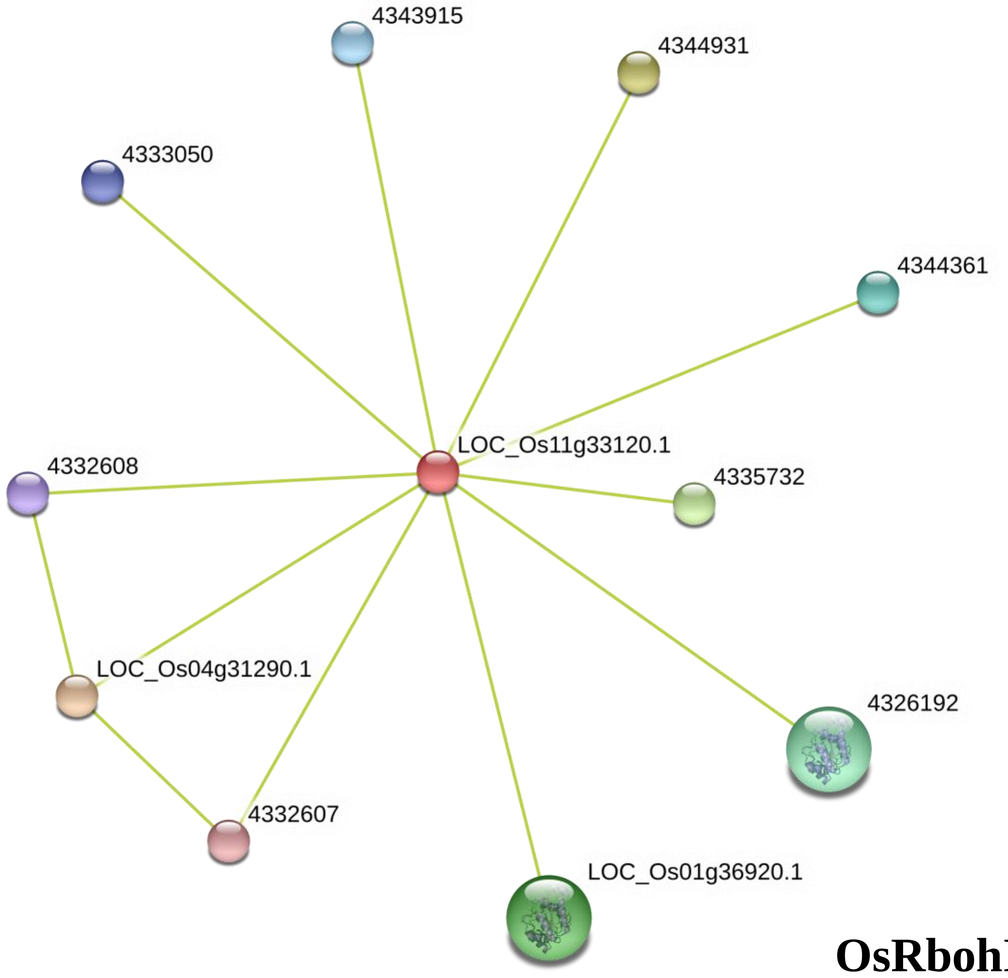

OsRbohI
