# Supplementary material for: In silico insights on diverse interacting partners and phosphorylation sites of respiratory burst oxidase homolog (Rbohs) gene families from Arabidopsis and rice
Source: BMC Plant Biol. 2018 Aug 10;18:161. doi: 10.1186/s12870-018-1378-2 (PMC6086027; doi:10.1186/s12870-018-1378-2)
Supplement: Supplementary file 9 — Table showing details of identified potential functional partners of OsRboh proteins. (PDF 259 kb) [file 12870_2018_1378_MOESM9_ESM.pdf]

Table. Details of identified potential functional partners of OsRboh proteins.

Rbohs	Functional Partners						Types of evidence for the association							Score
	**S.No.**	**Name**	**Gene ID**	**Description**	**UniProt ID**	**Length (aa)**	**Neighborhood**	**Gene Fusion**	**Co- occurrence**	**Experiments**	**Databases**	**Textmining**	**Homology**	
OsRbohA	1.	4337339	LOC_Os04g57320.1	Immutans protein (ubiquinol oxidase)	B8AW16	336						P		0.75
	2.	LOC_Os04g31290.1	LOC_Os04g31290.1	Basic helix-loop-helix (bHLH) DNA-binding domain containing protein	Q01LJ4	352						P		0.625
	3.	4339304	LOC_Os05g43820.1	Ras-related protein, belongs to the small GTPase superfamily	A2Y6G9	214						P		0.571
	4.	4340091	LOC_Os06g05110.1	Superoxide dismutase; chloroplastic	B8B2C9	255						P		0.548
	5.	4339922	LOC_Os06g02500.1	Superoxide dismutase; chloroplastic	B8B1M4	391						P		0.542
	6.	4338417	LOC_Os05g25850.1	superoxide dismutase [Mn]; mitochondrial	Q43008*	231						P		0.54
	7.	4330286	LOC_Os02g45130.1	Probable serine/threonine-protein kinase WNK4	Q6EU49*	612						P		0.54
	8.	4344931	LOC_Os08g10570.1	Bifunctional purine biosynthesis protein purH;IMP cyclohydrolase activity;	B8BBZ9	601						P		0.529

				phosphoribosylaminoimidazolecarboxamide formyltransferase activity										
	9.	4335732	LOC_Os04g32950.1	Calreticulin precursor protein (calnexin)	Q7XV86	537						P		0.529
	10.	LOC_Os01g36920.1	LOC_Os01g36920.1	DEAD-box ATP-dependent RNA helicase 15	B7F820	432						P		0.52
OsRbohB	1.	LOC_Os04g31290.1	LOC_Os04g31290.1	Basic helix-loop-helix (bHLH) DNA-binding domain containing protein	Q01LJ4	352						P		0.625
	2.	4339304	LOC_Os05g43820.1	Ras-related protein, belongs to the small GTPase superfamily	A2Y6G9	214						P		0.55
	3.	4325272	LOC_Os01g48680.1	Two pore calcium channel protein 1	Q5QM84*	757						P		0.54
	4.	4344931	LOC_Os08g10570.1	Bifunctional purine biosynthesis protein purH;IMP cyclohydrolase activity; phosphoribosylaminoimidazolecarboxamide formyltransferase activity	B8BBZ9	601						P		0.529
	5.	4335732	LOC_Os04g32950.1	Calreticulin precursor protein (calnexin)	Q7XV86	537						P		0.529
	6.	LOC_Os01g36920.1	LOC_Os01g36920.1	DEAD-box ATP-dependent RNA helicase 15	B7F820	432						P		0.52
	7.	4326192	LOC_Os01g36890.1	DEAD-box ATP-dependent RNA helicase	B8AA09	432						P		0.52

	8.	4344361	LOC_Os07g48980.1	Nicotianamine synthase 3; involved in long-distance transport of iron and differentially regulated by iron	A2YQ58*	343					P		0.512
	9.	4343915	LOC_Os07g42590.1	OsFBX258- F-box domain containing protein	A2YNQ9	406					P		0.511
	10.	4333050	LOC_Os03g28130.1	OsFBX94 - F-box domain containing protein	A2XHP5	408					P		0.511
OsRbohC	1.	LOC_Os0.4g31290.1	LOC_Os04g31290.1	Basic helix-loop-helix (bHLH) DNA-binding domain containing protein	Q01LJ4	352					P		0.625
	2.	4346882	LOC_Os09g20284.1	Amino oxidase	A2Z0H1	478					P		0.545
	3.	4337360	LOC_Os04g57560.1	Amino oxidase	B8ARE0	492					P		0.545
	4.	4344931	LOC_Os08g10570.1	Bifunctional purine biosynthesis protein purH;IMP cyclohydrolase activity; phosphoribosylaminoimidazolecarboxamide formyltransferase activity	B8BBZ9	601					P		0.529
	5.	4335732	LOC_Os04g32950.1	Calreticulin precursor protein (calnexin)	Q7XV86	537					P		0.529
	6.	LOC_Os01g36920.1	LOC_Os01g36920.1	DEAD-box ATP-dependent RNA helicase 15	B7F820	432					P		0.52
	7.	4326192	LOC_Os01g36890.1	DEAD-box ATP-dependent RNA helicase	B8AA09	432					P		0.52
	8.	4344361	LOC_Os07g48980.1	Nicotianamine synthase 3; involved in long-distance	A2YQ58*	343					P		0.512

				transport of iron and differentially regulated by iron										
	9.	4343915	LOC_Os07g42590.1	OsFBX258- F-box domain containing protein	A2YNQ9	406						P		0.511
	10.	4333050	LOC_Os03g28130.1	OsFBX94 - F-box domain containing protein	A2XHP5	408						P		0.511
OsRbohD	1.	LOC_Os04g31290.1	LOC_Os04g31290.1	Basic helix-loop-helix (bHLH) DNA-binding domain containing protein	Q01LJ4	352						P		0.625
	2.	4344931	LOC_Os08g10570.1	Bifunctional purine biosynthesis protein purH;IMP cyclohydrolase .activity; phosphoribosylaminoimidazolecarboxamide formyltransferase activity	B8BBZ9	601						P		0.529
	3.	4335732	LOC_Os04g32950.1	Calreticulin precursor protein (calnexin)	Q7XV86	537						P		0.529
	4.	LOC_Os01g36920.1	LOC_Os01g36920.1	DEAD-box ATP-dependent RNA helicase 15	B7F820	432						P		0.52
	5.	4326192	LOC_Os01g36890.1	DEAD-box ATP-dependent RNA helicase	B8AA09	432						P		0.52
	6.	4344361	LOC_Os07g48980.1	Nicotianamine synthase 3; involved in long-distance transport of iron and differentially regulated by iron	A2YQ58*	343						P		0.512
	7.	4343915	LOC_Os07g42590.1	OsFBX258- F-box domain containing protein	A2YNQ9	406						P		0.511

	8.	4333050	LOC_Os03g28130.1	OsFBX94 - F-box domain containing protein	A2XHP5	408						P		0.511
	9.	4332608	LOC_Os03g19427.1	Nicotianamine synthase 1; involved in long-distance transport of iron and differentially regulated by iron	A2XFU4*	332						P		0.511
	10.	4332607	LOC_Os03g19420.2	Nicotianamine synthase 2; involved in long-distance transport of iron and differentially regulated by iron	A2XFU5*	326						P		0.511
OsRbohE	1.	4346882	LOC_Os09g20284.1	Amino oxidase	A2Z0H1	478						P		0.635
	2.	4337360	LOC_Os04g57560.1	Amino oxidase	B8ARE0	492						P		0.635
	3.	LOC_Os04g31290.1	LOC_Os04g31290.1	Basic helix-loop-helix (bHLH) DNA-binding domain containing protein	Q01LJ4	352						P		0.625
	4.	4344931	LOC_Os08g10570.1	Bifunctional purine biosynthesis protein purH;IMP cyclohydrolase activity; phosphoribosylaminoimidazolecarboxamide formyltransferase activity	B8BBZ9	601						P		0.529
	5.	4335732	LOC_Os04g32950.1	Calreticulin precursor protein (calnexin)	Q7XV86	537						P		0.529
	6.	LOC_Os01g36920.1	LOC_Os01g36920.1	DEAD-box ATP-dependent RNA helicase 15	B7F820	432						P		0.52
	7.	4326192	LOC_Os01g36890.1	DEAD-box ATP-dependent RNA helicase	B8AA09	432						P		0.52

	8.	4344361	LOC_Os07g48980.1	Nicotianamine synthase 3; involved in long-distance transport of iron and differentially regulated by iron	A2YQ58*	343						P		0.512
	9.	4343915	LOC_Os07g42590.1	OsFBX258- F-box domain containing protein	A2YNQ9	406						P		0.511
	10.	4333050	LOC_Os03g28130.1	OsFBX94 - F-box domain containing protein	A2XHP5	408						P		0.511
OsRbohF	1.	LOC_Os04g31290.1	LOC_Os04g31290.1	Basic helix-loop-helix (bHLH) DNA-binding domain containing protein	Q01LJ4	352						P		0.625
	2.	4344931	LOC_Os08g10570.1	Bifunctional purine biosynthesis protein purH;IMP cyclohydrolase activity; phosphoribosylaminoimidazolecarboxamide formyltransferase activity	B8BBZ9	601						P		0.529
	3.	4335732	LOC_Os04g32950.1	Calreticulin precursor protein (calnexin)	Q7XV86	537						P		0.529
	4.	LOC_Os01g36920.1	LOC_Os01g36920.1	DEAD-box ATP-dependent RNA helicase 15	B7F820	432						P		0.52
	5.	4326192	LOC_Os01g36890.1	DEAD-box ATP-dependent RNA helicase	B8AA09	432						P		0.52
	6.	4344361	LOC_Os07g48980.1	Nicotianamine synthase 3; involved in long-distance transport of iron and differentially regulated by iron	A2YQ58*	343						P		0.512

	7.	4343915	LOC_Os07g42590.1	OsFBX258- F-box domain containing protein	A2YNQ9	406						P		0.511
	8.	4333050	LOC_Os03g28130.1	OsFBX94 - F-box domain containing protein	A2XHP5	408						P		0.511
	9.	4332608	LOC_Os03g19427.1	Nicotianamine synthase 1; involved in long-distance transport of iron and differentially regulated by iron	A2XFU4*	332						P		0.511
	10.	4332607	LOC_Os03g19420.2	Nicotianamine synthase 2; involved in long-distance transport of iron and differentially regulated by iron	A2XFU5*	326						P		0.511
OsRbohG	1.	4346882	LOC_Os09g20284.1	Amino oxidase	A2Z0H1	478						P		0.635
	2.	4337360	LOC_Os04g57560.1	Amino oxidase	B8ARE0	492						P		0.635
	3.	LOC_Os04g31290.1	LOC_Os04g31290.1	Basic helix-loop-helix (bHLH) DNA-binding domain containing protein	Q01LJ4	352						P		0.625
	4.	4344931	LOC_Os08g10570.1	Bifunctional purine biosynthesis protein purH;IMP cyclohydrolase activity; phosphoribosylaminoimidazolecarboxamide formyltransferase activity	B8BBZ9	601						P		0.529
	5.	4335732	LOC_Os04g32950.1	Calreticulin precursor protein (calnexin)	Q7XV86	537						P		0.529
	6.	LOC_Os01g36920.1	LOC_Os01g36920.1	DEAD-box ATP-dependent RNA helicase 15	B7F820	432						P		0.52

	7.	4326192	LOC_Os01g36890.1	DEAD-box ATP-dependent RNA helicase	B8AA09	432						P		0.52
	8.	4344361	LOC_Os07g48980.1	Nicotianamine synthase 3; involved in long-distance transport of iron and differentially regulated by iron	A2YQ58*	343						P		0.512
	9.	4343915	LOC_Os07g42590.1	OsFBX258- F-box domain containing protein	A2YNQ9	406						P		0.511
	10.	4333050	LOC_Os03g28130.1	OsFBX94 - F-box domain containing protein	A2XHP5	408						P		0.511
OsRbohH	1.	4346882	LOC_Os09g20284.1	Amino oxidase	A2Z0H1	478						P		0.635
	2.	4337360	LOC_Os04g57560.1	Amino oxidase	B8ARE0	492						P		0.635
	3.	LOC_Os04g31290.1	LOC_Os04g31290.1	Basic helix-loop-helix (bHLH) DNA-binding domain containing protein	Q01LJ4	352						P		0.625
	4.	4344931	LOC_Os08g10570.1	Bifunctional purine biosynthesis protein purH;IMP cyclohydrolase activity; phosphoribosylaminoimidazolecarboxamide formyltransferase activity	B8BBZ9	601						P		0.529
	5.	4335732	LOC_Os04g32950.1	Calreticulin precursor protein (calnexin)	Q7XV86	537						P		0.529
	6.	LOC_Os01g36920.1	LOC_Os01g36920.1	DEAD-box ATP-dependent RNA helicase 15	B7F820	432						P		0.52
	7.	4326192	LOC_Os01g36890.1	DEAD-box ATP-dependent RNA	B8AA09	432						P		0.52

				helicase										
	8.	4344361	LOC_Os07g48980.1	Nicotianamine synthase 3; involved in long-distance transport of iron and differentially regulated by iron	A2YQ58*	343						P		0.512
	9.	4343915	LOC_Os07g42590.1	OsFBX258- F-box domain containing protein	A2YNQ9	406						P		0.511
	10.	4333050	LOC_Os03g28130.1	OsFBX94 - F-box domain containing protein	A2XHP5	408						P		0.511
OsRbohI	1.	LOC_Os04g31290.1	LOC_Os04g31290.1	Basic helix-loop-helix (bHLH) DNA-binding domain containing protein	Q01LJ4	352						P		0.625
	2.	4344931	LOC_Os08g10570.1	Bifunctional purine biosynthesis protein purH;IMP cyclohydrolase activity; phosphoribosylaminoimidazolecarboxamide formyltransferase activity	B8BBZ9	601						P		0.529
	3.	4335732	LOC_Os04g32950.1	Calreticulin precursor protein (calnexin)	Q7XV86	537						P		0.529
	4.	LOC_Os01g36920.1	LOC_Os01g36920.1	DEAD-box ATP-dependent RNA helicase 15	B7F820	432						P		0.52
	5.	4326192	LOC_Os01g36890.1	DEAD-box ATP-dependent RNA helicase	B8AA09	432						P		0.52
	6.	4344361	LOC_Os07g48980.1	Nicotianamine synthase 3; involved in long-distance transport of iron and differentially	A2YQ58*	343						P		0.512

				regulated by iron										
	7.	4343915	LOC_Os07g42590.1	OsFBX258- F-box domain containing protein	A2YNQ9	406						P		0.511
	8.	4333050	LOC_Os03g28130.1	OsFBX94 - F-box domain containing protein	A2XHP5	408						P		0.511
	9.	4332608	LOC_Os03g19427.1	Nicotianamine synthase 1; involved in long-distance transport of iron and differentially regulated by iron	A2XFU4*	332						P		0.511
	10.	4332607	LOC_Os03g19420.2	Nicotianamine synthase 2; involved in long-distance transport of iron and differentially regulated by iron	A2XFU5*	326						P		0.511

*: Reviewed UniProt ID; P: Present
