# Supplementary material for: In silico insights on diverse interacting partners and phosphorylation sites of respiratory burst oxidase homolog (Rbohs) gene families from Arabidopsis and rice
Source: BMC Plant Biol. 2018 Aug 10;18:161. doi: 10.1186/s12870-018-1378-2 (PMC6086027; doi:10.1186/s12870-018-1378-2)
Supplement: Supplementary file 10 — Table showing various unique and common functional partners among OsRbohs in color coding, where unique partners are uncoloured. (PDF 101 kb) [file 12870_2018_1378_MOESM10_ESM.pdf]

**Table.** Various unique and common functional partners among OsRbohs in color coding, where unique partners are uncoloured.

| **Rbohs** | **OsRbohA** | **OsRbohB** | **OsRbohC** | **OsRbohD** | **OsRbohE** | **OsRbohF** | **OsRbohG** | OsRbohH | OsRbohI |
|---|---|---|---|---|---|---|---|---|---|
| **Functional Partners** | 4337339 | LOC_Os04g31290.1 | LOC_Os04g31290.1 | LOC_Os04g31290.1 | 4346882 | LOC_Os04g31290.1 | 4346882 | 4346882 | LOC_Os04g31290.1 |
| | LOC_Os04g31290.1 | 4339304 | 4346882 | 4344931 | 4337360 | 4344931 | 4337360 | 4337360 | 4344931 |
| | 4339304 | 4325272 | 4337360 | 4335732 | LOC_Os04g31290.1 | 4335732 | LOC_Os04g31290.1 | LOC_Os04g31290.1 | 4335732 |
| | 4340091 | 4344931 | 4344931 | LOC_Os01g36920.1 | 4344931 | LOC_Os01g36920.1 | 4344931 | 4344931 | LOC_Os01g36920.1 |
| | 4339922 | 4335732 | 4335732 | 4326192 | 4335732 | 4326192 | 4335732 | 4335732 | 4326192 |
| | 4338417 | LOC_Os01g36920.1 | LOC_Os01g36920.1 | 4344361 | LOC_Os01g36920.1 | 4344361 | LOC_Os01g36920.1 | LOC_Os01g36920.1 | 4344361 |
| | 4330286 | 4326192 | 4326192 | 4343915 | 4326192 | 4343915 | 4326192 | 4326192 | 4343915 |
| | 4344931 | 4344361 | 4344361 | 4333050 | 4344361 | 4333050 | 4344361 | 4344361 | 4333050 |
| | 4335732 | 4343915 | 4343915 | 4332608 | 4343915 | 4332608 | 4343915 | 4343915 | 4332608 |
| | LOC_Os01g36920.1 | 4333050 | 4333050 | 4332607 | 4333050 | 4332607 | 4333050 | 4333050 | 4332607 |
