# Supplementary material for: In silico insights on diverse interacting partners and phosphorylation sites of respiratory burst oxidase homolog (Rbohs) gene families from Arabidopsis and rice
Source: BMC Plant Biol. 2018 Aug 10;18:161. doi: 10.1186/s12870-018-1378-2 (PMC6086027; doi:10.1186/s12870-018-1378-2)
Supplement: Supplementary file 11 — Table showing functional categorization of identified interaction partners among OsRbohs. (PDF 196 kb) [file 12870_2018_1378_MOESM11_ESM.pdf]

Table. Functional categorization of identified interaction partners among OsRbohs

Categories based on functions of interaction partners	Identified interaction partners	Specific functions of interaction partners	References
**CELLULAR DEVELOPMENT**	4338417	Embryogenesis	[1]
	4325272	Growth and development	[2]
	4344361, 4332608, 4332607	Iron transport	[3]
**ABIOTIC STRESS**	4340091, 4339922, 4338417, 434688	Drought	[4,5]
	4337339	High light	[6]
	4339304, LOC_Os01g36920.1, 4326192, 4346882, 4337360	Salt	[5,7-9]
**BIOTIC STRESS**	4335732	Pathogen-responsive	[10]
**UNKNOWN**	4330286, 4344931, 4343915, 4333050, LOC_Os04g31290.1	Unknown	

References

1. Zi J, Zhang J, Wang Q, Zhou B, Zhong J, et al. (2013) Stress responsive proteins are actively regulated during rice (Oryza sativa) embryogenesis as indicated by quantitative proteomics analysis. PLoS One 8: e74229.

2. Kurusu T, Sakurai Y, Miyao A, Hirochika H, Kuchitsu K (2004) Identification of a putative voltage-gated Ca2+-permeable channel (OsTPC1) involved in Ca2+ influx and regulation of growth and development in rice. Plant Cell Physiol 45: 693-702.

3. Inoue H, Higuchi K, Takahashi M, Nakanishi H, Mori S, et al. (2003) Three rice nicotianamine synthase genes, OsNAS1, OsNAS2, and OsNAS3 are expressed in

cells involved in long-distance transport of iron and differentially regulated by iron. Plant J 36: 366-381.

4. Song ZZ, Yang SY, Zuo J, Su YH (2014) Over-expression of ApKUP3 enhances potassium nutrition and drought tolerance in transgenic rice. Biologia Plantarum 58: 649-658.
5. Udomchalothorn T, Plaimas K, Comai L, Buaboocha T, Chadchawan S (2014) Molecular Karyotyping and Exome Analysis of Salt-Tolerant Rice Mutant from Somaclonal Variation. Plant Genome 7.
6. Ribeiro CW, Carvalho FEL, Rosa SB, Alves-Ferreira M, Andrade CMB, et al. (2012) Modulation of genes related to specific metabolic pathways in response to cytosolic ascorbate peroxidase knockdown in rice plants. Plant Biol 14: 944-955.
7. Pandit A, Rai V, Sharma TR, Sharma PC, Singh NK (2011) Differentially expressed genes in sensitive and tolerant rice varieties in response to salt-stress. J Plant Biochem Biotechnol 20: 149-154.
8. Tuteja N, Tarique M, Trivedi DK, Sahoo RK, Tuteja R (2015) Stress-induced Oryza sativa BAT1 dual helicase exhibits unique bipolar translocation. Protoplasma.
9. Walia H, Wilson C, Ismail AM, Close TJ, Cui XP (2009) Comparing genomic expression patterns across plant species reveals highly diverged transcriptional dynamics in response to salt stress. BMC Genomics 10.
10. Campos-Soriano L, Garcia-Martinez J, Segundo BS (2012) The arbuscular mycorrhizal symbiosis promotes the systemic induction of regulatory defence-related genes in rice leaves and confers resistance to pathogen infection. Mol Plant Pathol 13: 579-592.
