# Supplementary material for: In silico insights on diverse interacting partners and phosphorylation sites of respiratory burst oxidase homolog (Rbohs) gene families from Arabidopsis and rice
Source: BMC Plant Biol. 2018 Aug 10;18:161. doi: 10.1186/s12870-018-1378-2 (PMC6086027; doi:10.1186/s12870-018-1378-2)
Supplement: Supplementary file 12 — Mapping of phosphorylation sites on multiple sequence alignment of AtRboh and OsRboh protein sequences. 19 Rboh sequences were aligned with Clustal Omega (http://www.ebi.ac.uk/Tools/msa/clustalo). Gaps were indicated with dashes in the sequences. CDPK-binding motifs, two EF-hand-likes, two EF- hands, six ransmembrane spanning domains (TMD I-VI), two FAD-binding and four NADPH-binding sites were shown in black boxes. Reported (also retrieved in prediction) S/T/Y sites involved in phosphorylation were highlighted in 654 green while predicted sites were indicated in cyan. (PDF 62 kb) [file 12870_2018_1378_MOESM12_ESM.pdf]

```
OsRbohA_Q0JJJ9      ------------------------------------------------------------
OsRbohB_Q5ZAJ0      -----------M--ADLEAGMVAAAT------------------------------DQGN 17
OsRbohC_Q65XC8      -----MRAGIGSG----------------------SGGGTTPVRPRWGSGVTT----PR- 28
OsRbohD_Q0DHH6      ------------------------------------------------------------
OsRbohE_Q8S1T0      ------------------------------------------------------------
OsRbohF_Q0J595      -MWTPSRGSASGRRATGHRRIADYLADDRTE--A--STENGSFNTAYSDELFAPTSSSAG 55
OsRbohG_Q69LJ7      -MWTPSRGSNAAR-RSGHRRIADYLADDQTT--NTDTSDNESYTTAYGDEFFAAAAA-AA 55
OsRbohH_Q2QP56      -MASREESGNG--------------------------------------------G---- 11
OsRbohI_Q2R351      MHHTRAGAADGA--GGGGEDIVEAGAEAPPPQRERLVP--------HSGPLSKRS-GMRK 49
AtRbohA_O81209      -MMNR-----------------------SEMQKLGFEHVRY----YTESP---------- 22
AtRbohB_Q9SBI0      ---------------------------------------------------------MRE 3
AtRbohC_O81210      ------------------------------MSRVSFEVSGG----YHSDAEAGNSGPM-- 24
AtRbohD_Q9FIJ0      MKMRRGNSSND-----HELGILRGANSDTNSDTESIASDRG----AFSGPLGRPKRASKK 51
AtRbohE_O81211      MKLSPLSFSTSSS----------------------FSHADG---IDDGVELIS----SP- 30
AtRbohF_O48538      -----------------------------------MKPFSKNDRRRWSFDSVS----AGK 21
AtRbohG_Q9SW17      ------------------------------MQRVSFEVKDT----EA----EKSSSEI-- 20
AtRbohH_Q9FJD6      ------------------------------------------------------------
AtRbohI_Q9SUT8      ---------MSMS----------------------FSG--GTHNDRWGSDLAS----AGE 23
AtRbohJ_Q9LZU9      ------------------------------------------------------------


OsRbohA_Q0JJJ9      ------------------------------------------------------------
OsRbohB_Q5ZAJ0      STRSQDDA-ATLIPNSGNLGSSNRSTKTARFKDDDELVEITLDVQRDS-VAIQE-VRGVD 74
OsRbohC_Q65XC8      -SLSTGS---------------SPRGSDRSSDDGEELVEVTLDLQEDDTIVLRS-VEPAA 71
OsRbohD_Q0DHH6      ------------------------------------------------------------
OsRbohE_Q8S1T0      ------------------------------------------------------------
OsRbohF_Q0J595      GDGVGGMLPA-FLAD------------------QSDLVEVMLELDEESMVV-RS-VTPTT 94
OsRbohG_Q69LJ7      GSGGGGMLPA-FLAD------------------QGDLVEVMLELDEESMVV-RS-VTPTS 94
OsRbohH_Q2QP56      ---GGGATPAADYRSSD---SRSSSRRSTRFKEDNEYVEITLDVKGDDTVAIQS-IRN-- 62
OsRbohI_Q2R351      SARFAESV-SAPLSAPSGVAAARRAAAAANDDDEEDYVEITLDVRDDS-VAVHS-VKPAG 106
AtRbohA_O81209      -----------YNRGES---SANVATTSNYYGEDEPYVEITLDIHDDS-VSVYG-LKSPN 66
AtRbohB_Q9SBI0      E-----EM---ESSSEGETNKISRCKATGSDNPDEDYVEITLEVRDET-INTMK-AKAT- 52
AtRbohC_O81210      ---SGGQL-PPIYKKPG---NSRFTAENSQRTRTAPYVDLTVDVQDDT-VSVHS-LKMEG 75
AtRbohD_Q9FIJ0      NARFADDL-P-----------KRSNSVAGGRGDDDEYVEITLDIRDDS-VAVHS-VQQAA 97
AtRbohE_O81211      -FAGGAMLPV-FLNDL----SRNSGESGSGSSWERELVEVTLELDVGDDSILVCGMSEAA 84
AtRbohF_O48538      TAVGSAS---------------TSPGTEYSINGDQEFVEVTIDLQDDDTIVLRS-VEPAT 65
AtRbohG_Q9SW17      ---LSGSL-PSTYRNPA---MENV-----------------------------G-NAVDD 43
AtRbohH_Q9FJD6      --------------------MKSNTPTEDSTKWMLESVEI-------------------- 20
AtRbohI_Q9SUT8      FTQSFPSLPA-----------TYSPSPSSSSSSGEELLEVTIEFPSGVIINIDS-VTGTG 71
AtRbohJ_Q9LZU9      --------------MK----NNKKVGTEDSTKWMLESVEI-------------------- 22

                                                                 CDPK-I
OsRbohA_Q0JJJ9      ------------------------------------------------------------
OsRbohB_Q5ZAJ0      EGGSGHGTGFDGL-PLVSPSSKS------G--------------------------KLTS 101
OsRbohC_Q65XC8      GGAAVASSSGASPSAVAPPRRAEPPGG-VASR-SRSPAMRRTSSHRLLQFSQELKAEAMA 129
OsRbohD_Q0DHH6      ---MAGD-------------YVDVPLGGGGQSTL-------------------------- 18
OsRbohE_Q8S1T0      ---MASPYDHQSPHAQHPSGLPRPPGAGAGAAAG-------------------------- 31
OsRbohF_Q0J595      G-ALYGPTSLAGG------GAAHTPPGSGRSL-SRCS----S------------------ 124
OsRbohG_Q69LJ7      A-TLYGGGGGQMPQPLPPPLRTPEGGGGARSL-SRCS----S------------------ 130
OsRbohH_Q2QP56      ------GADMPEV-ALLARGLAQ-----QPP-P--------SAAPGPG--------GLSS 93
OsRbohI_Q2R351      GGGE--DSDV----TLLART-LE------KR--SS-----SFGHSVIR--------NASS 138
AtRbohA_O81209      HRGA--GSNYEDQ-SLLRQGRSG------RSN-SV-----LK--------------RL-- 95
AtRbohB_Q9SBI0      ---------------------LR------SVL-S-------------------------G 59
AtRbohC_O81210      GSSV--EES-PEL-TLLKRNRLE------KKT-TV-----VK--------------RL-- 103
AtRbohD_Q9FIJ0      GGGG--HLEDPEL-ALLTKKTLE------SSL-NN-----TTSLSFFR--------STSS 134
AtRbohE_O81211      S---------------------------VDSR-ARSVDLVTA------RLSRNLS-NAST 109
AtRbohF_O48538      AINVIGDISDDNTGIMT-----------PVSI-SRSPTMKRTSSNRFRQFSQELKAEAVA 113
AtRbohG_Q9SW17      GSSV--KNN-PKL-DMQKQNGLV------KWF-K-------------------------- 66
AtRbohH_Q9FJD6      --DSMGESSSKEPEINLNKN-------EGGLKKNASRNLGVG------------------ 53
AtRbohI_Q9SUT8      T-----DISGTDLEITSC---S--DSG-SG---SRSLSLGWS------ASSERLTA--GT 109
AtRbohJ_Q9LZU9      --DPKGDSSVKQPESTINSNNPESSGAGGGILKNVSKNLAVG------------------ 62
```

**CDPK-II**

```
OsRbohA_Q0JJJ9    -----------------------------------------------------------
OsRbohB_Q5ZAJ0    KLRQVTNGLKMKSSSRKAP-----------------------SPQAQQSAKRVRKRLDRT 138
OsRbohC_Q65XC8    RARQFSQ-DLTKRF----TRT---------QSTTTAPPGIESALAARAERRQRAQLDRT 174
OsRbohD_Q0DHH6    ----PPVAPLKKQP----SRLASGMK---RLAS----------------MVPDTMKLKRT 51
OsRbohE_Q8S1T0    ----GFARGLMKQP----SRLASGVR---QFASRVSMKVP-E---GVGGMRPGGGRMTRM 76
OsRbohF_Q0J595    ----TSSRIRKKFAWLRSPSPAP----APRAPTPSEPPPPREAAMAARERRRIQARLNRS 176
OsRbohG_Q69LJ7    ----TSSRIRKKFAWLRSPSPSP----SPRPPTPAEL--QREAAMAARERRRIQARLNRS 180
OsRbohH_Q2QP56    RLKAVRTELRRIASWKFPSGVLSG--------------GGGGDAPGNGNDRRPRLDRS 138
OsRbohI_Q2R351    RIKQVSQELRRLASV-----------------------------NRRGGGGGGPRFDRS 168
AtRbohA_O81209    -ASSVSTGITRVASSVS-----------------------SSSARKPPRPQLAKLRRS 129
AtRbohB_Q9SBI0    RLKTMV--------------------------------------KSLSFASRRLDRS 78
AtRbohC_O81210    -AS-VSHELKRLTSVSG------------------------GIGGRKPPR--PAKLDRT 134
AtRbohD_Q9FIJ0    RIKNASRELRRVFS-------------------------------RRPSPAVRRFDRT 161
AtRbohE_O81211    RIRQKLGKLLRSESWKTTTSST----AGERDRDLERQTAVTLGILTARDKRKEDAKLQRS 165
AtRbohF_O48538    KAKQLSQ-ELKRFSWSRSFSGNLTTTSTAANQSGGAGGGLVNSALEARALRKQRAQLDRT 172
AtRbohG_Q9SW17    --------KCLTMVSG-----------------------------ESK--APRLDRS 84
AtRbohH_Q9FJD6    ----SIIRTLSVSNWRKSGNLGSPST---RKSGN--LGPPTN---AV-PKKTGPQRVERT 100
AtRbohI_Q9SUT8    NSKQQIQ-KISRRGYGYSSRS-------APEPVVPHRGEITDSVNLPRALSQRPTRPNRD 161
AtRbohJ_Q9LZU9    ----SIIRSMSVNKWRKSGNLGSPST---RKSGN--LGPPLP---VSQVKRPGPQRVERT 110
```

**EF-hand-like I**

```
OsRbohA_Q0JJJ9    ---------------------------------------------AR--DGYLSRSDFA 12
OsRbohB_Q5ZAJ0    KS-SAAVALKGLQFVTAKV-GN-----------DGWAAVEKRFNQLQV--DGVLLRSRFG 183
OsRbohC_Q65XC8    KS-GAQRAIRGLRFISGPNKAS-----------NAWIEVQANFDRLAR--DGYLSRDDFP 220
OsRbohD_Q0DHH6    HS-SAQPALRGLRFLDKTSAG-----------KDGWKNVEKRFDEMSA--DGRLPQESFA 97
OsRbohE_Q8S1T0    QS-SAQVGLRGLRFLDKTSGG-----------KEGWKSVERRFDEMNR--NGRLPKESFG 122
OsRbohF_Q0J595    RS-GARRALKGLRFISRTTGSA---------EAAELWTRVEHRFNALSR--DGLLSRDNFG 225
OsRbohG_Q69LJ7    ST-GAKRALKGLRFISRTTGTV---------QAAELWRRVEDRFNALAR--DGLLSRDDFG 229
OsRbohH_Q2QP56    MT-GAARALRGLQFLNSSA-VT-----------NGWPEVEKRFERLAV--DGFLLRSRFG 183
OsRbohI_Q2R351    KS-AAAHALKGLKFISRAD-GG-----------AGWPAVEKRFDDLAK--DGLLPRSKFG 213
AtRbohA_O81209    KS-RAELALKGLKFITKTD-GV-----------TGWPEVEKRFYVMTMTNNGLLHRSRFG 176
AtRbohB_Q9SBI0    KSFGAMFALRGLRFIAKNDAVG-----------RGWDEVAMRFDKLAV--EGKLPKSKFG 125
AtRbohC_O81210    KS-AASQALKGLKFISKTD-GG-----------AGWSAVEKRFNQITATTGGLLLRTKFG 181
AtRbohD_Q9FIJ0    SS-AAIHALKGLKFIAT---KT-----------AAWPAVDQRFDKLSADSNGLLLSAKFW 206
AtRbohE_O81211    TS-SAQRALKGLQFINKTTRGNSCVCDWDCDCDQMWKKVEKRFESLSK--NGLLARDDFG 222
AtRbohF_O48538    RS-SAQRALRGLRFISNKQKNV-----------DGWNDVQSNFEKFEK--NGYIYRSDFA 218
AtRbohG_Q9SW17    KS-TAGQALKGLKIISKTD-GN-----------AAWTVVEKRYLKITANTDGLLLRSKFG 131
AtRbohH_Q9FJD6    TS-SAARGLQSLRFLDRTVTGR---------ERDAWRSIENRFNQFSV--DGKLPKEKFG 148
AtRbohI_Q9SUT8    GS-GTERAIHGLKFISSKENGI-----------VDWNDVQNNFAHLSK--DGYLFKSDFA 207
AtRbohJ_Q9LZU9    TS-SAARGLQSLRFLDRTVTGR---------ERDSWRSIENRFNQFAV--DGRLPKDKFG 158
```

**EF-hand-like II**

```
OsRbohA_Q0JJJ9    ECIGMT-------------------------------ESKEFALELFDTLSRRRQMKVD- 40
OsRbohB_Q5ZAJ0    KCIGMD------------------------------G-SDEFAVQMFDSLARKRGIVKQ- 211
OsRbohC_Q65XC8    QCIGMT-------------------------------ESKEFAMELFDTLSRRRQMQVD- 248
OsRbohD_Q0DHH6    KCIGMA-------------------------------DSKEFASEVFVALARRRSIKPED 126
OsRbohE_Q8S1T0    KCIGMG-------------------------------DSKEFAGELFVALARRRNLEPED 151
OsRbohF_Q0J595    DCIGKQAKPSSMSMARRARARSRDDTAYGAGIGAGMEDSKEFAVGIFDALARRRRQELE- 284
OsRbohG_Q69LJ7    ECIG-----------------------------MVDSKEFAVGIFDALARRRRQNLE- 257
OsRbohH_Q2QP56    QCIGMV------------------------------G-SEEFAVQIFDSLARRRGITAQ- 211
OsRbohI_Q2R351    QCIGMK------------------------------E--LEFAGELFDALARRRNISGD- 240
AtRbohA_O81209    ECIGMK------------------------------S--TEFALALFDALARRENVSGD- 203
AtRbohB_Q9SBI0    HCIGMV------------------------------E-SSEFVNELFEALVRRRGTTSS- 153
AtRbohC_O81210    ECIGMT------------------------------S--KDFALELFDALARRRNITGE- 208
AtRbohD_Q9FIJ0    ECLGMN------------------------------KESKDFADQLFRALARRNNVSGD- 235
AtRbohE_O81211    ECVGMV-------------------------------DSKDFAVSVFDALARRRRQKLE- 250
AtRbohF_O48538    QCIGMK-------------------------------DSKEFALELFDALSRRRRLKVE- 246
AtRbohG_Q9SW17    ECIGMN------------------------------S--KEFALELFDALARKSHLKGD- 158
AtRbohH_Q9FJD6    VCIGMG-------------------------------DTMEFAAEVYEALGRRRQIETEN 177
AtRbohI_Q9SUT8    HCIGLE-----------------------------NENSKEFADELFDALCRRRRIMVD- 237
AtRbohJ_Q9LZU9    VCIGMG-------------------------------DTLEFAAKVYEALGRRRQIKTEN 187
```

**EF-hand-like II** **EF-hand I**

```
OsRbohA_Q0JJJ9      TINKDELREIWQQITDNSFDSRLQIFFEMVDKNADGRITEAEVKEIIMLSASANKLSRLK 100
OsRbohB_Q5ZAJ0      VLTKDELKDFYEQLTDQGFDNRLRTFFDMVDKNADGRLTAEEVKEIIALSASANKLSKIK 271
OsRbohC_Q65XC8      KINKEELREIWQQITDNSFDSRLQIFFDMVDKNADGHITEAEVKEIIMLSASANKLSRLK 308
OsRbohD_Q0DHH6      GITKEQLKEFWEELTDQNFDSRLRIFFDMCDKNGDGQLTEDEVKEVIVLSAAANKLAKLK 186
OsRbohE_Q8S1T0      GITKEQLKEFWEEMTDQNFDSRLRIFFDMCDKNGDGMLTEDEVKEVIILSASANKLAKLK 211
OsRbohF_Q0J595      RISKEELYDFWLI---------------------------------VLSASANKLSKLK 310
OsRbohG_Q69LJ7      RITREELYDFWLQISDQSFDARLQIFFDMVDTNVDGRITREEVQELIVLSASANKLAKLK 317
OsRbohH_Q2QP56      LLTKDQLREFWEQLSDPGFDAKLQTFFDMVDKNADGQITEEELKEVLTLTASANKLSKIL 271
OsRbohI_Q2R351      SISKAELLEFWDQISDTSFDSRLQTFFDMVDKNADGRITEEEVKEIITLSASANKLSKVQ 300
AtRbohA_O81209      SININELKEFWKQITDQDFDSRLRTFFAMVDKDSDGRLNEAEVREIITLSASANELDNIR 263
AtRbohB_Q9SBI0      SITKTELFEFWEQITGNSFDDRLQIFFDMVDKNLDGRITGDEVKEIIALSASANKLSKIK 213
AtRbohC_O81210      VIDGDQLKEFWEQINDQSFDSRLKTFFDMVDKDADGRLTEDEVREIISLSASANNLSTIQ 268
AtRbohD_Q9FIJ0      AITKEQLRIFWEQISDESFDAKLQVFFDMVDKDEDGRVTEEEVAEIISLSASANKLSNIQ 295
AtRbohE_O81211      KITKDELHDFWLQISDQSFDARLQIFFDMADSNEDGKITREEIKELLMLSASANKLAKLK 310
AtRbohF_O48538      KINHDELYEYWSQINDESFDSRLQIFFDIVDKNEDGRITEEEVKEIIMLSASANKLSRLK 306
AtRbohG_Q9SW17      VITETELKKFWEQINDKSFDSRLITFFDLMDKDSDGRLTEDEVREIIKLSSSANHLSCIQ 218
AtRbohH_Q9FJD6      GIDKEQLKLFWEDMIKKDLDCRLQIFFDMCDKNGDGKLTEEEVKEVIVLSASANRLGNLK 237
AtRbohI_Q9SUT8      KINLQELYEFWYQITDESFDSRLQIFFNMV-KNGDGRITENEVKEIIILSASANNLSRLR 296
AtRbohJ_Q9LZU9      GIDKEQLKLFWEDMIKKDLDCRLQIFFDMCDKDGDGKLTEEEVKEVIVLSASANRLVNLK 247
```

**EF-hand II**

```
OsRbohA_Q0JJJ9      EQAEEYAALIMEELDPEGL--GYIE----------------------LWQLETLLLQKD 135
OsRbohB_Q5ZAJ0      ERADEYTALIMEELDPTNL--GYIE----------------------MEDLEALLLQSP 306
OsRbohC_Q65XC8      EQAEEYAALIMEELDPEQL--GYIE----------------------LWQLETLLLQKD 343
OsRbohD_Q0DHH6      SHAATYASLIMEELDPDHR--GYIE----------------------IWQLETLLRGMV 221
OsRbohE_Q8S1T0      GHAATYASLIMEELDPDDR--GYIE----------------------IWQLETLLRGMV 246
OsRbohF_Q0J595      EQAEEYASLIMEELDPEDL--GYIE----------------------LWQLEALLLQRD 345
OsRbohG_Q69LJ7      EQAEEYASLIMEELDPENL--GYIE----------------------LWQLEALLLQRD 352
OsRbohH_Q2QP56      ERVDEYTALIMEELDPDQL--GYID----------------------ISNLESLLLLPP 306
OsRbohI_Q2R351      EQSEEYARLIMEELDPSNL--GYIE----------------------LYNLEMLLLQAP 335
AtRbohA_O81209      RQADEYAALIMEELDPYHY--GYIM----------------------IENLEILLLQAP 298
AtRbohB_Q9SBI0      ENVDEYAALIMEELDRDNL--GYIE----------------------LHNLETLLLQVP 248
AtRbohC_O81210      KRADEYAALIMEELDPDNI--GYIM----------------------LESLETLLLQAA 303
AtRbohD_Q9FIJ0      KQAKEYAALIMEELDPDNA--GFIM----------------------IENLEMLLLQAP 330
AtRbohE_O81211      EQAEEYASLIMEELDPENF--GYIE----------------------LWQLETLLLQRD 345
AtRbohF_O48538      EQAEEYAALIMEELDPERL--GYIE----------------------LWQLETLLLQKD 341
AtRbohG_Q9SW17      NKADEYAAMIMEELDPDHM--GYIM----------------------MESLKKLLLQAE 253
AtRbohH_Q9FJD6      KNAAAYASLIMEELDPDHK--GYIE----------------------MWQLEILLTGMV 272
AtRbohI_Q9SUT8      ERAEEYAALIMEELAPDGLYSQYIE----------------------LKDLEILLLEKD 333
AtRbohJ_Q9LZU9      KNAASYASLIMEELDPNEQ--GYIE----------------------MWQLEVLLTGIV 282
```

```
OsRbohA_Q0JJJ9      TYMNYSQALSYTSQAL-SQNLA-------------------------------GL-RKK 161
OsRbohB_Q5ZAJ0      SEAAARS-TTTHSSKL-SKALS----------------------------MKLASNKEM 335
OsRbohC_Q65XC8      TYVNYSQALSYTSQAL-SQNLA-------------------------------GL-RKR 369
OsRbohD_Q0DHH6      TAQGPPEKVKLASASL-----AR----------------------------TMVPSSHR 247
OsRbohE_Q8S1T0      SAQAAPEKMKRTTSSL-----AR----------------------------TMIPSRYR 272
OsRbohF_Q0J595      AYMNYSRPLSSGSTAQWSQNLGGGGGGQQG----------GQGQGQGQSEGRRNDWRRR 394
OsRbohG_Q69LJ7      SYMNYSRPLSTASGAQWSQNLGGAAVAAGAAAATGGGAHAAVAARGGQQQQQQEGRRGG 412
OsRbohH_Q2QP56      SQAPSKL--VTHSSNI-SQLIS----------------------------QKLVPTHDR 334
OsRbohI_Q2R351      SQSVRI--GTTNSRNL-SQMLS----------------------------QNLRPTAEP 363
AtRbohA_O81209      MQD----VRDGESKKL-SKMLS----------------------------QNLMVPQSR 324
AtRbohB_Q9SBI0      SQSNNSP-SSANKRAL-NKMLS----------------------------QKLIPTKDR 277
AtRbohC_O81210      TQSVITS--TGERKNL-SHMMS----------------------------QRLKPTFNR 331
AtRbohD_Q9FIJ0      NQSVRM--GD--SRIL-SQMLS----------------------------QKLRPAKES 356
AtRbohE_O81211      AYMNYSRPLSTTSGGV-STPRR--------------------------------NLIRPR 372
AtRbohF_O48538      TYLNYSQALSYTSQAL-SQNLQ-------------------------------GL-RGK 367
AtRbohG_Q9SW17      TKSVSTDINSEERKEL-SDMLT----------------------------ESLKPTRDP 283
AtRbohH_Q9FJD6      TNAD-TEK-MKKSQTL-----TR----------------------------AMIPERYR 296
AtRbohI_Q9SUT8      ISHSYSLPFSQTSRAL-SQNLK-------------------------------DR-R-- 357
AtRbohJ_Q9LZU9      SNAD-SHKVVRKSQQL-----TR----------------------------AMIPKRYR 307
```

```
                                                 TMD-I               A-loop

OsRbohA_Q0JJJ9      SSIRKISTSLSYYFEDNWKRLWVLALWIGIMAGLFTWKFMQYRNRYVFDVMGYCVTTAKG 221
OsRbohB_Q5ZAJ0      SPVRHYWQQFMYFLEENWKRSWVMTLWISICIALFIWKFIQYRNRAVFGIMGYCVTTAKG 395
OsRbohC_Q65XC8      SPIRKISTKLSYYLEDNWKRLWVLALWIGIMAGLFIWKFIQYRHRYVFNVMGYCVTTAKG 429
OsRbohD_Q0DHH6      SPMQRRFNKTVDFIHENWKRIWVLSLWAILNIALFMYKFVQYSRRDAFQVMGYCVCIAKG 307
OsRbohE_Q8S1T0      SPLKRHVSRTVDFVHENWKRIWLVALWLAVNVGLFAYKFEQYERRAAFQVMGHCVCVAKG 332
OsRbohF_Q0J595      WSPRRAAARAQVAAEENWRRAWVLALWFAAMAGLFAWKFVQYRRTPAFRVMGYCLPTAKG 454
OsRbohG_Q69LJ7      WGVRKAAARVRVAAEENWRRAWVVALWFAAMASLFVWKFVQYRRTPAFRVMGYCLPTAKG 472
OsRbohH_Q2QP56      NPLRRGLRRLSYFMEDNWKRVWVMALWLAINAGLFTWKFMAYKRHPTFDVMGYCVCVAKG 394
OsRbohI_Q2R351      NPLRRWWRRASYFLEDNWRRVWVLLLWLAICAGLFTYKFIQYRHRAVFHVMGYCVCVAKG 423
AtRbohA_O81209      NLGARFCRGMKYFLFDNWKRVWVMALWIGAMAGLFTWKFMEYRKRSAYEVMGVCVCIAKG 384
AtRbohB_Q9SBI0      NPVKRFAMNISYFFLENWKRIWVLTLWISICITLFTWKFLQYKRKTVFEVMGYCVTVAKG 337
AtRbohC_O81210      NPLKRWYRGLRFFLLDNWQRCWVIVLWFIVMAILFTYKYIQYRRSPVYPVMGDCVCMAKG 391
AtRbohD_Q9FIJ0      NPLLRWSEKIKYFILDNWQRLWIMMLWLGICGGLFTYKFIQYKNKAAYGVMGYCVCVAKG 416
AtRbohE_O81211      HVVQKCRKKLQCLILDNWQRSWVLLVWVMLMAILFVWKFLEYREKAAFKVMGYCLTTAKG 432
AtRbohF_O48538      SRIHRMSSDFVYIMQENWKRIWVLSLWIMIMIGLFLWKFFQYKQKDAFHVMGYCLLTAKG 427
AtRbohG_Q9SW17      NHLRRWYCQLRFFVLDSWQRVWVIALWLTIMAILFAYKYIQYKNRAVYEVLGPCVCLAKG 343
AtRbohH_Q9FJD6      TPMSKYVSVTAELMHENWKKLWVLALWAIINVYLFMWKYEEFMRNPLYNITGRCVCAAKG 356
AtRbohI_Q9SUT8      ---WRMSRNLLYSLQDNWKRIWVLTLWFVIMAWLFMWKCYQYKHKDAFHVMGYCLVMAKG 414
AtRbohJ_Q9LZU9      TPTSKYVVVTAELMYEHWKKIWVVTLWLAVNVVLFMWKYEEFTTSPLYNITGRCLCAAKG 367


                             TMD-II            B-loop              TMD-III

OsRbohA_Q0JJJ9      AAETLKLNMAIILLPVCRNTITWLRSTR--AARALPFDDNINFHKT-------------- 265
OsRbohB_Q5ZAJ0      AAETLKFNMALVLLPVCRNTITWIRSKTQ-VGAVVPFNDNINFHKV-------------- 440
OsRbohC_Q65XC8      AAETLKLNMAIILLPVCRNTITWLRNTR--AARALPFDDNINFHKT-------------- 473
OsRbohD_Q0DHH6      AAETLKLNMAVILLPVCRNTLTRLRSTA--LSKVVPFDDNINFHKV-------------- 351
OsRbohE_Q8S1T0      AAEVLKLNMALILLPVCRNTLTTLRSTA--LSHVIPFDDNINFHKV-------------- 376
OsRbohF_Q0J595      AAETLKLNMALVLLPVCRNTLTWLRSSW--ARFFVPFDDSITFHKVESSSGFKWAPPGRS 512
OsRbohG_Q69LJ7      AAETLKLNMALVLLPVCRNTLTWLRSSW--ARFFVPFDDNITFHKM-------------- 516
OsRbohH_Q2QP56      GAETTKFNMALILLPVCRNTITWLRSRTK-LGAVIPFNDNINFHKV-------------- 439
OsRbohI_Q2R351      GAETLKFNMALILLPVCRNTVTWIRNRAA-VARVVPFDDNLNFHKV-------------- 468
AtRbohA_O81209      AAETLKLNMAMILLPVCRNTITWLRTKTK-LSAIVPFDDSLNFHKV-------------- 429
AtRbohB_Q9SBI0      SAETLKFNMALILLPVCRNTITWLRTKSKLIGSVVPFDDNINFHKV-------------- 383
AtRbohC_O81210      AAETVKLNMALILLPVCRNTITWLRNKTR-LGRVVPFDDNLNFHKV-------------- 436
AtRbohD_Q9FIJ0      GAETLKFNMALILLPVCRNTITWLRNKTK-LGTVVPFDDSLNFHKV-------------- 461
AtRbohE_O81211      AAETLKLNMALVLLPVCRNTLTWLRSTR--ARACVPFDDNINFHKI-------------- 476
AtRbohF_O48538      AAETLKFNMALILFPVCRNTITWLRSTR--LSYFVPFDDNINFHKT-------------- 471
AtRbohG_Q9SW17      AAETLKLNMALILLPVCRNTITWLRNKTR-LGVFVPFDDNLNFHKV-------------- 388
AtRbohH_Q9FJD6      AAETLKLNMALILVPVCRKTLTILRSTF--LNRVVPFDDNINFHKV-------------- 400
AtRbohI_Q9SUT8      AAETLKFNMALILLPVCRNTITYLRSTA--LSHSVPFDDCINFHKT-------------- 458
AtRbohJ_Q9LZU9      TAEILKLNMALILVPVLRRTLTFLRSTF--LNHLIPFDDNINFHKL-------------- 411


OsRbohA_Q0JJJ9      --------------------------------------------IAAAIVVGIILHAGNH 281
OsRbohB_Q5ZAJ0      --------------------------------------------IAAGVAVGVALHAGAH 456
OsRbohC_Q65XC8      --------------------------------------------IAAAIVVGVILHGGLH 489
OsRbohD_Q0DHH6      --------------------------------------------IALTIAIGAATHTLAH 367
OsRbohE_Q8S1T0      --------------------------------------------IAATIAAATAVHTLAH 392
OsRbohF_Q0J595      NQCSVAGEPAGTEDPRPGRLRVKAPVIQKSPRPTGLPLSCGATIIATAIALGICTHAGTH 572
OsRbohG_Q69LJ7      --------------------------------------------IATAIVVGITLHAGNH 532
OsRbohH_Q2QP56      --------------------------------------------VAGGVVVGVALHGVTH 455
OsRbohI_Q2R351      --------------------------------------------IAVGITVGAGLHVISH 484
AtRbohA_O81209      --------------------------------------------IAIGISVGVGIHATSH 445
AtRbohB_Q9SBI0      --------------------------------------------VAFGIAVGIGLHAISH 399
AtRbohC_O81210      --------------------------------------------IAVGIIVGVTMHAGAH 452
AtRbohD_Q9FIJ0      --------------------------------------------IASGIVVGVLLHAGAH 477
AtRbohE_O81211      --------------------------------------------IACAIAIGILVHAGTH 492
AtRbohF_O48538      --------------------------------------------IAGAIVVAVILHIGDH 487
AtRbohG_Q9SW17      --------------------------------------------IAVGIAIGVAIHSVSH 404
AtRbohH_Q9FJD6      --------------------------------------------IAYMIAFQALLHTALH 416
AtRbohI_Q9SUT8      --------------------------------------------ISVAIISAMLLHATSH 474
AtRbohJ_Q9LZU9      --------------------------------------------IAVAIAVISLLHTALH 427
```

```
                                   C-loop                          TMD-IV

OsRbohA_Q0JJJ9     LVCDFPRLIKSSDEKYAP-LGQYFGEIK-PTYFTLVKGVEGITGVIMVVCMIIAFTLATR 339
OsRbohB_Q5ZAJ0     LTCDFPRLLHASDAQYEL-MKPFFGEKRPPNYWWFVKGTEGWTGVVMVVLMAIAFTLAQP 515
OsRbohC_Q65XC8     LVCDFPRLIGSSEEKYAP-LGKYFGETK-PTYLTLVKGVEGITGVIMLVCMIIAFTLATR 547
OsRbohD_Q0DHH6     VTCDFPRLVSCPRDKFEATLGPYFNYVQ-PTYSSLVASTPGWTGILMILIMSFSFTLATH 426
OsRbohE_Q8S1T0     VTCDFPRLINCPSDKFMATLGPNFGYRQ-PTYADLLESAPGVTGILMIIIMSFSFTLATH 451
OsRbohF_Q0J595     LACDFPRLIGSSREEYELLLSGFFGASR-PTYRGLLAGVEGVTGIVMVVLMVVSFTLATR 631
OsRbohG_Q69LJ7     LACDFPRLIASGPEEYRLVA-DAFGPEK-PTYVGLLSGVEGITGVAMVVLMTVSFTLATH 590
OsRbohH_Q2QP56     LTCDFPRLLHASDAAYEP-MKKYFGQTRIPDYWWFVRGVEGITGVIMVVLMAIAYTLAHP 514
OsRbohI_Q2R351     LTCDFPRLLHATDAEYEP-MKRFFGDTRPPNYWWFVKGTEGWTGLVMLVLMAVAFTLATP 543
AtRbohA_O81209     LACDFPRLIAADEDQYEP-MEKYFGP-QTKRYLDFVQSVEGVTGIGMVVLMTIAFTLATT 503
AtRbohB_Q9SBI0     LACDFPRLLHAKNVEFEP-MKKFFGDERPENYGWFMKGTDGWTGVTMVVLMLVAYVLAQS 458
AtRbohC_O81210     LACDFPRLLHATPEAYRP-LRQFFGDEQPKSYWHFVNSVEGITGLVMVLLMAIAFTLATP 511
AtRbohD_Q9FIJ0     LTCDFPRLIAADEDTYEP-MEKYFGD-QPTSYWWFVKGVEGWTGIVMVVLMAIAFTLATP 535
AtRbohE_O81211     LACDFPRIINSSPEQFVL-IASAFNGTK-PTFKDLMTGAEGITGISMVILTTIAFTLAST 550
AtRbohF_O48538     LACDFPRIVRATEYDYNRYLFHYFQTKQ-PTYFDLVKGPEGITGILMVILMIISFTLATR 546
AtRbohG_Q9SW17     LACDFPLLIAATPAEYMP-LGKFFGEEQPKRYLHFVKSTEGITGLVMVFLMVIAFTLAMP 463
AtRbohH_Q9FJD6     IFCNYPRLSSCSYDVFLTYAGAALGNTQ-PSYLGLMLTSVSITGVLMIFFMGFSFTLAMH 475
AtRbohI_Q9SUT8     LACDFPRILASTDTDYKRYLVKYFGVTR-PTYFGLVNTPVGITGIIMVAFMLIAFTLASR 533
AtRbohJ_Q9LZU9     MLCNYPRLSSCPYNFYSDYAGNLLGAKQ-PTYLGLMLTPVSVTGVLMIIFMGISFTLAMH 486

                          D-loop                                  TMD-V

OsRbohA_Q0JJJ9     WFRRS----------------LVK--LPRPFDKLTGFNAFWYSHHLFIIVYIALIVHGEC 381
OsRbohB_Q5ZAJ0     WFRRN----------------KLK--DSNPLKKMTGFNAFWFTHHLFVIVYTLLFVHGTC 557
OsRbohC_Q65XC8     WFRRS----------------LVK--LPKPFDKLTGFNAFWYSHHLFIIVYISLVIHGEW 589
OsRbohD_Q0DHH6     SFRRS----------------VVK--LPSPLHHLAGFNAFWYAHHLLVIAYILLVLHSYF 468
OsRbohE_Q8S1T0     SFRRS----------------VVK--LPSPLHHLAGFNAFWYAHHLLVLAYVLLVVHSYF 493
OsRbohF_Q0J595     PLRKRE---------------APR--LPFPLGHLAGFNAFWYSHHLLIVVYLLLLVHGWF 674
OsRbohG_Q69LJ7     PFRKGEKGGSGGGAAATVLPTVAR--LPSPFNRLAGFNAFWYSHHLLGIVYALLIAHGYF 648
OsRbohH_Q2QP56     WFRRS----------------KLS--DSNPLKRLSGFNMFWYSHHLFVIVYIAFVVHGVC 556
OsRbohI_Q2R351     WFRRG----------------RLR--LPRPLNRLTGFNAFWYSHHCFVIVYALLIVHGYY 585
AtRbohA_O81209     WFRRN----------------KLN--LPGPLKKITGFNAFWYSHHLFVIVYSLLVVHGFY 545
AtRbohB_Q9SBI0     WFRRN----------------RAN--LPKSLKRLTGFNAFWYSHHLFVIVYVLLIVHGYF 500
AtRbohC_O81210     WFRRG----------------KLN-YLPGPLKKLASFNAFWYTHHLFVIVYILLVAHGYY 554
AtRbohD_Q9FIJ0     WFRRN----------------KLN--LPNFLKKLTGFNAFWYTHHLFIIVYALLIVHGIK 577
AtRbohE_O81211     HFRRN----------------RVR--LPAPLDRLTGFNAFWYTHHLLVVVYIMLIVHGTF 592
AtRbohF_O48538     WFRRN----------------LVK--LPKPFDRLTGFNAFWYSHHLFVIVYILLILHGIF 588
AtRbohG_Q9SW17     WFRRG----------------KLEKKLPGPLKKLASFNAFWYTHHLFVIVYILLVLHGYY 507
AtRbohH_Q9FJD6     YFRRN----------------IVK--LPKPFNVLAGFNAFWYAHHLLVLAYILLIIHGYY 517
AtRbohI_Q9SUT8     RCRRN----------------LTK--LPKPFDKLTGYNAFWYSHHLLLTVYVLLVIHGVS 575
AtRbohJ_Q9LZU9     YFRRN----------------IVK--LPIPFNRLAGFNSFWYAHHLLVIAYALLIIHGYI 528

                   E-loop          TMD-VI

OsRbohA_Q0JJJ9     LYLI-HVWYRRTTWMYLSVPVCLYVGERILRFF-RSG-SYSVRLLKVAIYPGNVLTLQMS 438
OsRbohB_Q5ZAJ0     LYLS-RKWYKKTTWMYLAVPVVLYVSERILRLF-RSH--DAVGIQKVAVYPGNVLALYMS 613
OsRbohC_Q65XC8     LYLI-RIWYKRTTWMYLAVPVCLYVGERTLRFF-RSG-SYSVRLLKVAIYPGNVLTLQMS 646
OsRbohD_Q0DHH6     IFLT-KQWYNRTTWMFLAVPVLFYSCERTIRRV-RES-SYGVTVIKAAIYPGNVLSIHMN 525
OsRbohE_Q8S1T0     IFLT-REWYKKTTWMYLIVPVLFYACERTIRKV-REN-NYRVSIVKAAIYPGNVLSLHMK 550
OsRbohF_Q0J595     MFLV-TKWHQRTTWMYIAVPLMLYVGERTLRAF-RSK-AYAVKILKVCLLPGNVLTITMS 731
OsRbohG_Q69LJ7     LFLV-RRWYLKTTWMYISVPLMLYVGERMLRAL-RSN-AYAVKILKVCLLPGNVLTITMS 705
OsRbohH_Q2QP56     LYIN-RTWWKQTTWMYLAIPILLYAGERIFRAL-RSHGFTTVRIEKVAIYPGNVIAIHMT 614
OsRbohI_Q2R351     LFLT-KDWYKKTTWMYLAVPMFLYACERLTRAL-RSS-VRPVKILKVAVYPGNVLSLHFS 642
AtRbohA_O81209     VYLIIEPWYKKTTWMYLMVPVVLYLCERLIRAF-RSS-VEAVSVLKVAVLPGNVLSLHLS 603
AtRbohB_Q9SBI0     VYLS-KEWYHKTTWMYLAVPVLLYAFERLIRAF-RPG-AKAVKVLKVAVYPGNVLSLYMS 557
AtRbohC_O81210     LYLT-RDWHNKTTWMYLVVPVVLYACERLIRAF-RSS-IKAVTIRKVAVYPGNVLAIHLS 611
AtRbohD_Q9FIJ0     LYLT-KIWYQKTTWMYLAVPILLYASERLLRAF-RSS-IKPVKMIKVAVYPGNVLSLHMT 634
AtRbohE_O81211     LFFA-DKWYQKTTWMYISVPLVLYVAERSLRAC-RSK-HYSVKILKVSMLPGEVLSLIMS 649
AtRbohF_O48538     LYFA-KPWYVRTTWMYLAVPVLLYGGERTLRYF-RSG-SYSVRLLKVAIYPGNVLTLQMS 645
AtRbohG_Q9SW17     IYLN-KEWYKKTTWMYLAVPVALYAYERLIRAF-RSS-IRTVKVLKMAAYPGKVLTLQMS 564
AtRbohH_Q9FJD6     LIIE-KPWYQKTTWMYLAVPMLFYASERLFSRLLQEH-SHRVNVIKAIVYSGNVLALYVT 575
AtRbohI_Q9SUT8     LYLE-HKWYRKTVWMYLAVPVLLYVGERIFRFF-RSR-LYTVEICKVVIYPGNVVVLRMS 632
AtRbohJ_Q9LZU9     LIIE-KPWYQKTTWMYVAIPMVLYASERLFSRV-QEH-NHRVHIIKAIVYSGNVLALYMT 585
```

**FAD-I** **FAD-II**

```
OsRbohA_Q0JJJ9     KPPTFRYKSGQYMFVQCPAVSPFEWHPFSITSAPGDDYLSIHVRQLGDWTRELKRVFAAA 498
OsRbohB_Q5ZAJ0     KPPGFRYRSGQYIFIKCTAVSPYEWHPFSITSAPGDDYLSVHIRTRGDWTSRLRTVFSEA 673
OsRbohC_Q65XC8     KPPTFRYKSGQYMFVQCPAVSPFEWHPFSITSAPGDDYLSIHVRQLGDWTRELKRVFSAA 706
OsRbohD_Q0DHH6     KPSSFKYKSGMYMFVKCPDVSPFEWHPFSITSAPGDDYLSVHIRTLGDWTTELRNLFGKA 585
OsRbohE_Q8S1T0     KPPGFKYKSGMYLFVKCPDVSPFEWHPFSITSAPGDDYLSVHIRTLGDWTTELRNLFGKA 610
OsRbohF_Q0J595     KPYGFRYRSGQYIFLQCPTISPFEWHPFSITSAPGDDYISVHIQTRGDWTQELKRIFVEN 791
OsRbohG_Q69LJ7     KPYGFRYRSGQYIFLQCPTISPFEWHPFSITSAPGDDYLSVHIRTNGDWTQELKRIFVEN 765
OsRbohH_Q2QP56     KPHGFKYKSGQYIYVNCGEVSPFEWHPFTITSAPDDSYLSMHIRCRGDWTSSFRAIFSQI 674
OsRbohI_Q2R351     KPQGFKYKSGQYIFVNCAAVSPFQWHPFSITSAPQDDYVSVHIRTLGDWTRELKNVFSRV 702
AtRbohA_O81209     RPSNFRYKSGQYMYLNCSAVSTLEWHPFSITSAPGDDYLSVHIRVLGDWTKQLRSLFSEV 663
AtRbohB_Q9SBI0     KPKGFKYTSGQYIYINCSDVSPLQWHPFSITSASGDDYLSVHIRTLGDWTSQLKSLYSKV 617
AtRbohC_O81210     RPQNFKYKSGQYMFVNCAAVSPFEWHPFSITSAPQDDYLSVHIRVLGDWTRALKGVFSEV 671
AtRbohD_Q9FIJ0     KPQGFKYKSGQFMLVNCRAVSPFEWHPFSITSAPGDDYLSVHIRTLGDWTRKLRTVFSEV 694
AtRbohE_O81211     KPPGFKYKSGQYIFLQCPTISRFEWHPFSITSAPGDDQLSVHIRTLGDWTEELRRVLTVG 709
AtRbohF_O48538     KPTQFRYKSGQYMFVQCPAVSPFEWHPFSITSAPEDDYISIHIRQLGDWTQELKRVFSEV 705
AtRbohG_Q9SW17     KPTNFKYMSGQYMFVNCPAVSPFEWHPFSITSTPQDDYLSVHIKALGDWTEAIQGVFSEV 624
AtRbohH_Q9FJD6     KPPGFKYKSGMYMFVKCPDLSKFEWHPFSITSAPGDDYLSVHIRALGDWTTELRSRFAKT 635
AtRbohI_Q9SUT8     KPTSFDYKSGQYVFVQCPSVSKFEWHPFSITSSPGDDYLSIHIRQRGDWTEGIKKAFSVV 692
AtRbohJ_Q9LZU9     KPQGFKYKSGMYMFVKCPDISKFEWHPFSITSAPGDEYLSVHIRALGDWTSELRNRFAET 645
```

```
OsRbohA_Q0JJJ9     CEP--PAGG-----------KSGLLRA-----DETTKKILPKLLIDGPYGSPAQDYSKY 539
OsRbohB_Q5ZAJ0     CRPP--TEG-----------ESGLLRADLSKGITDEKARFPKLLVDGPYGAPAQDYREY 719
OsRbohC_Q65XC8     CEP--PVGG-----------KSGLLRA-----DETTKKALPKLLIDGPYGSPAQDYSKY 747
OsRbohD_Q0DHH6     CEAQVSSKKA---TLARLETTII---AD-----GLKEETCFPKVFIDGPFGAPAQNYKKY 634
OsRbohE_Q8S1T0     CEAQVTSKKA---TLSRLETTVV---AD-----AQTEDTRFPKVLIDGPYGAPAQNYKKY 659
OsRbohF_Q0J595     YFV-PSVPR-----------RASFGAL-----GMAEQKSPPRLLVDGPYGAPAQDFRNY 833
OsRbohG_Q69LJ7     YFS-PHLNR-----------RASFSEL-----GATEPRSLPRLLVDGPYGAPAQDFRNY 807
OsRbohH_Q2QP56     CRPP--MNG-----------QSGLLRADCMS--MEHHSRFPKLLIDGPYGAPAQDYWKY 718
OsRbohI_Q2R351     CRPP--TEG-----------KSGLLRAEYDRDGAMTNPSFPKVLIDGPYGAPAQDYKQY 748
AtRbohA_O81209     CKPR--PPD-----------EHRLNRADSKH--WDYIPDFPRILIDGPYGAPAQDYKKF 707
AtRbohB_Q9SBI0     CQLP--STS-----------QSGLFIADIGQA--NNITRFPRLLIDGPYGAPAQDYRNY 661
AtRbohC_O81210     CKPP--PAG-----------VSGLLRADMLH--GANNPDFPKVLIDGPYGAPAQDYKKY 715
AtRbohD_Q9FIJ0     CKPP--TAG-----------KSGLLRAD----GGDGNLPFPKVLIDGPYGAPAQDYKKY 736
AtRbohE_O81211     KDLSTCVIG-----------RSKFSAY-----CNIDMINRPKLLVDGPYGAPAQDYRSY 752
AtRbohF_O48538     CEP--PVGG-----------KSGLLRA-----DETTKKSLPKLLIDGPYGAPAQDYRKY 746
AtRbohG_Q9SW17     SKPP--PVG------------------DMLN--GANSPRFPKIMIDGPYGAPAQDYKKY 661
AtRbohH_Q9FJD6     CEPTQAAAKPKPNSLMRMETRAAGVNPH-----IEESQVLFPKIFIKGPYGAPAQNYQKF 690
AtRbohI_Q9SUT8     CHA--PEAG-----------KSGLLRA-----DVPNQRSFPELLIDGPYGAPAQDHWKY 733
AtRbohJ_Q9LZU9     CEPHQK-SKPSPNDLIRMETRARGANPH-----VEESQALFPRIFIKGPYGAPAQSYQKF 699
```

**NADPH-I**

```
OsRbohA_Q0JJJ9     DVLLLVGLGIGATPFISILKDLLNNIIKMEEEEDAST-DLYPPMGRN--KP----HVDLG 592
OsRbohB_Q5ZAJ0     DVLLLIGLGIGATPLISIVKDVLNHIQGEGSVGTT---------E-------------- 755
OsRbohC_Q65XC8     DVLLLVGLGIGATPFISILKDLINSIIKMEEEEEASG-DLYPPIGRN--KA----HVDLD 800
OsRbohD_Q0DHH6     DILLLIGLGIGATPFISILKDLLNNIKSNGDVQ------STHDAELGC----------- 676
OsRbohE_Q8S1T0     DILLLIGLGIGATPFISILKDLLNNIKSNEEVE------SIHGSEIG------------ 700
OsRbohF_Q0J595     DVLLLVGLGIGATPFISILRDLLNNIKLADELMDLAMET-S----RS------DDSANSF 882
OsRbohG_Q69LJ7     DVLLLVGLGIGATPFISILRDLLNNIKLAEELMDLAMET-S----RS------EDSANSF 856
OsRbohH_Q2QP56     DVLLLIGLGIGATPLISIVKDVLNHIYDDPESAASPHT--TN----------------- 758
OsRbohI_Q2R351     DIVLLVGLGIGATPMISIIKDIINNMRQLDGDLED-------GDG-------------- 786
AtRbohA_O81209     EVVLLVGLGIGATPMISIVSDIINNLKGVEEGSNRRQSPIHNMVT-------------- 752
AtRbohB_Q9SBI0     DVLLLIGLGIGATPLISIIRDVLNNIKNQNSIE----------RG-------------- 696
AtRbohC_O81210     EVVLLVGLGIGATPMISIVKDIVNNIKAKEQAQLNRME-----NG-------------- 755
AtRbohD_Q9FIJ0     DVVLLVGLGIGATPMISILKDIINNMKGPDRDSDI------------------------ 771
AtRbohE_O81211     DVLLLIGLGIGATPFISILKDLLNNSRDEQTDNEFSRSDFSWN-----------SCTSSY 801
AtRbohF_O48538     DVLLLVGLGIGATPFISILKDLLNNIVKMEEHADSIS-DFSRSSEYS--T---------- 793
AtRbohG_Q9SW17     EVVLLIGLGIGATPMISIIKDIINNTETKE--QLSQME-----KG-------------- 699
AtRbohH_Q9FJD6     DILLLVGLGIGATPFISILKDMLNHLKPGIPRSGQKYEGSVGGESIGGDSVSG------- 743
AtRbohI_Q9SUT8     DVVLLVGLGIGATPFVSILRDLLNNIIKQQEQAECIS-GSCSNSNIS--SDHSFSCLNSE 790
AtRbohJ_Q9LZU9     DILLLIGLGIGATPFISILKDMLNNLKPGIPKTGQKYEGSVGGESLGGSSVYGGSSVNGG 759
```

**NADPH-II**

```
OsRbohA_Q0JJJ9      T--LMTITSRPKKILKTTNAYFYWVTREQGSFDWFKGVMNEIADLDQR-NIIEMHNYLTS 649
OsRbohB_Q5ZAJ0      ---PESSSKAKKKPFMTKRAYFYWVTREEGSFEWFRGVMNEVSEKDKD-GVIELHNHCSS 811
OsRbohC_Q65XC8      T--LMRITSKPKRVLKTTNAYFYWVTREQGSFDWFKGVMNEIAELDQR-NIIEMHNYLTS 857
OsRbohD_Q0DHH6      ----------TFKSNGPGRAYFYWVTREQGSFEWFKGVMNDVAESDHD-NVIEMHNYLTS 725
OsRbohE_Q8S1T0      ----------SFKNNGPGRAYFYWVTREQGSFEWFKGVMNDVAESDHN-NIIEMHNYLTS 749
OsRbohF_Q0J595      S--VSTASSNKRRAYRTSRAHFYWVTREPGSFEWFKGVMNEVAEMDKK-GVIELHNYLTS 939
OsRbohG_Q69LJ7      S--VSTASSNKKRAYRTSRAHFYWVTREPLSFEWFKGVMNEVAEMDKK-GVIELHNYLTS 913
OsRbohH_Q2QP56      ---GGGAAAAARRAFMTKRVYFYWCTREEGSFEWFRGVMNEVADRDAGRELIELHNHCTS 815
OsRbohI_Q2R351      ---NDNSVSSSSAAFKTRRAYFYWVTREQGSFEWFRGVMDEVAETDKK-GVIELHNYCTS 842
AtRbohA_O81209      ---PPVSSPSRKSETFRTKRAYFYWVTREQGSFDWFKNVMDEVTETDRK-NVIELHNYCTS 808
AtRbohB_Q9SBI0      ---TNQ---HIKNYVATKRAYFYWVTREQGSLEWFSEVMNEVAEYDSE-GMIELHNYCTS 749
AtRbohC_O81210      ---TSEPQRSKKESFRTRRAYFYWVTREQGSFDWFKNIMNEVAERDAN-RVIEMHNYCTS 811
AtRbohD_Q9FIJ0      ---ENNNSNNNSKGFKTRKAYFYWVTREQGSFEWFKGIMDEISELDEE-GIIELHNYCTS 827
AtRbohE_O81211      T--TATPTSTHGGKKKAVKAHFYWVTREPGSVEWFRGVMEEISDMDCR-GQIELHNYLTS 858
AtRbohF_O48538      G--SNGDTPRRKRILKTTNAYFYWVTREQGSFDWFKGVMNEVAELDQR-GVIEMHNYLTS 850
AtRbohG_Q9SW17      ---SPQEQQGNKETFKTRRAYFYWVTKEQGTFDWFKNIMNEIAERDKS-KVIELHNHCTS 755
AtRbohH_Q9FJD6      ----------GGGKKFPQRAYFFWVTREQASFDWFKGVMDDIAEYDKT-HVIEMHNYLTS 792
AtRbohI_Q9SUT8      A--ASRIPQTQRKTLNTKNAYFYWVTREQGSFDWFKEIMNEIADSDRK-GVIEMHNYLTS 847
AtRbohJ_Q9LZU9      GSVNGGGSVSGGGRKFPQRAYFYWVTREQASFEWFKGVMDDIAVYDKT-NVIEMHNYLTS 818
```

**NADPH-III**

```
OsRbohA_Q0JJJ9      VYEEGDARSALITMLQALNHAKNGVDIVSGTKVRTHFARPNWRKVLSKISSKHPYAKIGV 709
OsRbohB_Q5ZAJ0      VYQEGDARSALIVMLQELQHAKKGVDILSGTSVKTHFARPNWRSVFKKVAVSHENQRVGV 871
OsRbohC_Q65XC8      VYEEGDARSALITMLQALNHAKNGVDIVSGTKVRTHFARPNFKKVLSKIASKHPYAKIGV 917
OsRbohD_Q0DHH6      VYEEGDARSALIAMVQSLQHAKNGVDIVSGSKIRTHFARPNWRKVFSDLANAHQNSRIGV 785
OsRbohE_Q8S1T0      VYEEGDARSALIAMVQSLQHAKNGVDIVSGSRIRTHFARPNWRKVFSDLANAHKNSRIGV 809
OsRbohF_Q0J595      VYEERDARSTLLSMVQALNHAKHGVDIVSGTRVRTHFARPNWKEVFTRIASKHPNSTVGV 999
OsRbohG_Q69LJ7      VYEERDARTTLLSMVQALNHAKHGVDIVSGTRVRTHFARPNWKEVFTRIASKHPNSTVGV 973
OsRbohH_Q2QP56      VYEEGDARSALVTMLQALHHAKNGVDVVSGTRVRTHFARPSWRDVFKRVAVNHQGQRVGV 875
OsRbohI_Q2R351      VYEEGDARSALIAMLQSLNHAKHGVDVVSGTRVKTHFARPNWRNVYKRIALNHRDQRVGV 902
AtRbohA_O81209      VYEEGDARSALITMLQSLNHAKHGVDVVSGTRVMSHFARPNWRSVFKRIAVNHPKTRVGV 868
AtRbohB_Q9SBI0      VYEEGDARSALITMLQSLHHAKSGIDIVSGTRVRTHFARPNWRSVFKHVAVNHVNQRVGV 809
AtRbohC_O81210      VYEEGDARSALIHMLQSLNHAKNGVDIVSGTRVMSHFAKPNWRNVYKRIAMDHPNTKVGV 871
AtRbohD_Q9FIJ0      VYEEGDARVALIAMLQSLQHAKNGVDVVSGTRVKSHFAKPNWRQVYKKIAVQHPGKRIGV 887
AtRbohE_O81211      VYDEGDARSTLIKMVQALNHAKHGVDILSGTRVRTHFARPNWKEVFSSIARKHPNSTVGV 918
AtRbohF_O48538      VYEEGDARSALITMVQALNHAKNGVDIVSGTRVRTHFARPNWKKVLTKLSSKHCNARIGV 910
AtRbohG_Q9SW17      VYEEGDVRSALIRMLQSLNYAKNGLDIVAGTRVMSHFARPNWKNVYKQIAMDHPGANVGV 815
AtRbohH_Q9FJD6      MYEAGDARSALIAMVQKLQHAKNGVDIVSESRIRTHFARPNWRKVFSELSSKHEACRIGV 852
AtRbohI_Q9SUT8      VYEEGDTRSNLLTMIQTLNHAKNGVDIFSGTKVRTHFGRPKWKKVLSKISTKHRNARIGV 907
AtRbohJ_Q9LZU9      MYEAGDARSALIAMVQKLQHAKNGVDIVSESRIRTHFARPNWRKVFSELSNKHETSRIGV 878
```

**NADPH-IV**

```
OsRbohA_Q0JJJ9      FYCGAPVLAQELSKLCHEFNGKCTTKFEFHKEHF 743
OsRbohB_Q5ZAJ0      FYCGEPVLVPQLRQLSADFTHKTNTRFDFHKENF 905
OsRbohC_Q65XC8      FYCGAPVLAQELSDLCHDFNGRCTSKFEFHKEHF 951
OsRbohD_Q0DHH6      FYCGSPTLTKMLRDLSLEFSQTTTTRFHFHKENF 819
OsRbohE_Q8S1T0      FYCGSPTLTKQLKDLSKEFSQTTTTRFHFHKENF 843
OsRbohF_Q0J595      FYCGKPTLAKELKKLSLDMSHKTTTRFHFHKEYF 1033
OsRbohG_Q69LJ7      FYCGAPTLAKELKTLSHEMSHRTGTRFHFHKEYF 1007
OsRbohH_Q2QP56      FFCGDQALTPELRRLAQDFSHKTTTKFVFHKENF 909
OsRbohI_Q2R351      FYCGAPVLTKELRELAQDFSRKTSTKFDFHKENF 936
AtRbohA_O81209      FYCGAAGLVKELRHLSLDFSHKTSTKFIFHKENF 902
AtRbohB_Q9SBI0      FYCGNTCIIGELKRLAQDFSRKTTTKFEFHKENF 843
AtRbohC_O81210      FYCGAPALTKELRHLALDFTHKTSTRFSFHKENF 905
AtRbohD_Q9FIJ0      FYCGMPGMIKELKNLALDFSRKTTTKFDFHKENF 921
AtRbohE_O81211      FYCGIQTVAKELKKQAQDMSQKTTTRFEFHKEHF 952
AtRbohF_O48538      FYCGVPVLGKELSKLCNTFNQKGSTKFEFHKEHF 944
AtRbohG_Q9SW17      FYCGAPVLTKELRQLALEFTHKTSTRFSFHKENF 849
AtRbohH_Q9FJD6      FYCGSPTLVRPLKELCQEFSLESSTRFTFHKENF 886
AtRbohI_Q9SUT8      FYCGVPSLGKELSTLCHEFNQTGITRFDFHKEQF 941
AtRbohJ_Q9LZU9      FYCGSPTLVRPLKSLCQEFSLESSTRFTFHKENF 912
```
